# Supplementary material for: Mixed-methods feasibility study to inform a randomised controlled trial of proton pump inhibitors to reduce strictures following neonatal surgery for oesophageal atresia
Source: BMJ Open. 2023 Apr 20;13(4):e066070. doi: 10.1136/bmjopen-2022-066070 (PMC10124212; doi:10.1136/bmjopen-2022-066070)
Supplement: Supplementary data [file bmjopen-2022-066070supp001.pdf]

TOAST
Treating Oesophageal Atresia to Prevent Stricture

TOAST feasibility study

Parent/Legal Representative Information Sheet

We invite you to take part in a research study

Following repair of oesophageal atresia some babies develop a narrowing at the site of the repair in the oesophagus called a stricture. Reflux is also very common after repair of oesophageal atresia and it is believed that reflux of acid into the oesophagus may make a stricture more likely or more severe.

Antacid medication is sometimes used to supress the acid produced by the stomach and help reduce the risk of strictures. Despite this treatment being used in about half of babies with oesophageal atresia, the evidence for using this medication to reduce the chance of getting a stricture is weak. We don't really know if it works. But we would like to find out because if it does then all babies with oesophageal atresia can be given this treatment and if it does not then we can stop asking parents to give medicine to their baby that has no benefit.

We want to answer the question, "Should babies born with oesophageal atresia all be treated routinely with antacid medication to reduce strictures?" To do this we are planning a clinical trial, which will be the first of its kind, to help inform the future treatment of babies with oesophageal atresia.

- To help find out what parents think about the trial and help to design it, we would like to interview about 25 parents/legal representatives with experience of infants born with oesophageal atresia within the last three years, including those with or without stricture and those who did or did not receive routine antacid medication
- By speaking to parents/ legal representatives we hope to find out whether the trial is acceptable and, if so, how it should be done.
- The interview should take about 30-45 minutes and can be done over the telephone or online (e.g. Zoom). Should you be interviewed, you will receive a £30 Amazon voucher to thank you for your time.

How to contact us

If you have any questions and/or would like to take part in an interview, please contact: Dr Tracy Mitchell Telephone: 07379 105134 Email: tracy.mitchell@liverpool.ac.uk Further information can be found on our website: ***https://www.npeu.ox.ac.uk/toast/parents***

Why are we doing this study?

Babies may suffer reflux after surgery where the acid content of the stomach comes back into the oesophagus and can cause regurgitation of feeds and/or damage to the oesophagus. Sometimes a narrowing of the tube (called a stricture) where the oesophagus was rebuilt may be caused or made worse by reflux. Some surgeons who look after babies with oesophageal atresia use a medication to suppress the acid produced by the stomach even if there are no symptoms of reflux. A major reason for this is to reduce the risk of strictures forming. Despite this being an apparently popular option (about half of babies with oesophageal atresia are treated with this medication) the evidence for using this medication is weak. In fact, some studies of babies with oesophageal atresia have actually found that strictures are more common in babies treated with acid suppression than in those who were not. In addition, there is some suggestion that taking the medicine can increase the chance of certain types of infection. Until we conduct a clinical trial we don't really know the best way to treat babies with oesophageal atresia.

We want to answer the question, "Should babies born with oesophageal atresia all be treated routinely with antacid medication to reduce strictures?" To do this we are planning a two-phased study. Firstly, we will assess the feasibility of the study. We will work with surgeons, other doctors looking after babies with oesophageal atresia, families of such babies and others who are involved in their care to explore what is important to them and whether they would be willing to take part in a trial where we test our question. Secondly, the findings from this will then be used to design a trial where babies with oesophageal atresia are allocated at random to either being given acid suppressing medicine or a placebo (an inactive substance that looks the same as the medicine). This is called a randomised controlled trial and is considered the "Gold Standard" in terms of answering clinical questions like this.

Who can take part?

Parents/legal representatives with experience of a baby with oesophageal atresia, including those with or without stricture and those who did or did not receive routine antacid medication in the last three years.

How can I take part?

If you would like to take part in an interview, please contact Tracy Mitchell or Kerry Woolfall by email or telephone (contact details are on the first page). If two parents/guardians in the same family wish to take part then the interviews will be conducted separately, one at a time.

Before the interview, we will send you an information sheet (like this one) which will be used to invite parents/guardians to take part in the TOAST clinical trial.

During the interview, Tracy will ask you what you think about the information sheet and what improvements you think we could make. We would also appreciate your views on the best way to speak to parents about this research, the design of the study (such as how and when medication is given to babies with reflux), our approach to seeking consent and your views on the parent / family centred things that we plan to observe as outcomes of the trial. With your permission we would like to record the interviews for analysis purposes but all names and identifying information will be removed. Interviews will be digitally recorded which will then be written out by the researcher or a transcription service. After the interview, we will send you a £30 Amazon voucher to thank you for your time.

The views of parents with experience infants born with oesophageal atresia are very important to make sure we design this study in a way that is acceptable to parents/guardians and ensure that they understand the study information given to them in such stressful circumstances.

Taking part is completely optional and you can change your mind about being part of the study at any time by contacting the study team.

If you have any questions before deciding to take part, please do not hesitate to contact us. The study results will be made available on the study website when the study is finished.

Are there any risks in taking part?

This is a low risk study. The majority of questions will be about the proposed clinical trial. However, at the beginning of the interview we will be inviting you to discuss your child's experiences, such as when was your child's oesophageal atresia first identified or diagnosed. And did your child experience a narrowing of their oesophagus following this surgery? Such personal questions about your child's experience may be upsetting for some. If you would rather not answer such questions, please let the interviewer know. You can decide not to answer a question at any time. The interview can also be paused or stopped at any time you wish. Details of additional support such as the TOFS (Tracheo-Oesophageal Fistula Support) group for parents are provided on the last page of this information sheet.

Who is involved in this study?

The study is being funded by the National Institute for Health Research (NIHR) which is the research arm of the NHS. Mr Nigel Hall (University of Southampton) and Mr Iain Yardley (Evelina Children's Hospital) are the TOAST Study Chief Investigators. At a later stage, it is planned that the the National Perinatal Epidemiology Unit, Clinical Trials Unit (NPEU CTU) at the University of Oxford (which has been central to previous UK-wide surveys of babies with Oesophageal Atresia) will conduct the trial. Dr Kerry Woolfall (University of Liverpool) is leading this element of the study. The TOAST research team are qualified to do this study because they have all the specialties and skills needed. Members of team have a lot of experience in caring for children with oesophageal atresia and are very active in health research. Parents of children oesophageal atresia and TOFS have been involved in the development of this study.

How will my data be used and what happens if I want to stop taking part?

The University of Liverpool is the sponsor for this study based in the United Kingdom. We will be using information you provide us with during interviews in order to undertake this study and will act as the data controller for this study. This means that we are responsible for looking after your information and using it properly. The University of Liverpool will keep identifiable information about you for 10 years after the study has finished.

Your rights to access, change or move your information are limited, as we need to manage your information in specific ways in order for the research to be reliable and accurate. Participation in the study is voluntary and you are free to withdraw without explanation up until your data has been anonymised (approximately a week after the interview). All identifiable information removed and saved anonymously.

The University processes personal data as part of its research and teaching activities in accordance with the lawful basis of 'public task', and in accordance with the University's purpose of "advancing education, learning and research for the public benefit." Under UK data protection legislation, the University acts as the Data Controller for personal data collected as part of the University's research. Dr Kerry Woolfall, acts as the Data Processor for this study, and any queries relating to the handling of your personal data can be sent to Dr Kerry Woolfall, University of Liverpool (Tel: 0151 794 4634).

Further information on how your data will be used can be found in the table below.

How will my data be collected?	Digital recording of interviews.
How will my data be stored?	Password protected data files.
How long will my data be stored for?	10 years
What measures are in place to protect the security and confidentiality of my data?	Any identifiable information will be taken out of the interviews when written out and each participant will be assigned a number. Consent forms will be securely stored.
Will my data be anonymised?	All identifiable information will be removed, and a number assigned to each participant.
How will my data be used?	Findings will be written up for the funders NIHR. Publication will be sought with peer reviewed journals.
Who will have access to my data?	Tracy Mitchell and Kerry Woolfall
Will my data be archived for use in other research projects in the future?	No
How will my data be destroyed?	Shredded or deleted after 10 years

Mitchell TK, *et al. BMJ Open* 2023; 13:e066070. doi: 10.1136/bmjopen-2022-066070

What if there is a problem?

If you are unhappy, or if there is a problem, please feel free to let us know by contacting **Kerry Woolfall, Telephone: 0151 794 4634** and we will try to help. If you remain unhappy or have a complaint which you feel you cannot come to us with then you should contact the Research Ethics and Integrity Office at *ethics@liv.ac.uk*. When contacting the Research Ethics and Integrity Office, please provide details of the name or description of the study (so that it can be identified), the researcher(s) involved, and the details of the complaint you wish to make. The University strives to maintain the highest standards of rigour in the processing of your data. However, if you have any concerns about the way in which the University processes your personal data, it is important that you are aware of your right to lodge a complaint with the Information Commissioner's Office by calling 0303 123 1113. For NHS service advice or support please contact: Patient Advice and Liaison Services (PALS) services. Go to *www.nhs.uk* to find your local PALS contact details. There is also the TOFS (Tracheo-Oesophageal Fistula Support) support group for parents:

www.tofs.org.uk/about-us.aspx

Telephone +44 (0)115 961 3092

Email: *info@tofs.org.uk*

Who has reviewed the study?

The study has been reviewed by the University of Liverpool Research Ethics Committee (REC Ref. 8510) who have agreed that the study is being conducted in a correct and appropriate manner.

Thank you for your time.
We are very grateful that you are considering taking part in this study.

TOAST Study Team
NPEU Clinical Trials Unit, University of Oxford,
Old Road Campus, Headington, Oxford, OX3 7LF.
01865 617919
toast@npeu.ox.ac.uk
www.npeu.ox.ac.uk/toast

TOAST is funded by the National Institute for Health Research (NIHR) Health Technology Assessment programme (project reference 131136). The views expressed are those of the author(s) and not necessarily those of the NIHR or the Department of Health and Social Care.
