# Supplementary material for: Mixed-methods feasibility study to inform a randomised controlled trial of proton pump inhibitors to reduce strictures following neonatal surgery for oesophageal atresia
Source: BMJ Open. 2023 Apr 20;13(4):e066070. doi: 10.1136/bmjopen-2022-066070 (PMC10124212; doi:10.1136/bmjopen-2022-066070)
Supplement: Supplementary data [file bmjopen-2022-066070supp002.pdf]

TOAST Practitioner Questionnaire

Thank you for agreeing to help us with a study assessing the feasibility of a proposed RCT into the use of proton pump inhibitor (PPI) medication (Omeprazole) to reduce anastomotic stricture formation following oesophageal atresia repair. We would like to explore your attitudes and opinions about the study concept, the trial design and ways in which we can make the project more likely to succeed. This questionnaire, which will take approximately 15 minutes to complete, will explore your views on the proposed trial design, including questions to assess your views on trial acceptability, the inclusion/exclusion criteria, and your willingness to be involved in the TOAST trial.

Your responses are important to us and will help to refine the trial protocol. Please note that by completing this survey you are giving permission for your responses to be included in the NIHR funded TOAST study. All information will be held by the University of Liverpool, anonymised and stored securely in compliance with UK GDPR.

If you have any questions, please email Dr Tracy Mitchell: Tracy.Mitchell@liverpool.ac.uk or Dr Kerry Woolfall: woolfall@liverpool.ac.uk. Further information about the study can be found here: https://www.npeu.ox.ac.uk/toast/clinicians

It is recommended that you download the 'Treatment plan for treating babies with symptoms of reflux' flowchart before commencing this questionnaire to be able to see the text more clearly, especially if completing this questionnaire on your mobile phone: https://www.npeu.ox.ac.uk/assets/downloads/toast/pathway.pdf

Once you have read and understood each statement, please click on the box to indicate consent.

* Required

1. I confirm that I have read and understood the information sheet. I have had the opportunity to consider the information, ask questions and have these answered satisfactorily *

   Check all that apply.

   ☐ Yes

2. I agree to participate in the questionnaire *

   Check all that apply.

   ☐ Yes

Mitchell TK, *et al. BMJ Open* 2023; 13:e066070. doi: 10.1136/bmjopen-2022-066070

3. I understand that my responses will remain confidential and will not be used for any other purpose *

   Check all that apply.

   - [ ] Yes

4. I understand that I am free to withdraw from the study at any time without reason, and if so, all of my information will be disposed of *

   Check all that apply.

   - [ ] Yes

5. I understand that the results from the study may be published in an academic journal and that it will be kept anonymous *

   Check all that apply.

   - [ ] Yes

6. I agree to my data being stored on a computer at University of Liverpool for 10 years in line with the standard university procedure *

   Check all that apply.

   - [ ] Yes

Please read this outline of the proposed Treating Oesophageal Atresia to Prevent Stricture (TOAST) trial before completing the questionnaire

As you know, babies born with oesophageal atresia (OA) may have gastro-oesophageal reflux after surgery to repair their oesophagus. The link between reflux and the development of anastomotic stricture is contentious. Some surgeons who look after babies with oesophageal atresia use proton pump inhibitor (PPI) medication to suppress the acid produced by the stomach even if there are no symptoms of reflux. A major reason for this is to reduce the risk of strictures forming. Despite this being an apparently popular option (about half of babies with oesophageal atresia are treated with PPI medication) the evidence for using this medication is weak. In fact, some studies of babies with oesophageal atresia have actually found that strictures are more common in babies treated with PPI medication than in those who were not. In addition, there is some suggestion that taking the PPI medication can increase the chance of certain types of infection.

The primary objective of the Treating Oesophageal Atresia to Prevent Stricture (TOAST) study is to address the research question 'In babies born with oesophageal atresia does routine use of PPI medication reduce the severity or incidence of anastomotic stricture compared to placebo?'

To do this we aim to perform a multicentre double blinded randomised placebo-controlled trial (with integral internal pilot and health economic evaluation) of babies with type C oesophageal atresia (i.e., only those with distal fistula) in which half will receive PPI from the time of repair until 1 year of age and the other half will receive placebo. The primary outcome of the TOAST study is to assess the severity of anastomotic stricture through the number of dilatations performed within the first year of life, and we intend to measure a range of other relevant and meaningful outcomes for up to two years. We have selected type C only so as to have a reasonably homogenous population and because this represents the majority of babies born with OA.

The research will take place in specialist U.K. neonatal surgical units and in participant homes; it is expected that approximately 12-15 units will take part. An 18-month internal pilot phase incorporates "stop-go" criteria to evaluate feasibility of recruitment and other trial processes. The trial aims to recruit 211 infants in 90 months from the recruitment start date, equating to around 3-4 infants per month across all participating sites. Eligible infants will be recruited early in life, around the time of their surgical repair, so that the trial intervention can commence as soon as possible after surgical repair. Where a diagnosis of oesophageal atresia is made antenatally, parents may be approached regarding the trial before birth. Eligible infants will be identified by the clinical care team and recruited by appropriately trained, delegated individuals. These infants will be randomised to receive PPI medication or a placebo, daily, until one year of age.

Trial data collection will be from trial entry until 24 months of age, including screening, consent, randomisation and follow-up. Outcome information will be collected by case report forms (CRFs), paper and electronic, with clinical data collection from medical records at hospital sites and parent reported outcomes. Participant compliance with administering PPI medication or placebo will be performed via a parent report App. The App may also be used for communication to parents about data collection, reportable safety events and other trial communications. Data will be collected when the child is 3, 6, 9, 12, 18, and 24 months of age on primary care contacts, out of pocket expenses, time away from work, and parental health related quality of life using the EuroQol EQ-5D-5L questionnaire. Children's health related quality of life using the parent completed PedsQL questionnaire will be collected at 24 months.

We have designed this trial to be as pragmatic as possible, so that data are recorded during routine hospital visits as much as possible and there is little interference with your standard follow-up pathways. There key points that are important to the successful running of the trial are as follows:

- The trial will be blinded so that none of surgeons, nurses, nor parents will know which treatment is being given.
- The dose of PPI we plan to use will be 1mg/kg/day. During the trial the use

of other gastric acid suppression medication will generally not be allowed (e.g., other PPI or ranitidine) since we wish to maintain separation between the trial groups. To allow for symptoms of reflux in babies to be treated we have designed a pathway for escalating treatment of reflux symptoms for use in this trial. This will also prevent inadvertent overdosing with PPI.

• During the trial, we will be discouraging the use of 'routine' dilatations / calibrations, reserving these only for when an infant is symptomatic. However, we will be leaving the definition of symptomatic to clinician judgement.

The schedule of events for the proposed trial are summarised in the flow chart below.

Flow chart: Treating Oesophageal Atresia to prevent STricture (TOAST)

Inclusion criteria

- Infants with oesophageal atresia with distal trache-oesophageal fistula undergoing primary repair at the first operative intervention in the newborn period
- Written informed parental consent

Exclusion criteria

- Infants undergoing staged repair or delayed primary repair or requiring emergency ligation of trache-oesophageal fistula with primary repair later
- No realistic prospect of survival

Randomisation (1:1 allocation ratio)

Routine anti-reflux medication

Esomeprazole 0.5mg/kg whilst infants are nil by mouth, followed by omeprazole 1mg/kg

Given once daily from trial entry for one year

OR

Placebo

Given once daily from trial entry for one year

Primary Outcome

Severity of anastomotic stricture
(defined as number of dilatations performed to one year of age)

Secondary Outcomes

Measured at 3, 6, 9, 12, 18 and 24 months after trial entry

- Incidence of anastomotic stricture (0 vs 1 or more dilatations)
- Other complications directly related to oesophageal atresia
- Adverse events
- Presence of symptoms of gastro-oesophageal reflux
- Need for treatment of reflux
- Growth
- Respiratory symptoms
- Oral intake
- Episodes of infection
- Mortality
- Maternal Health Related Quality of Life (EQ-5D-5L)
- Child Health Related Quality of Life (PedsQL; parent reported, collected at age 24 months)
- Healthcare and societal costs
- Incremental cost per dilation prevented
- Other meaningful outcomes proposed during the feasibility phase

Longer-term follow up

Permission will be requested to access trial participants' healthcare and educational records for the measurement of longer-term outcomes using routinely collected data. This activity would be as part of a separately funded study.

TOAST flow chart 280721

Part 1 Background Information

7. Are you actively involved in the pre/post-surgical care of children with oesophageal atresia (OA) in the United Kingdom? *

Mark only one oval.

- ( ) Yes *Skip to question 8*
- ( ) No *Skip to section 4 (Thank you for your interest in the TOAST study. Unfortunately, this study is only involving those actively involved in the pre or post surgical care of children with oesophageal atresia (OA) in the United Kingdom.)*

Thank you for your interest in the TOAST study. Unfortunately, this study is only involving those actively involved in the pre or post surgical care of children with oesophageal atresia (OA) in the United Kingdom.

8. Which UK hospital do you work at? *

____

9. What is your job role? *

Mark only one oval.

- ( ) Surgeon *Skip to question 11*
- ( ) Neonatologist *Skip to question 11*
- ( ) Neonatal surgical specialist nurse *Skip to question 11*
- ( ) Other *Skip to question 10*

10. Please type your job role here *

____

11. Approximately how many babies with oesophageal atresia do you treat/are under your own individual care each year? *

____

12. Do you have any experience of recruitment to clinical trials? *

Mark only one oval.

- ( ) Yes *Skip to question 13*
- ( ) No *Skip to question 14*

13. How many years' experience do you have in recruiting to clinical trials? *

____

14. Do you routinely administer or prescribe prophylactic proton pump inhibitors following surgery in ALL babies with type C oesophageal atresia under your care? *

Mark only one oval.

- ( ) Yes *Skip to question 15*
- ( ) No *Skip to question 16*

15. Please can you explain your reasons for prescribing prophylactic PPIs following surgery? *

____
____
____
____
____

Skip to question 17

Supplemental material
BMJ Publishing Group Limited (BMJ) disclaims all liability and responsibility arising from any reliance placed on this supplemental material which has been supplied by the author(s)
BMJ Open

16. Please can you explain your reasons for not prescribing prophylactic PPIs following surgery? *

____
____
____
____
____

Part 2: Inclusion and exclusion criteria

The primary objective of the Treating Oesophageal Atresia to Prevent Stricture (TOAST) study is to address the research question 'In babies born with oesophageal atresia does routine use of PPI medication reduce the severity or incidence of anastomotic stricture compared to placebo?' TOAST is a multicentre double blinded randomised placebo-controlled trial of babies with type C oesophageal atresia (i.e., only those with distal fistula) in which half will receive PPI from the time of repair until 1 year of age and the other half will receive placebo.

Our proposed inclusion criteria are:
- Infants with OA with distal tracheoesophageal fistula (TOF) undergoing primary repair at the first operative intervention.
- Written parental informed consent

17. How satisfied are you with these inclusion criteria? *

Mark only one oval.

- Very satisfied
- Satisfied
- Neutral
- Not satisfied
- Not at all satisfied

18. Do you have any comments or suggested changes for proposed inclusion criteria?

____
____
____
____
____

Mitchell TK, *et al. BMJ Open* 2023; 13:e066070. doi: 10.1136/bmjopen-2022-066070

Our proposed exclusion criteria are:

- Infants undergoing staged repair, delayed primary repair or requiring emergency ligation of tracheoesophageal atresia with primary repair later.
- Infants with no realistic chance of survival beyond the new-born period.

19. How satisfied are you with these exclusion criteria? *

Mark only one oval.

- Very satisfied
- Satisfied
- Neutral
- Not satisfied
- Not at all satisfied

20. Do you have any comments or suggested changes for the proposed exclusion criteria?

Part 3: Trial question and acceptability

21. Do you think the proposed trial is addressing an important research question? *

Mark only one oval.

- Yes *Skip to question 22*
- No *Skip to question 23*

Mitchell TK, *et al. BMJ Open* 2023; 13:e066070. doi: 10.1136/bmjopen-2022-066070

22. Please explain why you think that the proposed trial is addressing an important research question? *

____
____
____
____
____

Skip to question 24

23. Please explain why you think that the proposed trial is not addressing an important research question *

____
____
____
____
____

The proposed intervention is esomeprazole (0.5mg/kg intravenously) once daily whilst infants are nil by mouth followed by omeprazole (1mg/kg orally) once daily until one year of age.

24. How acceptable do you think the use of this PPI medication is in the intervention arm of the TOAST trial? *

Mark only one oval.

- Very acceptable
- Acceptable
- Neutral
- Not acceptable
- Not at all acceptable

25. How acceptable do you think the dose of 1mg/kg of omeprazole orally once daily is? *

Mark only one oval.

- Very acceptable
- Acceptable
- Neutral
- Not acceptable
- Not at all acceptable

26. How acceptable do you think the duration of one year for intervention delivery is? *

Mark only one oval.

- Very acceptable
- Acceptable
- Neutral
- Not acceptable
- Not at all acceptable

27. How acceptable would you find administering the PPI medication to babies in your care? *

Mark only one oval.

- Very acceptable
- Acceptable
- Neutral
- Not acceptable
- Not at all acceptable

28. Please elaborate on your answers for the proposed intervention medication

____
____
____
____
____

The comparator to esomeprazole/omeprazole is a matched placebo (e.g., saline/cellulose/water solution with no active ingredient) given intravenously once daily whilst infants are nil by mouth followed by oral administration, once daily, until one year of age.

29. How acceptable do you think the use of a placebo is in the comparator arm of the TOAST trial? *

Mark only one oval.

- Very acceptable
- Acceptable
- Neutral
- Not acceptable
- Not at all acceptable

30. How acceptable would you find administering a placebo to babies in your care? *

Mark only one oval.

- Very acceptable
- Acceptable
- Neutral
- Not acceptable
- Not at all acceptable

31. Please elaborate on your answers for the proposed placebo

____

____

____

____

____

32. Do you think the TOAST trial is practically possible to conduct? *

Mark only one oval.

- Yes
- No

33. How acceptable do you think it is to conduct TOAST Trial? *

Mark only one oval.

- Very acceptable
- Acceptable
- Neutral
- Not acceptable
- Not at all acceptable

34. How acceptable would you find randomising babies with OA under your care into the TOAST trial? *

Mark only one oval.

- Very acceptable
- Acceptable
- Neutral
- Not acceptable
- Not at all acceptable

35. Please elaborate on any of your responses above

____

____

____

____

____

36. How acceptable do you feel it is to give PPI medication to half the babies who take part in this study? *

Mark only one oval.

- Very acceptable
- Acceptable
- Neutral
- Not acceptable
- Not at all acceptable

37. How acceptable do you think it is to NOT give PPI medication to half the babies in this study? *

Mark only one oval.

- Very acceptable
- Acceptable
- Neutral
- Not acceptable
- Not at all acceptable

Supplemental material BMJ Publishing Group Limited (BMJ) disclaims all liability and responsibility arising from any reliance placed on this supplemental material which has been supplied by the author(s) BMJ Open

38. Please elaborate on your answers about the acceptability of giving/not giving PPI medication to half the babies in this study

____

____

____

____

____

Treatment plan for babies with symptoms of reflux

We have developed an escalating treatment plan (please see below, or if you did not download a copy before starting this questionnaire, you can this link: https://tinyurl.com/38n4wjtv) for babies with symptoms of reflux drawing on the best available evidence for the management of reflux. Babies with reflux symptoms would first be managed with changing feed frequencies and positioning advice escalating to feed thickener (if formula feed) or Gaviscon (if breast fed) and only allowed to have additional medication (e.g., omeprazole) if these fail and symptoms are felt to be having a significant impact.

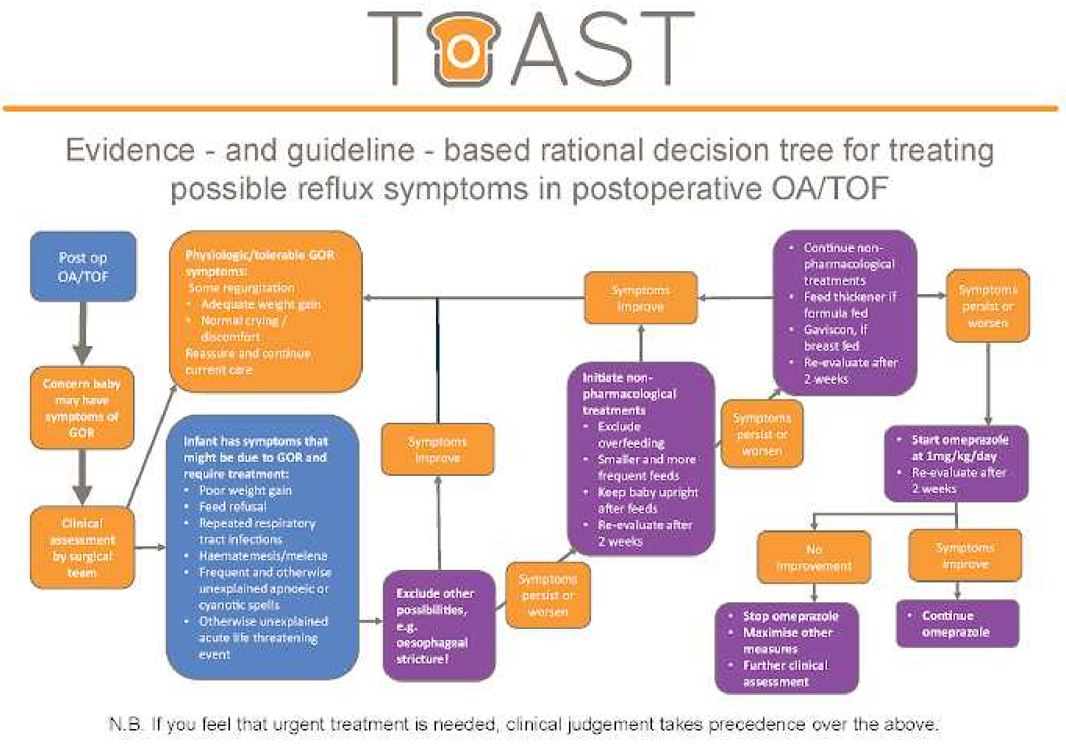

Mitchell TK, *et al. BMJ Open* 2023; 13:e066070. doi: 10.1136/bmjopen-2022-066070

39. How acceptable do you find this escalating treatment plan for babies under your care who have symptoms of reflux in the trial? *

Mark only one oval.

- Very acceptable
- Acceptable
- Neutral
- Not acceptable
- Not at all acceptable

40. Would you be happy to follow this escalating treatment plan for babies under your care who have symptoms of reflux in the trial? *

Mark only one oval.

- Yes
- No

41. Please elaborate

____
____
____
____
____

42. Does the pathway to managing reflux for babies in the trial address any concerns or raise any concerns for you? *

Mark only one oval.

- Yes
- No

Supplemental material
BMJ Publishing Group Limited (BMJ) disclaims all liability and responsibility arising from any reliance placed on this supplemental material which has been supplied by the author(s)
BMJ Open

43. Please elaborate and include whether anything was unclear or missing from the treatment pathway flow chart, or any changes that you would like to suggest.

Part 4: Trial outcomes and follow up

As we have discussed, in the TOAST study we want to find out if routinely giving babies PPI suppression medication after repair of oesophageal atresia will reduce the incidence or severity of oesophageal stricture. To do this we will collect information on outcomes such as those listed below:

Mitchell TK, *et al. BMJ Open* 2023; 13:e066070. doi: 10.1136/bmjopen-2022-066070

TOAST TRIAL: OUTCOMES

Primary Outcome

1. Severity of anastomotic stricture
• The number of dilations performed within one year of trial entry (Measured at 3, 6, 9, 12 months).

Secondary Outcomes (Measured up to the first two years of life)

2. Incidence of anastomotic stricture
• Whether the baby has any dilatation or none in the first year / 2 years of their life.
3. All-cause mortality (up to 12 and 24 months)
• Whether the baby survived to a certain time point (usually time point at months/years) or to a specific event (e.g., hospital discharge).
4. Number of complications directly related to oesophageal atresia post-surgery
• Anything that is directly related to oesophageal atresia or its repair, for instance anastomotic leak or recurrent fistula.
5. Adverse events
• A general term used to describe things that don't go as planned but aren't included in other outcomes.
6. Presence of symptoms of gastro-oesophageal reflux
• Symptoms that may be due to reflux and reported by parents (e.g., using a reflux symptom score, measured every three months).
7. Treatment of reflux symptoms
• Whether the baby is given any sort of medication to treat reflux symptoms (e.g., feed thickener, Gaviscon, Omeprazole).
8. Weight (standard deviation score)
• To monitor the baby's growth during the first year of their life (recorded by weight, length and head circumference at certain time points).
9. Respiratory symptoms
• Presence of respiratory symptoms such as infections, chronic cough (but not the usual TOF cough) or any other symptoms that a doctor felt required investigation or treatment.
10. Oral feed intake
• How the baby is progressing with advancing from milk feeds onto solids at 12 and 24-months using a validated measure (e.g., IDDSI score).
11. Number of courses of antibiotics prescribed for episodes of infection
• (including respiratory infections).
12. Maternal health related quality of life (EQ-5D-5L)
• A measure of a mother's quality of life using a specially designed questionnaire to understand how their baby's health may impact on their health and quality of life.
13. Child health related quality of life (PedsQL; Parent reported, collected at age 24 months)
• A measure of the baby's quality of life, as reported by a parent. This is measured using a specially designed questionnaire and is done at 2 years of age because this is the youngest age that it can reliably be done.

Mitchell TK, et al. BMJ Open 2023; 13:e066070. doi: 10.1136/bmjopen-2022-066070

44. Please rank three outcomes that you feel are most important to be measured in the TOAST trial in order of importance. The first most important outcome for the TOAST study is: *

Mark only one oval.

- Severity of anastomotic stricture
- Incidence of anastomotic stricture
- All-cause mortality
- Number of complications directly related to oesophageal atresia post-surgery
- Adverse events
- Presence of symptoms of gastro-oesophageal reflux
- Treatment of reflux symptoms
- Weight
- Respiratory symptoms
- Oral feed intake
- Number of courses of antibiotics prescribed for episodes of infection
- Maternal health related quality of life
- Child health related quality of life

Mitchell TK, *et al. BMJ Open* 2023; 13:e066070. doi: 10.1136/bmjopen-2022-066070

45. The second most important outcome for the TOAST study is: *

Mark only one oval.

- Severity of anastomotic stricture
- Incidence of anastomotic stricture
- All-cause mortality
- Number of complications directly related to oesophageal atresia post-surgery
- Adverse events
- Presence of symptoms of gastro-oesophageal reflux
- Treatment of reflux symptoms
- Weight
- Respiratory symptoms
- Oral feed intake
- Number of courses of antibiotics prescribed for episodes of infection
- Maternal health related quality of life
- Child health related quality of life

46. The third most important outcome for the TOAST study is: *

Mark only one oval.

- Severity of anastomotic stricture
- Incidence of anastomotic stricture
- All-cause mortality
- Number of complications directly related to oesophageal atresia post-surgery
- Adverse events
- Presence of symptoms of gastro-oesophageal reflux
- Treatment of reflux symptoms
- Weight
- Respiratory symptoms
- Oral feed intake
- Number of courses of antibiotics prescribed for episodes of infection
- Maternal health related quality of life
- Child health related quality of life

47. Are there any other outcomes that you think are important primary or secondary outcomes to measure for this trial that are not included in the list above?

Mark only one oval.

- Yes *Skip to question 48*
- No *Skip to question 51*

48. Please provide your suggestion for an additional outcome here and state whether it is a primary or secondary outcome *

49. Please provide your suggestion for an additional outcome here and state whether it is a primary or secondary outcome

50. Please provide your suggestion for an additional outcome here and state whether it is a primary or secondary outcome

Mitchell TK, *et al. BMJ Open* 2023; 13:e066070. doi: 10.1136/bmjopen-2022-066070

Follow up is currently planned to occur at 3, 6, 9 and 12 months to coincide with routine clinic visits and some will be measured though parental reports via an App. You will be free to see these infants more often should you wish. This will include asking specifically about the treatment of anastomotic strictures and more generally about other symptoms and adverse effects.

51. Is there anything you feel should be specifically addressed in follow up? *

Mark only one oval.

- Yes *Skip to question 52*
- No *Skip to question 53*

52. Please elaborate *

____
____
____
____
____

Part 5: Recruitment and consent

We propose that babies will be randomised into the medication or placebo group within 72 hours of their oesophageal surgery, when their condition has stabilised.

53. How acceptable do you think it is to approach parents to discuss the trial at this point in time? *

Mark only one oval.

- Very acceptable
- Acceptable
- Neutral
- Not acceptable
- Not at all acceptable

Mitchell TK, *et al. BMJ Open* 2023; 13:e066070. doi: 10.1136/bmjopen-2022-066070

54. Please elaborate

55. Would you have any concerns about approaching parents to seek consent for their baby's inclusion in TOAST? *

Mark only one oval.

- Yes
- No

56. Please elaborate

57. How concerned are you about the ability to retain participants in the TOAST trial? *

Mark only one oval.

- Not at all concerned
- A little concerned
- Very concerned

Mitchell TK, et al. BMJ Open 2023; 13:e066070. doi: 10.1136/bmjopen-2022-066070

58. If you have any concerns about the retention of participants in the TOAST trial and/or any suggestions to help address concerns about the retention of participants, please elaborate below

59. Do you envisage any practical or logistical challenges in delivering TOAST at your unit? *

Mark only one oval.

- Yes
- No

60. Please elaborate

Part 6: Overall acceptability and training

Mitchell TK, *et al. BMJ Open* 2023; 13:e066070. doi: 10.1136/bmjopen-2022-066070

61. Overall, how acceptable do you find the proposed TOAST trial? *

Mark only one oval.

- Very acceptable
- Acceptable
- Neutral
- Not acceptable
- Not at all acceptable

62. Please elaborate

____
____
____
____
____

63. Is there anything specific that you would suggest we include in the TOAST site training package? (e.g., training content, who should be trained, whether any online resources would be needed in addition to site initiation visit) *

____
____
____
____
____

64. Do you have any other comments or concerns about the TOAST trial? *

Mark only one oval.

- Yes *Skip to question 65*
- No *Skip to section 27 ()*

65. Please note any other comments or concerns about the TOAST trial here

We plan to conduct further research into clinicians' attitudes to the trial via more in depth focus group or telephone interview discussions. These will be held online via Zoom or a similar platform. Please add your contact details if you would like to register interest in taking part in a focus group or interview. Your details will not be used for any other purposes and will be stored separately from questionnaire data

First name and email address

66. First name

67. Email address

Thank you for your participation in this survey.

This content is neither created nor endorsed by Google.

Google Forms

Mitchell TK, *et al. BMJ Open* 2023; 13:e066070. doi: 10.1136/bmjopen-2022-066070
