# Supplementary material for: Mixed-methods feasibility study to inform a randomised controlled trial of proton pump inhibitors to reduce strictures following neonatal surgery for oesophageal atresia
Source: BMJ Open. 2023 Apr 20;13(4):e066070. doi: 10.1136/bmjopen-2022-066070 (PMC10124212; doi:10.1136/bmjopen-2022-066070)
Supplement: Supplementary data [file bmjopen-2022-066070supp003.pdf]

TOAST Parent interview topic guide

Please note: ***Italic text indicates instruction for researcher and will not be read to participant***

Intro: My name is xxx and I am a researcher from the University of xxx. Many thanks for agreeing to help us with the design of the TOAST study.<br>Before we begin the interview I need to obtain your consent for the study is that ok? (*Refer to instructions at top of Participant Consent Form (re: reading 8 statements, sending a copy and consent for audio recording of this discussion).*

- **Obtain consent here**

You can stop the interview at any time. Before we start do you have any questions?<br>Have you had chance to look at the draft participant information sheet I sent to you for the TOAST trial? (If no- read through sheet with parent)

**Section one- Parent details and experience**	
1.1	For administrative purposes: your age, occupation, first part of post code, ethnic background, number and ages of children, where saw the study advertised.
1.2	My notes from when you registered interest in taking part in this study state that your child was born with oesophageal atresia (*insert month and year*). Is that correct?<br>I have a few questions about your child's experience is that ok?<br>*Explore:*<br>When was your child's oesophageal atresia first identified or diagnosed?<br>When did your child have surgery to reconstruct their oesophagus? (Days or weeks after birth)<br>What treatments or medication did your child receive after this surgery? (Prompt; explore if they received gastric acid suppression medication or if it had been discussed by the doctor/surgeon?)<br>Did your child experience a narrowing of their oesophagus following this surgery? *If yes,* could you tell me a little bit about this? How did the stricture impact on your child? *If relevant,* how many dilations has your child had?<br>Has your child had (or expected to have) treatments for other conditions, which may have impacted upon the oesophageal surgery?<br>Do you access support groups, for example, online groups such as TOFS? (If yes: explore if the group have provided any useful advice about treatments or medication?)
	**We are in the process of developing a type of study called a clinical trial. Have you ever heard of a clinical trial before?**<br>*Explain:* This is a type of medical research which provides information on the safety and effects of a drug or medical device (for example, a needle or line used to administer drugs). They are used to find out the best way to treat patients in the future. Trials are carried out to test many different medicines or treatments for children. Some test medicines or treatments which we think will be low risk, because doctors have already been using them for some time. Some trials test new medicines or ones where we don't know exactly what effects they will have in a particular situation.

	**Have you ever been asked if your child could take part in a clinical trial?** *If yes,*<br>Could you tell me a little more about that?<br>What was the trial looking at?<br>At what point where you approached and asked if you would give consent for your child to take part in the trial?<br>Could you tell me a bit more about what happened? How did you feel at the time?
**Section two- Baseline Knowledge of TOAST**	
2.1	Have you had chance to look at the draft participant information sheet I sent to you for the proposed TOAST study, which is a clinical trial (If no- read through sheet with parent)?
2.2	Based on the participant information sheet please describe your understanding of what the TOAST study is aiming to do?
**Section three- TOAST**	
3.1	**Talk though the draft trial PIL** *Prompt:* Is each of these sections clear? Is there anything we need to add or change?
3.2	Looking at the information sheet, are there any parts of the study design that you think parents may find difficult to understand?
3.3	What would you think if someone approached you about a study like this, explained to you whilst your child was in NICU?<br>What would be your initial thoughts about this proposed study?<br>When do you think would be best time to be approached about the study? (*Prompt, after initial response explore views on time frame proposed*).
3.4	Who do you think would be the best person to approach parents to discuss the TOAST study *(explore views on it being a research nurse, doctor, surgeon)?*
3.5	Would you have any concerns about the proposed TOAST study?
3.6	Would you have any questions about the TOAST study?
3.7	How much time would you need to consider the information before making a decision about the TOAST study?
3.8	For babies who are sent home from hospital, parents will be asked to give their child the gastric acid suppression medication every day as part of the TOAST trial. We are designing an app which will send reminders to parents to help them remember to give the medication<br>*(Prompt explore thoughts on this, timing of notification, how many per day and for how long, storage of medication in fridge and anything parents think might help stop people dropping out of the trial over time, preparation of meds, expiry dates (although we are limited on this), whether gave medication or not, dosing with weight changes, follow up at clinic at 3,6,9,12 months and additional questionnaires)*

Mitchell TK, *et al. BMJ Open* 2023; 13:e066070. doi: 10.1136/bmjopen-2022-066070

3.9	The information sheet describes how each parent would be approached and permission would be sought for their baby's involvement in the TOAST trial. If informed consent is provided, their child will be randomly allocated to either group A (no gastric acid suppression medication) or group B (Gastric acid suppression medication).<br><br>An alternative approach is called 'opt out' consent. This is when all eligible babies are automatically randomly allocated to either group A of group B following their surgery. There will be posters up on the walls of NICU explaining the trial and then parents will be approached with an information sheet and asked if they would like to opt their baby out of the trial. If they 'opt out' then no further information is collected from that point forward and their baby will receive whatever the usual treatment is in that NICU.<br><br>*(Check understanding and clarify any queries)*<br><br>What are your thoughts about an opt out approach for the TOAST trial?<br><br>Do you think the TOAST trial should use an informed consent or opt out approach? *(Explore reasons).*
	Does your child currently take any antacid medications?<br><br>In TOAST, if participating children are given additional gastric acid suppression medication this will make the results hard to interpret. So they would be given anti-reflux and other medications first (see flow chart) and only allowed to have additional medication (e.g. omeprazole) if these fail. How would you feel about that? Would that influence your views on taking part in the study? *(Explore any information that parents feel we should prioritise in the trial PIS)*
3.10	How acceptable do you feel it is to give anti reflux medication to half the babies who take part in this study?
3.11	How acceptable do you think it is to not give anti reflux medication to half the babies in this study?
3.12	What are your thoughts about the suggested pathway to managing symptoms of reflux flowchart? *Explore if:* it addressed any concerns, raised any concerns they may not have had, is it easy to understand? Anything unclear or missing?
3.13	Is there anything you would find useful when deciding whether or not to allow your child's participation? (*Prompt:* what would be the top two/three issues that would make you positive about giving permission for your child to take part? And what would be the main two/three points that might make you want to say no?
3.14	Would you have given your permission for your child's to take part in the TOAST Trial? *(Prompt: Could you tell me a bit more about your reasons for this?)*
**Section 4- Outcome measures**	
As we have discussed, in the TOAST study we want to find out if routinely giving babies gastric acid suppression medication after repair of oesophageal atresia will reduce the incidence or severity of oesophageal stricture.<br><br>To do this we will collect information on Outcomes *(the list that I sent to you prior to interview).*<br><br>By collecting information on these main things, we hope to find out which should be used in the future. These are called outcome measures.	

Mitchell TK, *et al. BMJ Open* 2023; 13:e066070. doi: 10.1136/bmjopen-2022-066070

However, these outcomes have come from research papers and don't really give us much information on how children or families feel, or what is important to them. It is important that we include outcome measures that matter to children and their families.	
4.1	Thinking about your experience of your child's surgery and their health since that point in time, what would you hope the gastric acid suppression medication would do to help your child? (*Prompt: what effect would the medication have to be useful?*)
4.2	What would you be looking for as an indicator that the medication was helping your child?
4.3	What do you think about the outcome measures (*re-cap measures in the list provided*)<br>Is there another outcome measure that you think is important to families which we should be collecting information about in the TOAST Study?
4.4	*Recap on outcomes measured and ask them to put in order of importance* (e.g. So far you have mentioned x outcomes, X, Y & Z. which would you say is the most important for this study? Second most important for this study?)
**Section 5- Concluding comments**	
Finally, is there anything else you would like to say about this proposed trial?	
If we do find that it is feasible to do a trial, would you potentially be interested in being on a parent advisory group for the study? We would of course contact you and provide further information about what would be involved and would check this with you again as it may be some time in the future.	

Mitchell TK, *et al. BMJ Open* 2023; 13:e066070. doi: 10.1136/bmjopen-2022-066070
