# Supplementary material for: Mixed-methods feasibility study to inform a randomised controlled trial of proton pump inhibitors to reduce strictures following neonatal surgery for oesophageal atresia
Source: BMJ Open. 2023 Apr 20;13(4):e066070. doi: 10.1136/bmjopen-2022-066070 (PMC10124212; doi:10.1136/bmjopen-2022-066070)
Supplement: Supplementary data [file bmjopen-2022-066070supp005.pdf]

BMJ Publishing Group Limited (BMJ) disclaims all liability and responsibility arising from any re
placed on this supplemental material which has been supplied by the author(s)


# TOAST

Treating Oesophageal Atresia to Prevent Stricture

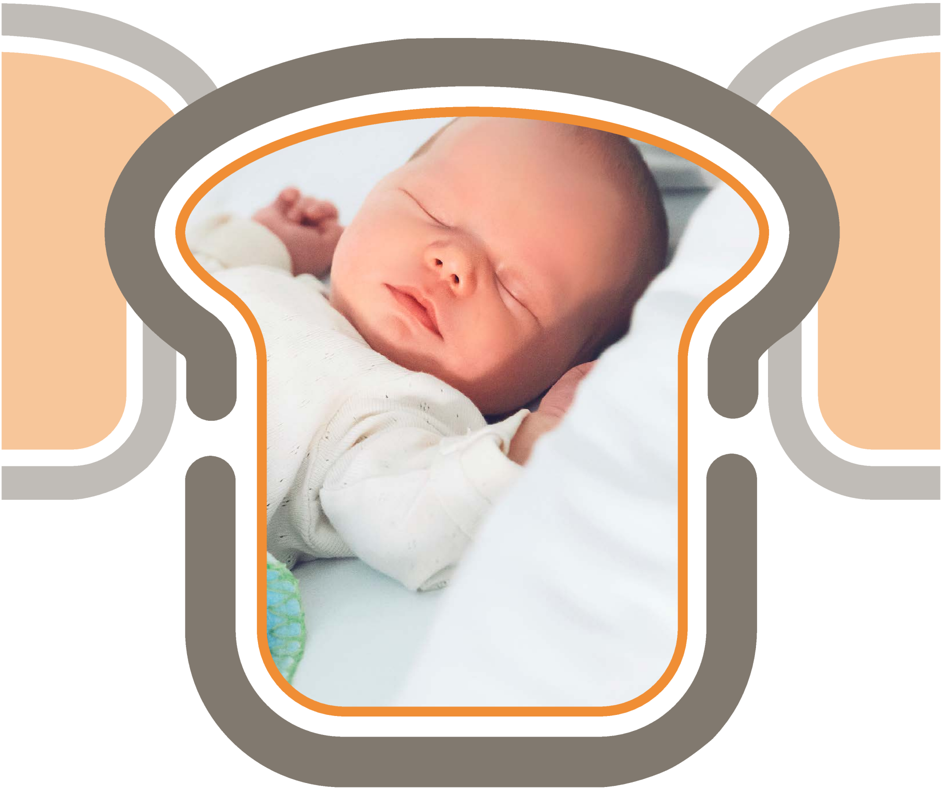

## Parent Information Leaflet

TOAST (PARENT) PIL REC ref: 8510 Version 0.4, 28-Jun-2021

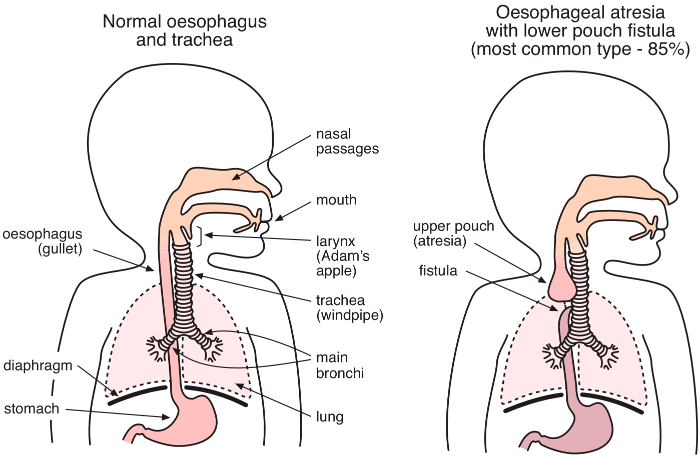


From Jaffray, B. (2016) 'Introduction to OA/TOF', in Martin, V. and Crabbe, D. (Eds.) The TOF Book. 2nd Edition. Nottingham, UK: TOFS., p. 17. ISBN 978-0-9536265-1-9. Copyright 2016 by TOFS. Reprinted with permission.

# TOAST

We understand that this is a difficult time for you and your family. We would like to invite you to take part in a study this hospital is taking part in for babies who have been born with oesophageal atresia. Before you decide it is important that you understand why the research is being done and what it will mean for you and your baby. Please ask us if there is anything else that is not clear or you would like to know.

## Why do we do research in healthcare?

Research is really important so that we can improve how we treat patients. If no research took place, then it would be difficult to improve outcomes for patients. All research in the NHS is voluntary.

## Why are we being asked to take part?

Your baby has been diagnosed with oesophageal atresia, where the oesophagus (swallowing tube) has not formed properly and so your baby's mouth is not connected to their stomach. The treatment for this condition involves surgery to reconstruct the oesophagus. The team looking after your baby will have talked to you about what this surgery involves.

We are asking you to help us with a research project to learn more about how treatment following this surgery can be made better. The study is called TOAST which stands for Treating Oesophageal Atresia to prevent STricture.

## Why are we doing this research?

One of the complications that can follow surgery to reconstruct the oesophagus is a narrowing called a stricture at the point where the repair is made. A stricture in the oesophagus would make it difficult for your baby to feed. Between 40% and 50% of babies get a stricture in the months after repair of oesophageal atresia. A stricture is usually treated by stretching the narrow section to make it wider in a procedure called a "dilatation". This requires an admission to hospital and is done under general anaesthetic. Some babies require several dilations during the first year of life.

Gastro-oesophageal reflux (where the stomach contents go back up into the



Mitchell TK, *et al. BMJ Open* 2023; 13:e066070. doi: 10.1136/bmjopen-2022-066070

oesophagus) is also very common after repair of oesophageal atresia and some surgeons think that reflux of acid into the oesophagus may make a stricture more likely or more severe. To try and prevent strictures from forming some surgeons use gastric acid suppression medication (e.g. omeprazole, 1ml/kg/day) in all babies, even if they have no symptoms of reflux, after repair of oesophageal atresia. The aim of using this medicine is to reduce stomach acid secretion so that even if there is reflux it is not as acidic.

Despite the common use of gastric acid suppression medication, we do not know for certain if there is any benefit to its use in babies following surgery. Indeed, some studies have indicated that babies routinely given gastric acid suppression medication may be more likely to get a stricture, but the evidence is not conclusive. There are other reasons why giving gastric acid suppression medication may not be a good idea, including that they may slightly increase the risk of infections and concerns about giving medicines to babies without proven benefit.

For these reasons, we are carrying out the TOAST study to find out if giving gastric acid suppression medication does help babies with oesophageal atresia. If it does, then all babies with oesophageal atresia can be given this treatment. If it does not, then we can stop asking parents to give medicine to their baby that has no benefit.

## What will happen if we take part?

The study we are asking you to give permission for your child to take part in is a randomised controlled trial (RCT). In the trial half of the babies will be given an gastric acid suppression medication from soon after their surgery to repair the oesophageal atresia and the other half will be given a cellulose/water solution as a ‘dummy’ medication called a placebo. We will use a computer to decide at random whether your baby receives gastric acid suppression medication or the placebo. The chance of your baby receiving either gastric acid suppression medication or placebo is equal. No one will know whether your baby is receiving the gastric acid suppression medication or the placebo. This includes you, the medical team and the research team. The reason a placebo is important is to make sure we are carrying out a fair test.

Your baby will be given the treatment each day by the medical team in hospital. When your baby is discharged home, we will ask you to give the treatment to your baby once daily for one year after their surgical repair. The doctors and nurses will show you how to do this. You will be asked to complete questionnaires about your baby’s progress, how much treatment you have given, hospital visits and how you are feeling. These will be completed through an app, online or



Mitchell TK, *et al*. *BMJ Open* 2023; 13:e066070. doi: 10.1136/bmjopen-2022-066070

on paper if you prefer. The doctors and nurses will provide you with further treatment supplies at your routine hospital visits and will be available to answer any questions you might have.

We will then follow your baby up for the first two years of their life to see if they develop a stricture and to monitor their progress. You will be asked to complete questionnaires at 3, 6, 9, 12, and 18 months, and 2 years about your baby's progress, how much treatment you have given, hospital visits and how you are feeling. These will be completed through an app, online or on paper if you prefer. Your baby will be followed up for the duration of their childhood whether they take part in this study or not and we will use these routine follow up visits to collect information relevant to the study so you will have the minimum inconvenience. If your baby does develop a stricture then they will need treatment and your doctors will explain this to you. At the end of the study in around 2028 we hope to have included about 211 babies born with oesophageal atresia. This is a number which we believe is big enough to give convincing results, and is far bigger than reported in any similar studies done worldwide to date.

We have tried to make taking part in the study as easy as possible for you and your family. The follow up has been designed around routine follow up visits that would happen for all babies born with oesophageal atresia so it should not mean extra visits to the hospital.

## What are the possible benefits or risks of taking part?

The treatments used in the study are used routinely for babies born with oesophageal atresia in UK hospitals and are known to be safe.

This study will not bring any immediate benefit to your child. We hope that we will get information about how best to treat children born with oesophageal atresia in the future. By participating, you will be helping us to learn whether treatment with gastric acid suppression medication is better than none so that we will know whether to offer this routinely as a treatment for other children in the future. After we have finished the study, we can let you know the results if you would like.

## Do we have to take part?

No, taking part is completely voluntary. If you decide not to take part, you do not have to give a reason and your child's care will not be affected in any way. If you agree to take part, we will ask you to sign a consent form which indicates your agreement to take part in the study and to let the researchers look at your child's health records. We will put a copy of this research consent form in your



Mitchell TK, *et al. BMJ Open* 2023; 13:e066070. doi: 10.1136/bmjopen-2022-066070

child's health records. We will give you a copy for your files.

## How long do I have to decide?

Because we would like to start treating your child soon after their operation to repair the oesophagus we will need to know if you are willing to take part within the next few days. To help you decide please talk about the study to your doctor. Please ask them any questions you may have.

## What if I change my mind?

You can change your mind about taking part at any time and remove your baby from the study without giving a reason. We would then only use the information we have collected up to the point when you withdraw. This would not affect the care your baby receives in any way.

## Will my details be kept confidential?

Your GP will be told that your baby is taking part in this study. We will collect personal information about you and your baby; the details will be kept securely and will only be seen by the research team, the study organisers in Oxford and people from the sponsor or regulatory authorities who check on studies such as this - this is a routine process of research in the NHS.

All information that we collect about you and your baby during the study will be kept strictly confidential and stored securely. We might ask for permission to contact you again in the future to find out how your baby is getting on as they grow up. If you decide to take part, we will collect some personal information about you and your baby, including name, NHS number, date of birth, address and contact details (e.g., email address and telephone number). This information will be sent to the Study Coordinating Centre at the University of Oxford, National Perinatal Epidemiology Unit Clinical Trials Unit (NPEU CTU). Authorised members of the research team will hold this data and store it securely for the purposes of running the trial. Authorised staff from the University of Oxford (as Coordinating Centre and Sponsor), funder, regulatory bodies, and your hospital may be given access to data for monitoring and/or audit of the study to ensure the research is complying with applicable regulations. Personal identifiable information including your telephone number and email address will be shared with Blue Frontier App provider for the purposes of recording the amount of medication given. The Study Coordinating Centre in Oxford will keep identifiable information about you and your baby from this study for 25 years after the study has finished. For more information on how we process and protect you and your baby's data, please see our website: ***https://www.npeu.ox.ac.uk/ctu/privacy-notice***



Mitchell TK, *et al. BMJ Open* 2023; 13:e066070. doi: 10.1136/bmjopen-2022-066070

BMJ Publishing Group Limited (BMJ) disclaims all liability and responsibility arising from any re
placed on this supplemental material which has been supplied by the author(s)


Further information can also be found at the NHS Health Research Authority's website:

***https://www.hra.nhs.uk/planning-and-improving-research/policies-standards-legislation/data-protection-and-information-governance/gdpr-guidance/templates/template-wording-for-generic-information-document/***

Please see our website for further information about how we protect your data: ***https://www.npeu.ox.ac.uk/ctu/privacy-notice***

## Who is organising and funding the study?

The study is funded by the National Institute of Health Research (NIHR) which is the research arm of the NHS. The study is sponsored by the University of Oxford and is being run by the National Perinatal Epidemiology Unit Clinical Trials Unit (NPEU CTU) at the University of Oxford. The researchers involved in the study are all experienced researchers, interested in improving the care given to babies born with oesophageal atresia. A number of parents have also helped design the study, as have TOFS, the UK support group for families affected by oesophageal atresia.

All research in the NHS is looked at by an independent group of people, called a Research Ethics Committee, to protect your interests and make sure it is done to the highest standards. This trial has been reviewed and given favourable opinion by a Research Ethics Committee.

## What will happen to the results of the research study?

At the end of the study, the results will be analysed and published in a medical journal. We will write our reports in a way that no one can work out who took part in the study. We will also share the results at medical and surgical conferences and with families of children with oesophageal atresia via the TOFS support group, who have been involved in setting up the trial. We will send you a copy of the results at the end of the study and we will also share them on our website. Unidentifiable data from this study may be shared with other groups who are carrying out similar work in the future.

## What happens if I have a complaint because of my baby taking part in this study?

In the first instance you can talk to the clinical team looking after your baby who will help you with your concern. You can also contact the local research team, either the research nurses or the principal investigator, their contact details are on the back page of this leaflet. If you wish to complain about any aspect of



Mitchell TK, *et al. BMJ Open* 2023; 13:e066070. doi: 10.1136/bmjopen-2022-066070

the way that you or your baby has been treated you may use the normal National Health Service complaints procedures, the Patient Advice and Liaison Service at your hospital will advise you about this (their contact details are also on the back page of this leaflet).

In the unlikely event your baby has been harmed by taking part in this study, you may have grounds for legal action and could seek compensation through the Research Sponsor, the University of Oxford, who has appropriate insurance-related arrangements in place. If your baby is harmed and it is due to any routine clinical treatment or negligence then the usual NHS indemnity arrangements will apply.

## Contact Information:

**Chief Investigators:**

Mr Nigel Hall, University of Southampton

Mr Iain Yardley, Evelina London Children's Hospital.

## Local Contact Details:

**Principal Investigator:**

{_LEAD_}

**Thank you for taking the time to read this information sheet**

## TOAST Study Team

NPEU Clinical Trials Unit,
National Perinatal Epidemiology Unit, University of Oxford,
Old Road Campus, OXFORD, OX3 7LF

01865 617919 toast@npeu.ox.ac.uk www.npeu.ox.ac.uk/toast

For more information about the TOFS support group please visit their website:
*www.tofs.org.uk/about-us.aspx*

TOAST is funded by the National Institute for Health Research (NIHR) Health Technology Assessment programme (project reference 131136).
The views expressed are those of the author(s) and not necessarily those of the NIHR or the Department of Health and Social Care.
