# Supplementary material for: Mixed-methods feasibility study to inform a randomised controlled trial of proton pump inhibitors to reduce strictures following neonatal surgery for oesophageal atresia
Source: BMJ Open. 2023 Apr 20;13(4):e066070. doi: 10.1136/bmjopen-2022-066070 (PMC10124212; doi:10.1136/bmjopen-2022-066070)
Supplement: Supplementary data [file bmjopen-2022-066070supp006.pdf]

TOAST

Evidence - and guideline - based rational decision tree for treating possible reflux symptoms in postoperative OA/TOF

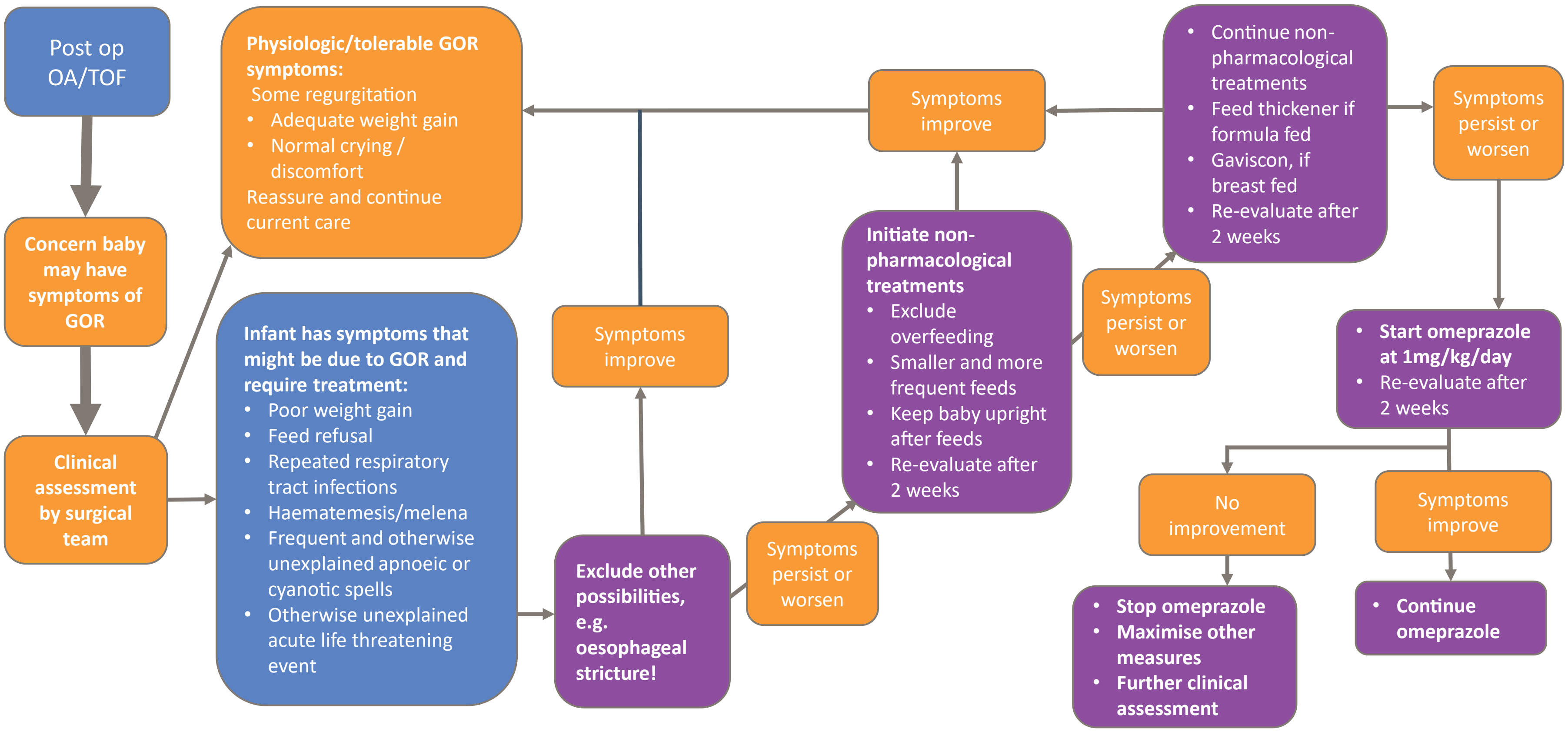

N.B. If you feel that urgent treatment is needed, clinical judgement takes precedence over the above.
