# Supplementary material for: Mixed-methods feasibility study to inform a randomised controlled trial of proton pump inhibitors to reduce strictures following neonatal surgery for oesophageal atresia
Source: BMJ Open. 2023 Apr 20;13(4):e066070. doi: 10.1136/bmjopen-2022-066070 (PMC10124212; doi:10.1136/bmjopen-2022-066070)
Supplement: Supplementary data [file bmjopen-2022-066070supp007.pdf]

TOAST

Treating Oesophageal Atresia to Prevent Stricture

Toast Trial: Outcomes

Severity of anastomotic stricture

- The number of dilations performed within one year of trial entry.

Incidence of anastomotic stricture

- Whether your baby has any dilatation or none in the first year / 2 years of their life.

Other complications directly related to oesophageal atresia

- Anything that is directly related to oesophageal atresia or its repair, for instance leakage from the join in the oesophagus.

Adverse events

- A general term used to describe things that don't go as planned but aren't included in other outcomes.

Presence of symptoms of gastro-oesophageal reflux

- Symptoms that may be due to reflux and reported by parents.

Need for treatment of reflux

- Whether your baby is given any sort of medication to treat reflux (e.g., feed thickener, Gaviscon).

Growth

- Your baby's growth during the first year of their life (recorded by weight, length and head circumference at certain time points).

Respiratory symptoms

- Presence of respiratory symptoms such as infections, chronic cough (but not the usual TOF cough) or any other symptoms that a doctor felt required investigation or treatment.

Oral Intake

- How your baby is progressing with advancing from milk feeds onto solids at specific ages.

Episodes of infection

- Any episode of proven infection (including respiratory infections).

Survival

- Whether your child survived to a certain time point (usually time point at months/years) or to a specific event (e.g., hospital discharge).

Maternal Health Related Quality of Life (EQ-5D-5L)

- A measure of a mother's quality of life using a specially designed questionnaire to understand how their baby's health may impact on their health and quality of life.

Child Health Related Quality of Life (PedsQL; Parent reported, collected at age 24 months)

- A measure of your baby's quality of life, as reported by a parent. This is measured using a specially designed questionnaire and is done at 2 years of age because this is the youngest age that it can reliably be done.

TOAST Study Team
NPEU Clinical Trials Unit, University of Oxford,
Old Road Campus, Headington, Oxford, OX3 7LF.
01865 617919 toast@npeu.ox.ac.uk
www.npeu.ox.ac.uk/toast

TOAST is funded by the National Institute for Health Research (NIHR) Health Technology Assessment programme (project reference 131136). The views expressed are those of the author(s) and not necessarily those of the NIHR or the Department of Health and Social Care.

TOAST Outcomes List v1.0, 28-Sep-2021

REC Reference: 8510
