# Supplementary material for: Mixed-methods feasibility study to inform a randomised controlled trial of proton pump inhibitors to reduce strictures following neonatal surgery for oesophageal atresia
Source: BMJ Open. 2023 Apr 20;13(4):e066070. doi: 10.1136/bmjopen-2022-066070 (PMC10124212; doi:10.1136/bmjopen-2022-066070)
Supplement: Supplementary data [file bmjopen-2022-066070supp008.pdf]

SUPPLEMENTARY FILE 8

a) Parent suggested edits to the symptomatic reflux treatment pathway

In the Blue box ***Infant has symptoms that might be due to GOR and require treatment*** six parents suggested the following be added:

- *'Clear visible pain'*/ '*clear discomfort'* (P4, mother)/ being really *'uncomfortable'* (P3; P5, mothers) or having a *'sore tummy'* (P9, mother) - *'arching their back'* (P3; P4; P6, mothers), *'pushing their tummy out'* (P5, mother) or *'squirming'* (P3; P5, mothers).
- *'Coughing'* (P12; P3, mothers) (not the usual TOF cough) and *'retching'* (P3, mother).
- *'Abnormal'* (P4, mother) or *'extreme'* (P3, mother) *'crying'* (P4; P5, mothers) or *'screaming'* (P4, mother).
- *'Not settling after feeds'* (P5, mother).
- *'Bringing up milk, even after holding them up for half an hour'* (P6, mother).
- *'Dry nappies'* (P9, mother).

In the purple *Exclude other possibilities, e.g., oesophageal stricture box*, add *'if they [GOR symptoms] are in combination with other usual stricture signs*' (P5, mother) (albeit, some parents did not receive much information about (P3, mother) or know what a stricture was at the beginning of their journey with a child with OA (P3; P9; P10, mothers). Some mothers did not know the signs of stricture (see below) to look out for (P5; P9, mothers). Furthermore, some parents were not told the word *stricture* or *dilatation* – terms such as *'narrowing'* or *'tightening'* (P12, mother) and *'stretch'* were used instead (P9, mother). Parents said that the other difficulty is that most signs of stricture are similar to those of reflux, such as:

- Coughing (P3; P5, mothers) or choking (P5; P15, mothers; P8, father), refusing (P5, mother), or not being able to swallow milk, food (P8, father; P12, mother) or saliva (P11, mother).
- Milk coming out of their nose (P5, mother).
- Not finishing milk feeds (P3, mother).
- Vomiting after feeds (P8; P17, fathers; P9; P13; P15, mothers).
- 'Blue events' when their child stopped/were struggling to breathe when trying to feed or lying down (P4; P5; P9; P12, mothers; P16; P18, fathers), or upset (P18, father):

Have a further box under *Start omeprazole - symptoms improve* for when children *'are better than [they were but are] still having a problem'* (P10, mother) and need the dosage increasing (P5, mother). Four mothers (P4; P5; P10; P12) felt that *'Omeprazole at 1mg per kilogram a day… [is] still quite a low dose of Omeprazole'* (P4, mother), with one child being *'started on 2mg per kilogram when he was in hospital'* (P12, mother) and another being on *'30mg per day … Why would they not pursue 'increase to maximum dose' and then 'symptoms improve, continue.' 'No improvement, stop Omeprazole.' Why stop the treatment before you've reached the maximum dose?'* (P4, mother).

Under ***Initiate Non-Pharmacological Treatments***, as well as holding the baby upright after feeds (P3; P5, mothers; P16, father), add:

- *'Keep stopping every five sucks'* if bottle fed to pace the feed (P3, mother).
- Remove *'animal milk protein from the mother's diet if the child is being breastfed, or from the formula, if the child is formula-fed'* (P4, mother).
- *'Incline the bed'* (P12, mother; P16, father)/*'Tip your cot at an angle'* (P4, mother) to ensure that the crib/cot/bed is higher at the head end (P10, mother). *'Things like propping them up and wedges … are standard things for a lot of TOFS children that might not get mentioned in a medical sense'* (P11, mother).

Mitchell TK, *et al. BMJ Open* 2023; 13:e066070. doi: 10.1136/bmjopen-2022-066070

• *'Any of the above [reflux] symptoms when parent has already initiated non-pharmacological treatment'* (P4, mother).
Explain acronyms such as *'GOR'* (P9; P10; P14, mothers; P18, father) or to have a *'key to terms'* (P12, mother) at the bottom of the diagram.<br>Explain medical terminology such as haematemesis/melena and unexplained apnoeic or cyanotic spells – perhaps word these as *'blue events'* (P12, mother), *'life-threatening events'* (P5, mother) or *'desaturations'* (P3, mother) because *'different hospitals … use slightly different terms'* (P12, mother) and some parents did not know what these were when worded this way (P3; P10, mothers), even though they learn *'pretty quickly'* (P10) what these are.
Consider accessibility:<br>*'Some people are colour blind and might find it hard to read on all the different colours', so it might be best to make the colours a darker shade to increase the contrast between the colours and the text'* (P13, mother).<br>*Left align all text (rather than some being 'centred')* (P13, mother).<br>Send the arrow from the first *Symptoms improve* box directly to the Physiologic/tolerable GOR symptoms box.

b) Clinician suggestions for edits to the symptomatic reflux treatment pathway

'I think the signs in the blue box need splitting into minor and minor. For example, poor weight gain or food refusal' (C20, surgeon, survey).
'Good flow chart - maybe mention in other conditions: consider tracheomalacia, as this would mimic reflux closely, but in some cases may require urgent surgical intervention' (C22, surgeon, survey).
'*I think it should be more explicitly pro breast feeding. Breastfeeding should come above artificial feeding in each list. Some may advocate investigation (e.g., pH study, contrast swallow etc) at some point. Where will that fall? Feeding in a more upright position as well as nursing after feeding in an upright position may help. In the normal physiology box, you could add something about regular bowels and wet nappies (to be reassuring)'* (C41, neonatologist, survey).
'Not clear in pathway when formal evaluation of GOR (pH/impedance)' (C46, surgeon, survey).
'In the pathway you have put exclude oesophageal stricture before excluding overfeeding and keeping the baby upright after feeds. I think this is the wrong way round because excluding stricture would involve endoscopy or contrast - not sure you would go to that step before you prop the baby up a little?' (C35, surgeon, survey). This quote supports that of one mother who said *'Obviously with the reflux and the GOR and things like that, they will go off and do the contrast studies… diagnosing whether reflux is an issue or not'* (P11, mother).
'What if a baby has gross clinical reflux / BRUEs consistent with severe reflux? Normally I would maximise PPI before taking the next steps? What if I perform a fundoplication in the first year of life?' (C4, surgeon, survey).
'What about anti-reflux surgeries? At what point would these be considered?' (C27, surgeon, survey).

Mitchell TK, *et al. BMJ Open* 2023; 13:e066070. doi: 10.1136/bmjopen-2022-066070
