# Supplementary material for: Mixed-methods feasibility study to inform a randomised controlled trial of proton pump inhibitors to reduce strictures following neonatal surgery for oesophageal atresia
Source: BMJ Open. 2023 Apr 20;13(4):e066070. doi: 10.1136/bmjopen-2022-066070 (PMC10124212; doi:10.1136/bmjopen-2022-066070)
Supplement: Supplementary data [file bmjopen-2022-066070supp009.pdf]

SUPPLEMENTARY FILE 9 Edits and additions for predefined outcomes

Predefined outcome	Suggested edits or additions
'Presence of symptoms of gastro-oesophageal reflux'	Collect data on tube feeding:<br>*[Child's name]'s reflux was a lot worst when he had the nasogastric tube and when the tube come out his instances of vomiting dramatically decreased. So, if children go home with a nasogastric tube, it might be interesting as part of the study to see the ones that had the antacid medicine and the ones that didn't to see the sort of effects of both* (P13, mother, interview).<br>Collect data on the *'number of [hospital] admissions related to GER'* (C25, surgeon, survey).
'Oral intake' and 'Growth'	Measure the age of solid food intake (P4; P15, mothers, interviews; P17, father, interview).<br>Collect data on *'the use or non-use of a gastrostomy'* (P17, father, interview), '*nasogastric tube'* (P13, mother, interview) or *'jejunal feeding without fundoplication'* (C47, surgeon, survey) because *'neonatologists and paediatricians will be… interested in feeding, nasogastric tube feeding and growth parameters'* (C41, neonatologist, focus group 2) / *'weight gain velocity'* (C21, surgeon, survey). '*That is the first question that comes into your mind when you are thinking about the quality of life for those children during the first and second year of their life'* (C41, neonatologist, focus group 2).
'Respiratory symptoms'	*'Add bronchiolitis'* (P15, mother, interview).<br>Measure *'any near-death attacks'* (C25, surgeon, survey) / *'ALTE'* [apparent life-threatening event/BRUE – brief, resolved, unexplained event] (C47, surgeon, survey).
'Episodes of infection'	*'Add bronchiolitis'* (P15, mother, interview) and aspiration (P5; P9, mothers, interviews; P18, father, interview):<br>*'You already have respiratory symptoms as an outcome but I wonder if evidence of aspiration would be better because it is really aspiration that we are worried about in the small infant post OA/TOF repair who may have reflux'* (C35, surgeon, survey).<br>Gastrointestinal infections:<br>*'G.I. infection including NEC later'* (C21, surgeon, survey).<br>*'Sepsis/G.I. infections. There is evidence of increased risk of sepsis in neonates who are given PPIs so I think it would be important to look at this specifically'* (C40, surgeon, survey).<br>*'Gastro [episodes of diarrhoea and vomiting] … because of the lack of acid it [PPI] gives bugs, which will be going into the gut first'* (C39, surgeon, focus group 2).
'Child health related quality of life'	Measure the time that children are hospitalised for (P11; P12, mothers, interviews), child's quality of sleep (P10; P11, mothers, interviews) and child's mental health:<br>'*His mental wellbeing is just as important as his physical wellbeing. In preventing strictures or reducing the severity of a stricture, ultimately, you're reducing hospital stays, you're reducing stress. He developed a fear of hospitals when he was about nine months old. As soon as he would see the entrance, he'd just be an absolute mess'* (P12, mother, interview).

Mitchell TK, *et al. BMJ Open* 2023; 13:e066070. doi: 10.1136/bmjopen-2022-066070

SUPPLEMENTARY FILE 9 Edits and additions for predefined outcomes

	Measure *'long term outcomes including Barrett's oesophagus'* (C11, surgeon, survey).<br>*'I think longer term follow-up would also be important as a secondary outcome at 5 and 10 years'* (C16, surgeon, survey).<br>Also see the edits or additions in 'Oral intake' and 'Growth' as these are overlapping.
'Maternal Health Related Quality of Life'	Amend to measure *'Parental'* health related quality of life (P12; P13, mothers, interviews):<br>*I don't think it should be limited to mothers, because obviously dads go through the same thing. I think it should be parental health related quality of life, rather than maternal. Actually, I work and my husband stays at home and looks after [Child's name] ... We don't have traditional gender roles at home. There are probably more families like that as well, so it's probably worth including dads, grandparents or whoever it is. That's probably the only thing I'd change is that it was parental rather than maternal* (P12, mother, interview).<br>*Maybe not just maternal, maybe it needs to be parental because it has an effect on certainly both parents* (P13, mother, interview).
'Other complications directly related to oesophageal atresia'	*'Other surgery – aortopexy / tracheopexy'* (C49, surgeon, survey).
'Severity of anastomotic stricture'	*'Anastomotic tension'* (C14, surgeon, survey).<br>Overlapping with 'Respiratory symptoms':<br>*'Any near-death attacks'* (C25, surgeon, survey).<br>*'ALTE'* [apparent life-threatening event/BRUE – brief, resolved, unexplained event] (C47, surgeon, survey).
'Need for treatment of reflux'	*'Fundoplication'* (C47, surgeon, survey)<br>*'Other surgery – fundoplication'* (C49, surgeon, survey).
