# Supplementary material for: Mixed-methods feasibility study to inform a randomised controlled trial of proton pump inhibitors to reduce strictures following neonatal surgery for oesophageal atresia
Source: BMJ Open. 2023 Apr 20;13(4):e066070. doi: 10.1136/bmjopen-2022-066070 (PMC10124212; doi:10.1136/bmjopen-2022-066070)
Supplement: Author's manuscript [file bmjopen-2022-066070.draft_revisions.pdf]

BMJ Open

BMJ Open is committed to open peer review. As part of this commitment we make the peer review history of every article we publish publicly available.

When an article is published we post the peer reviewers' comments and the authors' responses online. We also post the versions of the paper that were used during peer review. These are the versions that the peer review comments apply to.

The versions of the paper that follow are the versions that were submitted during the peer review process. They are not the versions of record or the final published versions. They should not be cited or distributed as the published version of this manuscript.

BMJ Open is an open access journal and the full, final, typeset and author-corrected version of record of the manuscript is available on our site with no access controls, subscription charges or pay-per-view fees (http://bmjopen.bmj.com).

If you have any questions on BMJ Open's open peer review process please email info.bmjopen@bmj.com

BMJ Open

A mixed methods feasibility study to inform a randomised controlled trial of proton pump inhibitors to reduce strictures following neonatal surgery for oesophageal atresia

Journal:	*BMJ Open*
Manuscript ID	bmjopen-2022-066070
Article Type:	Original research
Date Submitted by the Author:	04-Jul-2022

Complete List of Authors:	Mitchell, Tracy; University of Liverpool, Public Health, Policy and Systems<br>Hall, Nigel; University of Southampton Faculty of Medicine, University Surgery Unit<br>Yardley, Iain; Evelina Children's Hospital, Guy's & St. Thomas's NHS Foundation Trust,<br>Cole, Christina; University of Oxford, National Perinatal Epidemiology Unit, Clinical Trials Unit, Nuffield Department of Population Health<br>Hardy, Pollyanna; University of Oxford, Clinical Trials<br>King, Andy; University of Oxford,<br>Murray, David; University of Oxford, National Perinatal Epidemiology Unit, Clinical Trials Unit, Nuffield Department of Population Health<br>Nuthall, Elizabeth; University of Oxford, National Perinatal Epidemiology Unit, Clinical Trials Unit, Nuffield Department of Population Health<br>Roehr, Charles; University of Oxford, National Perinatal Epidemiology Unit, Clinical Trials Unit, Nuffield Department of Population Health<br>Stanbury, Kayleigh; National Perinatal Epidemiology Unit, Clinical Trials Unit, Nuffield Department of Population Health, University of Oxford, Old Road Campus<br>Williams, Rachel; University of Oxford, National Perinatal Epidemiology Unit, Clinical Trials Unit, Nuffield Department of Population Health<br>Pearce, John; TOFS<br>Woolfall, Kerry; University of Liverpool, Institute of Population, Health and Society

Keywords:	QUALITATIVE RESEARCH, Paediatric gastroenterology < PAEDIATRICS, STATISTICS & RESEARCH METHODS, NEONATOLOGY, Neonatal intensive & critical care < INTENSIVE & CRITICAL CARE

SCHOLARONE™
Manuscripts

I, the Submitting Author has the right to grant and does grant on behalf of all authors of the Work (as defined in the below author licence), an exclusive licence and/or a non-exclusive licence for contributions from authors who are: i) UK Crown employees; ii) where BMJ has agreed a CC-BY licence shall apply, and/or iii) in accordance with the terms applicable for US Federal Government officers or employees acting as part of their official duties; on a worldwide, perpetual, irrevocable, royalty-free basis to BMJ Publishing Group Ltd ("BMJ") its licensees and where the relevant Journal is co-owned by BMJ to the co-owners of the Journal, to publish the Work in this journal and any other BMJ products and to exploit all rights, as set out in our licence.

The Submitting Author accepts and understands that any supply made under these terms is made by BMJ to the Submitting Author unless you are acting as an employee on behalf of your employer or a postgraduate student of an affiliated institution which is paying any applicable article publishing charge ("APC") for Open Access articles. Where the Submitting Author wishes to make the Work available on an Open Access basis (and intends to pay the relevant APC), the terms of reuse of such Open Access shall be governed by a Creative Commons licence – details of these licences and which Creative Commons licence will apply to this Work are set out in our licence referred to above.

Other than as permitted in any relevant BMJ Author's Self Archiving Policies, I confirm this Work has not been accepted for publication elsewhere, is not being considered for publication elsewhere and does not duplicate material already published. I confirm all authors consent to publication of this Work and authorise the granting of this licence.

For peer review only

A mixed methods feasibility study to inform a randomised controlled trial of proton pump inhibitors to reduce strictures following neonatal surgery for oesophageal atresia

Tracy K Mitchell [a], Nigel J. Hall [b], Iain Yardley [c], Christina Cole [d], Pollyanna Hardy [d], Andy King [d], David Murray [d], Elizabeth Nuthall [d], Charles C. Roehr [d], Kayleigh Stanbury [d], Rachel Williams [d], John Pearce [e], Kerry Woolfall [a]

Academic affiliations

[a] Institute of Population Health, Department of Public Health, Policy and Systems, University of Liverpool, UK.
[b] University Surgery Unit, Faculty of Medicine, University of Southampton, UK
[c] Evelina Children's Hospital, London, UK; Faculty of Life Sciences and Medicine, King's College London, UK
[d] National Perinatal Epidemiology Unit, Nuffield Department of Population Health, University of Oxford, UK
[e] TOFS, Nottingham, UK

Email addresses

tracy.mitchell@liverpool.ac.uk
n.j.hall@soton.ac.uk
Iain.Yardley@gstt.nhs.uk
christina.cole@npeu.ox.ac.uk
pollyanna.hardy@npeu.ox.ac.uk
andy.king@npeu.ox.ac.uk
david.murray@npeu.ox.ac.uk
elizabeth.nuthall@npeu.ox.ac.uk
charles.roehr@npeu.ox.ac.uk
kayleigh.stanbury@npeu.ox.ac.uk
rachel.williams@npeu.ox.ac.uk
johnpearcetofs@googlemail.com
woolfall@liverpool.ac.uk

ORCID IDs

Tracy Karen Mitchell 0000-0003-0014-8016
Nigel J. Hall 0000-0001-8570-9374
Iain Yardley 0000-0002-6928-2267
Christina Cole 0000-0002-8798-2136
Pollyanna Hardy 0000-0003-2937-8368
Andrew R. King 0000-0001-5180-7179
David Murray 0000-0001-9010-2905
Elizabeth Nuthall 0000-0002-5092-7643
Charles C. Roehr 0000-0001-7965-4637
Kayleigh Stanbury 0000-0002-8726-2411
Rachel Williams 0000-0002-5872-1690
Kerry Woolfall 0000-0002-5726-5304

For peer review only

Corresponding author

Tracy Mitchell, Public Health, Policy, and Systems, Institute of Population Health, University of Liverpool, Room 213, Second Floor, Whelan Building, Quadrangle, Liverpool L69 3GB, UK; tracy.mitchell@liverpool.ac.uk Twitter @trakmitch

Key words

Qualitative research, Paediatric gastroenterology, Paediatric surgery, Statistics and research methods, Neonatology, Neonatal intensive and critical care

Abstract

Objectives: This mixed methods feasibility study aimed to explore parents' and medical practitioners' views on the acceptability and design of a clinical trial to determine whether routine prophylactic proton pump inhibitors (PPI) reduce the incidence of anastomotic stricture in infants with oesophageal atresia (OA).

Design: Semi-structured interviews with UK parents of an infant with OA and an online survey, telephone interviews and focus groups with clinicians. Data were analysed using reflexive thematic analysis and descriptive statistics.

Participants We interviewed 18 parents of infants with OA. Fifty-one clinicians (49 surgeons, 2 neonatologists) from 20/25 (80%) units involved in OA repair completed an online survey and 10 took part in one of two focus groups. Interviews were conducted with 2 clinicians whose survey responses indicated they had concerns about the trial.

Results: All parents and most clinicians found the use, dose and duration of omeprazole as the intervention medication, and the placebo control, as acceptable. Parents stated they would hypothetically consent to their child's participation in the trial. Concerns of a few parents and clinicians about infants suffering with symptomatic reflux, and the impact of this for study retention, appeared to be alleviated through the symptomatic reflux treatment pathway. Hesitant clinician views appeared to change through discussion of parental support for the study and by highlighting existing research that questions current practice of PPI treatment. Parents and clinicians ranked the same top four outcomes as important to measure for this study.

Conclusions Our findings indicate that parents and most clinicians view the proposed TOAST trial to be feasible and acceptable so long as infants can be given PPI if clinicians deem it clinically necessary. This insight into parent and clinician views and concerns will inform pilot phase trial monitoring, staff training and the development of the trial protocol.

Article summary

Strengths and limitations of this study

- A mixed methods approach including a survey, interviews and focus groups enabled comprehensive insight into key stakeholder views.
- Despite the difficulties experienced in arranging interviews we continued to interview parents until the point of information power (1) and to involve parents of infants with OA at all stages, including study design and conduct, as members of the study team.

For peer review only - http://bmjopen.bmj.com/site/about/guidelines.xhtml

- Our sample may comprise of experienced parents with an interest in OA research and may not reflect the potential TOAST sample who will also have less awareness of PPI and treatment options for symptoms of reflux at the time the trial is discussed.
- Our study includes the perspectives of clinicians involved in the treatment of OA representing the majority of UK surgical units.

Introduction

Oesophageal atresia (OA) is a rare congenital anomaly that affects a baby's oesophagus, where the upper part of the oesophagus does not connect with the lower part. As this is life threatening, surgery is usually carried out shortly after birth. Approximately 150 babies are born with OA annually in the United Kingdom (UK) (2). Stricture (narrowing) at the anastomosis (new connection) is the most common post-operative complication in the months following surgical repair (2,3), which requires admission to hospital for investigation and dilatation of the narrowed segment under general anaesthesia (3).

Some international guidelines (4) recommend routine proton pump inhibitors (PPI) for all infants with OA for the first year of life to reduce the incidence of anastomotic stricture. Some studies (2,5,6) and a systematic review and meta-analysis of the evidence (7), however, indicate that infants routinely given PPI are no less likely to get a stricture. The evidence to support the use of PPI is not conclusive and stems from only a small number of low quality, observational or single centre studies (7). Furthermore, PPI can increase the risk of gastrointestinal (2,6,8,9) and respiratory infections (4,8), raising concerns about giving medication to infants that has no benefit.

A randomised clinical trial is needed to answer the question 'In infants born with oesophageal atresia, does the routine use of PPI compared to matched placebo impact the incidence or severity of anastomotic stricture?' The chances of successful trial completion are improved if the trial is deemed to be acceptable to parents and clinicians. This paper presents the findings of a mixed methods feasibility study which aimed to explore parent and practitioner views on the feasibility, acceptability and design of a proposed randomised controlled trial: Treating Oesophageal Atresia with prophylactic proton pump inhibitors to prevent STricture (TOAST).

Methods

Study Design

We conducted a mixed methods study involving interviews with parents of an infant born with OA in the last three years (June – September 2021), as well as an online survey (August – October 2021), interviews (October 2021) and focus groups (November – December 2021) with clinicians caring for infants with OA.

We used previous research (10,11) to develop participant information sheets (PIS) (see **online supplementary file 1**), protocol and online survey (see **online supplementary file 2**), whilst ongoing findings were used to develop topic guides and as part of an iterative process. Topic guides and the survey included questions on the proposed trial design, information materials, trial acceptability, willingness to be involved/provide consent, the approach to consent and parent prioritised outcomes for the proposed trial. The research was conducted in the UK between June and December 2021. The consolidated criteria for reporting qualitative research (COREQ) checklist (12) was used to aid reporting (see **online supplementary file 3**).

For peer review only - http://bmjopen.bmj.com/site/about/guidelines.xhtml

Patient and Public Involvement

Our parent advisory group (PAG) involved members of TOFS charity, who support infants born with OA/TOF (Tracheo-Oesophageal Fistula). The PAG met regularly before and during the study, providing valuable input into the design of research materials (including topic guide and draft reflux treatment pathway) and the conduct, progress and findings of this study. JP (TOFS Trustee) was a member of the TOAST study management team and a TOFS representative for all aspects of study development and conduct.

Recruitment and sampling procedure

Based on previous feasibility studies (10,11,13) we anticipated that we would need to interview 15-25 parents to reach information power (1), which is the point at which data addresses the study aims; sample specificity (e.g., participants' experience relevant to the study aims and sample diversity) (12); our reflexive and interpretive approach to theory and analysis (14,15); and sufficient quality of interview dialogue (1). Parents were recruited via direct email from our collaborating support group TOFS, as well as via social media and study website advertising.

We aimed to recruit at least 50 clinicians to the online survey from approximately 18/25 (75%) of UK units. IY (male, Paediatric Surgeon) distributed an invitation to participate in the survey through the UK Children's Upper Gastrointestinal Surgery (CHUGS) network with a request to cascade the survey link to clinicians involved in the care of OA infants. We aimed to purposively sample clinicians who raised concerns about the proposed trial design in their survey responses and invite them to participate in a telephone interview to further explore their concerns and discuss potential ways these could be addressed to assist 'buy in'. Finally, we invited survey participants to attend an online or face-to-face focus group.

Eligibility screening and conduct

TM (female, Research Methodologist) responded to parents' email and social media responses in sequential order, confirmed eligibility and emailed them a proposed trial Parent Information Leaflet (PIL) (see **online supplementary file 4**), draft treatment pathway for symptomatic reflux (see **online supplementary file 5**), and potential list of outcome measures (see **online supplementary file 6**) derived from a review of the literature. KW (female, Social Scientist) contacted clinicians to arrange interviews and IY sent invitations to attend a focus group.TM and KW facilitated interviews and focus groups. Respondent validation was used to add unanticipated topics to the topic guide as interviewing and analysis progressed (16). Findings from parent interviews and online survey were used to develop the topic guide for the clinician interviews and focus group. Interviews stopped when information power (1) was achieved and all clinicians who responded to the invite were interviewed. Parents were sent a £30 shopping voucher after their interview to thank them for their time.

Analysis

TM led the analysis with oversight from KW. Analysis of direct questioning and indirect discussion was broadly interpretive and inductive, informed by the Theoretical Framework of Acceptability (TFA), and adapted version for paediatrics (10,17). NVivo V.12 software (18) was used to assist the organisation and coding of data. TM and KW met regularly to discuss interpretation and develop the coding framework. Outcome measures prioritised as being most important were given a score of three, second most important a score of two, and third

For peer review only - http://bmjopen.bmj.com/site/about/guidelines.xhtml

most important a score of one. Outcomes were then ranked. Quantitative data were entered into Microsoft Excel (19). Descriptive statistics are presented with frequencies and percentages. Synthesis of qualitative and quantitative data for mapping findings to the TFA drew on the constant comparative method (20,21).

Results

Sample

A total of 39 parents registered interest and were screened. Three parents were deemed ineligible, three booked interviews but cancelled due to their child's hospital readmission and 15 parents did not respond to initial contact. Information power was reached at 18 parent interviews (representing 17 children), which took place via telephone (n=15) or online (n=3), lasting between 40 and 92.5 minutes, median 65 minutes (see **Table 1** for parent and child characteristics). Nine parents were recruited through TOFS, three from social media (Facebook) and six could not recollect whether TOFS email or Facebook.

Table 1 Parent and child characteristics

Parent	Mother (n=13)<br>Father (n=5)
Parent age	Between 29 and 42 years (mean = 36 years; median = 36 years)
Child age	Between 4 weeks and 34 months (mean = 14.1 months old; median = 17 months)
Gestation	Term (n=14)<br>Premature (n=3) 31^{+0} weeks, 33^{+0} weeks and 33^{+3} weeks
Country of residence	England (n=15)<br>Scotland (n=3)
Ethnic group	White British (n=15)<br>White Scottish (n=1)<br>White Other (n=1)<br>Indian (n=1)

Fifty-one clinicians (49 paediatric surgeons; 2 neonatologists) from 20/25 (80%) sites completed the online survey. Four of the six clinicians (paediatric surgeons) who indicated in the survey that they did not find the trial acceptable had provided their contact details and were contacted to take part in an interview. Two did not respond to contact and two surgeons from different sites took part in an online Zoom interview (lasting 23 and 27 minutes). Ten clinicians from nine different sites (36%) took part in one of two focus groups, one face-to-face (n=5 surgeons), one online via Zoom (n=4 surgeons; n=1 neonatologist). Both focus groups lasted one hour. See **Table 2** for clinician characteristics.

Table 2 Clinician characteristics

Method of data generation	No. of clinicians and role	No. of sites represented
Online survey	n=51: 49 paediatric surgeons and 2 neonatologists	20 (80%)
Interview	n=2: consultant paediatric surgeons	2 (8%)
Focus group	n=10<br>Focus group 1: 5 consultant paediatric surgeons.<br>Focus group 2: 2 consultant paediatric surgeons, 2 paediatric surgeons and 1 consultant neonatologist.	9 (36%)

For peer review only - http://bmjopen.bmj.com/site/about/guidelines.xhtml

Trial research question

Across the research methods, the majority of parents and clinicians (through survey responses) described or indicated that the proposed trial would answer an important research question and help address '*how little evidence there is'* (P10, mother, interview). Some parents spoke of their hope that the study would help future babies with OA, whilst both parents and clinicians stated the trial was needed to help standardise practice, and prevent babies from taking potentially unnecessary medication, whilst also reducing costs and burden for families and the NHS.

> *'I think it [the proposed trial] is a good thing, because I hear a lot of parents on the TOFS site and things, and they are obviously getting different medical care and their concerns about that really. I think it is something that needs to be standardised'* (P11, mother, interview).

> *'If significant difference found then potential to decrease burden on families and providers'* (C44, surgeon, survey).

> *'If your child doesn't need to be on a medication, then you don't really want them to be on it'* (P13, mother, interview).

Parent information

Interviews and focus groups involved a review of a draft trial Participant Information Leaflet (PIL). The majority of parents said that they found the proposed PIL to be clear and understandable. However, some stated it was *'quite long'* (P10, mother, interview) and '*text heavy'* (P16, father, interview), whilst acknowledging all necessary information was included. Recommended changes included adding a one-page overview of the study; highlighting the differences in treatment that are already happening; and not using acronyms.

Symptomatic reflux treatment pathway

The study team recognised the need to develop a tool to assist clinicians in making decisions about how to treat babies who had symptoms of reflux during the proposed trial. A symptomatic reflux treatment pathway was developed, which included options for non-pharmacological treatments (e.g., exclude overfeeding, keep baby upright after feeds) and time frames for re-evaluation (e.g., every two weeks). During interviews and focus groups the draft symptomatic reflux treatment pathway was described as *'helpful for parents and clinicians*' (C51, surgeon, survey). Parents' suggestions for improvement were mainly around the additional symptoms of reflux, signs of stricture, other non-pharmacological treatments that could be initiated, and accessibility of the document (see **online supplementary file 7, Table 1**). Clinicians suggested adding conditions such as tracheomalacia (C22, surgeon, survey), the timing of/whether babies have *'anti-reflux'* surgery (C27, surgeon, survey), and prioritising breastfeeding over formula feeding (C41, neonatologist, survey) (see **online supplementary file 7, Table 2**).

Although the treatment pathway had been originally designed for use by clinicians during the trial, parents highlighted how it would be helpful to refer to and *'be aware of the things that are written down… just as a reminder of, for example, it says, "Are they gaining weight adequately? … Is he crying normally?"'* (P7, mother, interview). One father suggested that the

For peer review only - http://bmjopen.bmj.com/site/about/guidelines.xhtml

pathway will *'make them [parents in the trial] feel more comfortable'* (P8, interview) about trial participation. Parents said they would be happy to follow the symptomatic reflux treatment pathway, *'so long as no child is being left to suffer'* (P13, mother, interview) and '*the health of the individual child would trump… being in the study*' (P18, father, interview).

Most clinicians (n=38/51, 74.5%) indicated in the survey that they would be happy to follow the treatment pathway (see **online supplementary file 5**); others raised concerns about the potential 4-week timeframe to PPI (n=5); the severity of reflux symptoms (n=4); and retention of participants (n=3):

> *'Slight concern that it may be difficult to get TOFOA parents to agree to … wait a further 4 weeks … if their child is symptomatic'* (C49, surgeon, survey)
>
> '*This would be fine for minor symptoms but inappropriate for severe symptoms'* (C12, surgeon, survey)
>
> *'I think if there is a clinician who wants to take someone out of it [the trial], you could use that escalation policy [symptomatic reflux treatment pathway] to do so...That's the difficult thing, I think'* (C29, surgeon, interview).

Support for Omeprazole as the intervention, but some concerns about side effects

Most clinicians (including 60.8% of survey participants) routinely administered or prescribed PPI following surgery in all babies with type C OA under their care. During interviews and focus groups some clinicians stated that they did not prescribe PPI following surgery due to a lack of evidence about stricture formation, or when patients did not have any symptoms of reflux. Side effects of PPI, such as the increased risk of infections, not knowing the long-term risks, or wanting to minimise unnecessary drug use were also reasons not to use PPI.

Nevertheless, all parents and the vast majority of clinicians, found Omeprazole acceptable as the trial intervention as it was a *'routinely used by many teams with a very safe profile'* (C35, surgeon, survey). The dose of 1mg/kg omeprazole orally once daily for one year was also described as being acceptable, although four mothers and two clinicians perceived 1mg/kg Omeprazole per day to be a low dose, and had *'slight concern that it may be difficult to get parents [of children with OA] to agree to that dose'* (C49, surgeon, survey). Some parents, however, wondered whether PPI *'actually had any effect'* (P17, father, interview) because their child *'still had a stricture'* (P12, mother, interview) even though they had taken PPI from birth, whilst one father (P1, interview) and one surgeon (C21, focus group 1) said that children were left off PPI and *'nothing happened anyway'* (C21, surgeon, focus group 1). A minority of parents and clinicians had concerns about side effects, such as *'it [omeprazole] seemed to thicken her mucus a lot, so it produced more blue episodes'* (P16, father, interview), *'a very sore tummy'* (P9, mother, interview) and *'sepsis/G.I. infections'* (C40, surgeon, survey), and were concerned about the trial length due to the long-term impacts of the medication. One surgeon said that infants should take PPI for '*6 months only to avoid side effects*' (C26, survey).

For peer review only - http://bmjopen.bmj.com/site/about/guidelines.xhtml

Treating reflux in the comparator arm and the challenge of changing practice

The use of a placebo in the comparator arm of the proposed trial was acceptable to both groups, although parents stated that babies should not be *'left to suffer'* with reflux (P13, mother, interview) and the symptomatic reflux treatment pathway should *'be implemented sensibly'* (P4, mother, interview), however, clinicians should not *'automatically assume you need'* PPI (P9, mother, interview). A minority of clinicians were concerned about a change in practice and placing babies at risk of negative outcomes if they were not given PPI in the trial, particularly if they have symptomatic reflux and tight anastomosis:

> *'Babies in the placebo group are exposed to a high risk of complications… It is not safe to have a baby post-TOF without PPI'* (C9, surgeon, survey).

> *'I would struggle to join a clinical trial where I know that there is a randomisation of my symptoms who were not using PPIs… I was taught the importance of the PPIs [during my career] and I think it make sense to use PPIs in this condition [OA]…if you do any repair of a tissue, you don't want to spill acid on it'* (C9, surgeon, interview).

Inclusion and Exclusion Criteria

Most clinicians who took part in the survey and focus groups were satisfied with the proposed inclusion and exclusion criteria (see **Box 1**). Recommendations for improvement covered five key areas: 1) Include babies who require *'delayed'* or *'staged'* repairs (n=11) '*as PPI might be beneficial in those'* (C43, surgeon, survey); 2) Exclude babies with tight anastomosis because of the perceived increased risk of *'reflux'* (C23, surgeon, survey) and *'stricture formation'* (C3, surgeon, survey) (n=7); 3) Exclude pre-term babies - *'Use of PPI in preterm is not neutral, and has been shown to be associated with NEC [necrotising enterocolitis] and fungal in sepsis'* (C33, neonatologist, survey) (n=5); 4) Exclude babies with other anomalies (n=9) such as '*co-existent duodenal atresia or ARM'* (C45, surgeon, survey), *'cardiac / renal / neurological / chromosomal'* (C7, surgeon, survey), '*congenital oesophageal stenosis (COS)*' (C27, surgeon, survey), '*HIE/ major brain injury* …and *VACTERL* [vertebral defects, anal atresia, cardiac defects, tracheo-oesophageal fistula, renal anomalies, and limb abnormalities]*'* (C4, surgeon, survey); and, 5) Include but consider *'the homogeneity of the… population'* of (C27, surgeon, survey) babies who have thoracoscopic rather than open repairs (n=2).

Box 1 Proposed inclusion and exclusion criteria

Inclusion criteria	Exclusion criteria
• Infants with OA with distal tracheo-oesophageal fistula undergoing primary repair at the first operative intervention in the newborn period<br>• Written informed parental consent	• Infants undergoing staged repair or delayed primary repair or requiring emergency ligation of tracheo-oesophageal fistula with primary repair later<br>• No realistic prospect of survival

The importance of not discussing the trial on the day of surgery

Parents were then asked to consider when would be the most acceptable time to be approached about the proposed trial. Most stated that two to three days after birth would be best, as long as they have received *'the good news of [their baby having] a successful repair'* (P18, father, interview) and when their baby is *'starting to look stable'* (P4, mother, interview) and is off the ventilator. A clear message from parents was that trial discussions on the day of surgery would be too overwhelming. Some suggested the trial could be discussed with parents

For peer review only - http://bmjopen.bmj.com/site/about/guidelines.xhtml

prior to birth if OA is diagnosed antenatally. Clinicians made similar recommendations to broach the discussion within 72 hours post-surgery. The concerns of the three clinicians who did not find it acceptable to approach parents at this time were, once again, around the safety of infants with OA who do not receive PPI:

> *'I usually start PPI from the time of surgery. It is not acceptable to leave the baby without PPI for 72 hours. The baby should be randomised before the surgery'* (C9, surgeon, survey).

The use of a mobile application to assist trial retention

Parents' views were sought on the use of a mobile phone application (app), which would include reminders to administer the trial intervention when they had left hospital. Most parents thought that the app was a good idea and would be a *'massive bonus'* (P7, mother, interview) and so *'useful'* (P13, mother, interview) *'to offer with'* the trial (P18, father, interview). Seven parents felt that the app is not needed, although were not averse to having an app for the study, so long as it would not be a mandatory requirement for parents to use it.

Furthermore, when questioned about content that might be useful in an app, most made a number of suggestions such as: *'hints'* (P12, mother, interview), *'tips'* (P6, mother, interview) *'and advice … on how to [prepare and] administer'* (P13, mother, interview) the intervention; reminder notifications; symptoms tracker and a medical history page because *'the days all merge… [and] sometimes I'll be like, "Oh yes, he's been coughing." And then the surgeon will be like, "So how long has that been going on for?" And I'm like, "Oh Gosh, I don't know"'* (P3, mother, interview). Other suggestions included information about the study and the main signs of reflux and stricture and *'a guide to CPR because I know a lot of parents are very, very anxious about that'* (P4, mother, interview).

Shared views on outcomes of importance

Parents and clinicians were then asked to consider a list of potential outcomes sent prior to interview and focus groups, as well as any additional outcomes they felt should be included. Parents and clinicians suggested edits or additions to most of the predefined outcomes, as shown in **online supplementary file 8**.

Participants were asked to rank the outcomes that were most, second and third most important to be measured in the TOAST trial. After weighting, 'Severity of anastomotic stricture', 'Incidence of anastomotic stricture', 'Need for treatment of reflux' and the 'Presence of symptoms of reflux' remained the four most important outcome measures for the TOAST trial for both parents and clinicians.

Potential barriers to trial success

In the survey, 38 (38/51; 74.5%) clinicians stated they were a little (n=32) or very (n=6) concerned about the retention of babies in the trial if they have symptomatic reflux and were receiving the placebo, reflecting the concerns of some parents and clinicians interviewed:

> *'You might put a baby in a placebo group, but if they have reflux, they'll have to have the antacid medication, and reflux is really- well, as far as I'm aware, really common'* (P3, mother, interview).

For peer review only - http://bmjopen.bmj.com/site/about/guidelines.xhtml

> *'If babies become symptomatic then parents may ask to come out of trial and be assured that they're on a PPI'* (C20, surgeon, survey).

Some clinicians' concerns about retention of participants in the trial related to stricture management in babies with signs of reflux:

> *'If a patient has a particularly difficult stricture, and signs of reflux I would want to know if they are being treated or just on placebo, as at this point, I would definitely want them on a PPI'* (C15, surgeon, survey).

Other clinicians' concerns were about geographical challenges, highlighting the need for '*as many continuity sites as possible'* (C33, surgeon, survey) to be tertiary centres and *how 'some of our remote / poorer patients would struggle to travel to face-to-face follow-up'* (C4, surgeon, survey). A combination of external factors and trial setup queries were discussed including: the *'differing views of… surgical and neonatal (and other) colleagues'* (C21, surgeon, survey) about preference for use of PPI and prescribing outside of the trial; quality of intervention blinding and sourcing; staffing and research support issues, especially *'out of hours'* (C2; C30, surgeons, survey); access to training and support needs; and, reflecting the concerns of a small number of parents, the pro-medication influence of TOFS Facebook group members:

> *'It will be interesting to see what the parents have said, and what, like the TOFS group says, because I think most of the parents will be members of that group, and what they feel about reflux and how willing they would be if they go on the forum and say, "Oh, I think my kid's refluxing and he's on this trial. What should I do?" What advice they're going to be given from the parent groups because I think that would be a big factor'* (C29, surgeon, interview).

> *'It was only when I joined the TOFS Facebook group that I thought, "Oh dear there's a lot of stuff going on and a lot of complications and a lot of people talking about medication the whole time"'* (P9, mother, interview).

Overall views on trial acceptability

Towards the end of the interview, survey or focus group participants were asked to consider the overall acceptability of the trial. All eighteen parents stated that the proposed trial was acceptable; three with the proviso that their child could access PPI medication if clinically necessary and so long as *'a nice, softly-softly approach'* (P16, father, interview) was taken by an experienced and *'trusted doctor or surgeon'* (P9, mother, interview). Having trust in the opinions of health professionals about their child's involvement in the trial was mentioned (unprompted) by over half of parents.

Almost all clinicians stated that they found the proposed trial to be acceptable overall, despite the potential barriers to success described above. The views of the two clinicians who found the trial 'not acceptable' in the survey appeared to shift in favour of the trial during their subsequent interview, during which the evidence which questioned the use Omeprazole was discussed and changes to the reflux treatment pathway were explained, including parent views.

For peer review only - http://bmjopen.bmj.com/site/about/guidelines.xhtml

Finally, our findings were considered against the adapted Theoretical Framework of Acceptability (TFA) for paediatric trials (10:9,18:522), which consists of eight component constructs (see **Table 3**).

Table 3 Adapted Theoretical Framework of Acceptability (10,18)

Construct	Definition
Affective attitude	How an individual feels about the intervention.
Burden	The perceived amount of effort that is required to participate in the intervention.
Ethicality	The extent to which the intervention has a good fit with an individual's value system
Intervention coherence	The extent to which the participant understands the intervention and how it works.
Opportunity costs	The extent to which benefits, profits, or values must be given up to engage in the intervention.
Perceived effectiveness	The extent to which the intervention is perceived likely to achieve its purpose.
Self-efficacy	The participant's confidence that they can perform the behaviour(s) required to participate in the intervention.
Trust*	The extent to which the participant (or parent/guardian) trusts those delivering the intervention to put the needs of patient before the requirements of the study

Analysis of feasibility study data indicates that five out of eight constructs of the TFA (affective attitude, burden, intervention coherence, self-efficacy and trust) for the TOAST trial were fully met for parents. Concerns of a minority related to the ethicality construct and the proposed Omeprazole dose (1mg/kg) being insufficient to treat reflux symptoms and potential side effects. The remaining constructs were largely met, or could be met, if suggestions for changes to the trial materials and protocol are addressed by the team.

Although almost all clinicians stated they found the proposed TOAST trial acceptable overall, only two out of seven constructs of the TFA (affective attitude and burden) were fully met for clinicians who completed the survey and three met (affective attitude, burden and opportunity costs) for those who took part in the focus group or interviews. As the themes presented in this paper highlight, wider issues impacted upon anticipated acceptability including: the ability to retain patients in the trial due to concerns about a potential 4-week timeframe to PPI for babies with symptomatic reflux; a change in practice; and the need to amend the inclusion criteria to make the trial more acceptable for some.

Discussion

This study provides insight into the acceptability of the proposed TOAST trial for parents and clinicians who care for infants with OA. Like other studies that highlight the value of feasibility work (11) and 'conducting pre-trial research with key stakeholders' (10:9,23) to improve recruitment and retention in clinical trials (23,24), involving parents and clinicians in this feasibility study provided valuable insight into potential barriers and solutions to recruitment and retention of infants in the TOAST trial.

For peer review only - http://bmjopen.bmj.com/site/about/guidelines.xhtml

Overall, the majority of parents and clinicians who took part in this feasibility study supported the proposed trial as they felt it would help address an area of clinical uncertainty. Parents and clinicians ranked the same top four outcomes (‘Severity of anastomotic stricture’, ‘Incidence of anastomotic stricture’, ‘Need for treatment of reflux’ and the ‘Presence of symptoms of reflux’) as important to measure for this study. Our findings highlight the need to carefully consider how symptomatic reflux would be treated in all trial participants. Although all parents found the use, dose and duration of omeprazole as the intervention medication and placebo control acceptable, some parents whose child had experienced signs of symptomatic reflux (9) had concerns about being able to access PPI if their child was in discomfort. Parents of children who had experienced commonly reported side effects of PPI, such as infections, wind or an upset stomach (2,4,6,8,9), or a previously unreported side effects, such as thick mucus that made breathing difficult, stated they would still hypothetically consent for their child to take part in the trial even if they had a 50/50 chance of receiving PPI.

Our findings show that despite clinicians stating that they found the trial acceptable, multiple constructs in the Theoretical Framework of Acceptability were not fully met due to concerns or perceived challenges to conducting the trial. Some were external factors that they felt parents may face, such as the ability of families to travel to follow-up appointments, or the pro-medication influence of TOFS Facebook group impacting on the views of new parents of OA infants invited to participate. As also found by others (25), most other challenges raised related to changing usual individual clinical practice, and for this study, clinician equipoise and specifically a wish to access PPI when children were showing signs of reflux, in line with existing guidelines (4). As described above, these findings echo the concerns of some parents. Refining the inclusion and exclusion criteria and developing a symptomatic reflux pathway that clinicians would find acceptable will be key to ensuring they are willing to enrol infants in their care into the trial. Whilst reviewing this pathway with parents during interviews, it became apparent that they also viewed the pathway as an important resource for parents in the trial, which may assist with participant retention. Many felt it would bring reassurance that babies ‘would not be left to suffer’ with symptoms of reflux if they took part in the TOAST trial. Parents supported the use of an ‘opt in’ mobile phone application that would send reminders to administer the trial intervention, as well as host the symptomatic reflux pathway and other related trial information, all of which may help with protocol adherence and help prevent withdrawal from the trial.

Previous research has shown the importance of identifying when is an appropriate time to discuss trial participation, as a poorly timed approach can cause additional burden for distressed families, which may also increase the likelihood that parents will decline trial participation (26,27). Parents stated that it would not be acceptable for clinicians to broach the trial on the day of surgery as it would be too overwhelming. Timepoints before surgery, when a baby is diagnosed with OA during pregnancy, and two to three days after surgery were recommended by both parents and clinicians.

We found that the views of a minority of clinicians whose survey responses suggested they did not find the trial acceptable appeared to shift in favour of the trial during subsequent interviews. During these conversations the evidence that indicated PPIs may increase stricture rates (6,7), parent views, and the proposed reflux treatment pathway were explained. This finding alone highlights content that should be included in staff training and trial resources, as well as wider findings that demonstrate parental support for the trial. Inclusion of a statement

For peer review only - http://bmjopen.bmj.com/site/about/guidelines.xhtml

on the treatment pathway which states that if clinicians 'feel that urgent treatment is needed, clinical judgement takes precedence' is also likely to help address concerns about the potential 4-week timeframe to administer PPI and therefore make the trial seem more acceptable. However, it is also important to recognise that such a statement may also lead to cross over between trial arms, or patient withdrawal, which should be closely monitored in the pilot trial phase.

Strengths and limitations of this study

A mixed methods approach including a survey, interviews and focus groups enabled comprehensive insight into key stakeholder views, as well as the ability to explore clinician concerns that were evident in the survey in more depth through interviews. Although 39 parents registered interest in an interview, nearly half did not respond to further correspondence and three cancelled due to their child being readmitted to hospital, which highlights the challenges of engaging parents of such vulnerable children in research. Despite the difficulties experienced in arranging interviews we continued to interview parents until the point of information power (1) and our study included parents with recent relevant experience. As the majority of parents were recruited through the TOFS support group, our sample may comprise of experienced parents with an interest in OA research and may not reflect the potential TOAST sample who will also have less awareness of PPI and treatment options for symptoms of reflux at the time the trial is discussed. The clinicians involved were overwhelmingly surgeons, with only two neonatologists taking part in the survey. However, as surgeons will predominantly be deciding which babies to approach for the TOAST trial, this is unlikely to a be significant limitation to assessing the feasibility of the trial.

Conclusions

All parents and most clinicians viewed the proposed trial as being feasible and acceptable, so long as infants can access PPI if clinically required. Our findings will inform the trial protocol for the internal pilot phase of the main trial as well as the main trial itself and site training materials to ensure the trial is family centred and to assist clinician engagement. Recruitment, retention and protocol adherence data should be closely monitored during the pilot phase to inform decisions about progression to a full trial.

Author contributions

All authors (except EN) were involved in the conception or design of the study; or the acquisition, analysis, or interpretation of data for the work. TM and KW collected the data. TM analysed and interpreted the data with oversight from KW. TM and KW drafted the article and all authors (except PH and DM) critically revised it for important intellectual content. All authors gave approval of the version to be published. TM and KW agree to be accountable for all aspects of the work in ensuring that questions related to the accuracy or integrity of any part of the work are appropriately investigated and resolved.

Competing interests' statement

National Institute for Health Research (NIHR) Health Technology Assessment (HTA) Programme grant payments to support the conduct of this study were made to the institutions of all authors (except JP). TKM and EN employment roles were funded by the NIHR HTA programme grant payment made to their institutions.

For peer review only - http://bmjopen.bmj.com/site/about/guidelines.xhtml

Funding statement

The TOAST feasibility study was funded by the National Institute for Health Research Health Technology Assessment Programme (project number NIHR 131136; link to Protocol: https://fundingawards.nihr.ac.uk/award/NIHR131136) and coordinated by the University of Liverpool. The views and opinions expressed herein are those of the authors and do not necessarily reflect those of the HTA Programme, NIHR, NHS or the Department of Health. The funders had no role in the collection, analysis and interpretation of data or in the decision to submit this article for publication.

Data availability statement

No data are available. The datasets generated and analysed for this study are not publicly available due to participants being from a small, specialised population, which creates ethical and privacy concerns about being too identifiable.

Acknowledgements

Special thanks are extended to TOFS parent advisory group members for their involvement in the design, conduct, progress and findings of the feasibility study and the development and conduct of the proposed trial. Gratitude is extended to Alan Downs[d] for his involvement in this study.

Supplementary material

This content has been supplied by the authors. It has not been vetted by BMJ Publishing Group Limited (BMJ) and may not have been peer-reviewed. Any opinions or recommendations discussed are solely those of the authors and are not endorsed by BMJ. BMJ disclaims all liability and responsibility arising from any reliance placed on the content. Where the content includes any translated material, BMJ does not warrant the accuracy and reliability of the translations (including, but not limited to, local regulations, clinical guidelines, terminology, drug names and drug dosages), and is not responsible for any error and/or omissions arising from translation and adaptation or otherwise.

Open access

This is an open access article distributed in accordance with the Creative Commons Attribution 4.0 International (CC BY 4.0) license, which permits others to copy, redistribute, remix, transform and build upon this work for any purpose, provided the original work is properly cited and appropriate credit given, a link to the licence is given, and indication of whether changes were made. See: https://creativecommons.org/licenses/by/4.0/.

Ethics approval

The TOAST feasibility study was approved by the University of Liverpool Research Ethics Committee (Reference number: 8510).

For peer review only

Reference list

1. Malterud K, Siersma VD, Guassora AD. Sample Size in Qualitative Interview Studies: Guided by Information Power. Qual Health Res. 2016;26(13):1753–60. DOI: 10.1177/1049732315617444
2. Allin B, Knight M, Johnson P, Burge D. Outcomes at one-year post anastomosis from a national cohort of infants with oesophageal atresia. PLoS One. 2014;9(8). DOI: 10.1371/journal.pone.0106149.
3. Tambucci R, Angelino G, De Angelis P, Torroni F, Caldaro T, Balassone V, Chiara Contini A., Romeo E, Rea F, Faraci S, Federici di Abriola G, Dall'Oglio L. Anastomotic Strictures after Esophageal Atresia Repair: Incidence, Investigations, and Management, Including Treatment of Refractory and Recurrent Strictures. In: Krishnan U, Faure C, editors. Update on Oesophageal Atresia-Tracheoesophageal Fistula. Frontiers in Pediatrics; 2017. p. 48–61. DOI:10.3389/978-2-88945-304-7
4. Krishnan U, Mousa H, Dall'Oglio L, Homaira N, Rosen R, Faure C, Gottrand F. ESPGHAN-NASPGHAN guidelines for the evaluation and treatment of gastrointestinal and nutritional complications in children with esophageal atresia-tracheoesophageal fistula. J Pediatr Gastroenterol Nutr. 2016;63(5):550–70. DOI: 10.1097/MPG.0000000000001401
5. Hagander L, Muszynska C, Arnbjornsson E, Sandgren K. Prophylactic treatment with proton pump inhibitors in children operated on for oesophageal atresia. Eur J Pediatr Surg. 2012;22(2):139–42. DOI: 10.1055/s-0032-1308698
6. Jones CE, Smyth R, Drewett M, Burge DM, Hall NJ. Association Between Administration of Antacid Medication and Anastomotic Stricture Formation After Repair of Esophageal Atresia. J Surg Res. 2020;254:334–9. DOI:10.1016/j.jss.2020.05.004.
7. Miyake H, Chen Y, Hock A, Seo S, Koike Y, Pierro A. Are prophylactic anti-reflux medications effective after esophageal atresia repair? Systematic review and meta-analysis. Pediatr Surg Int. 2018;34(5):491–7. DOI:10.1007/s00383-018-4242-4.
8. Santana RNS, Santos VS, Ribeiro-Júnior RF, Freire MS, Menezes MAS, Cipolotti R, Gurgel RQ. Use of ranitidine is associated with infections in newborns hospitalized in a neonatal intensive care unit: A cohort study. BMC Infect Dis. 2017;17(1):1–6. DOI: 10.1186/s12879-017-2482-x
9. National Institute for Health and Care Excellence (NICE). GORD in children: Omeprazole. GORD in children: Omeprazole. 2020 [cited 2022 May 19]. Available from: https://cks.nice.org.uk/topics/gord-in-children/prescribing-information/omeprazole/
10. Deja E, Peters MJ, Khan I, Mouncey PR, Agbeko R, Fenn B, Watkins J, Ramnarayan P, Tibby SM, Thorburn K, Tume LN, Rowan KM, Woolfall K. Establishing and augmenting views on the acceptability of a paediatric critical care randomised controlled trial (the FEVER trial): A mixed methods study. BMJ Open. 2021;11(3):1–11. DOI: 10.1136/bmjopen-2020-041952
11. O'Hara CB, Canter RR, Mouncey PR, Carter A, Jones N, Nadel S, Peters MJ, Lyttle MD, Harrison DA, Rowan KM, Inwald D, Woolfall K. A qualitative feasibility study to inform a randomised controlled trial of fluid bolus therapy in septic shock. Arch Dis Child. 2018;103(1):28–32. DOI: 10.1136/archdischild-2016-312515
12. Tong A, Sainsbury P, Craig J. Consolidated criteria for reporting qualitative research (COREQ): A 32-item checklist for interviews and focus groups. Int J Qual Heal Care. 2007;19(6):349–57. DOI: 10.1093/intqhc/mzm042
13. Deja E, Roper L, Tume LN, Dorling J, Gale C, Arch B, Latten L, Pathan N, Eccleson H, Hickey H, Preston J, Beissel A, Andrzejewska I, Valla FV, Woolfall K. Can they stomach it? Parent and practitioner acceptability of a trial comparing gastric residual volume measurement versus no gastric residual volume in UK NNU and PICUs: a feasibility study. Pilot Feasibility Stud. 2021;7(1):1–9. DOI: 10.1186/s40814-021-00784-5
14. Braun V, Clarke V. Reflecting on reflexive thematic analysis. Qual Res Sport Exerc Heal. 2019;11(4):589–97. DOI:10.1080/2159676X.2019.1628806.
15. Angen MJ. Evaluating interpretive inquiry: Reviewing the validity debate and opening

For peer review only - http://bmjopen.bmj.com/site/about/guidelines.xhtml

the dialogue. Qual Health Res. 2000;10(3):378–95. DOI: 10.1177/104973230001000308
16. Mays N, Pope C. Qualitative research in health care: Assessing quality in qualitative research. Br Med J. 2000;320(7226):50–2. DOI: 10.1136/bmj.320.7226.50
17. Sekhon M, Cartwright M, Francis JJ. Acceptability of health care interventions: A theoretical framework and proposed research agenda. Br J Health Psychol. 2018;23(3):519–31. DOI: 10.1111/bjhp.12295
18. QSR International. What is NVivo? Introducing the NVivo Suite. 2022 [cited 2022 May 19]. Available from: About NVivo (NVivo 11 for Windows) (qsrinternational.com)
19. Microsoft Corporation. Microsoft ® Excel ® 2019 MSO. Microsoft Office Professional Plus 2019.
20. Corbin J, Strauss A. Basics of qualitative research: Techniques and procedures for developing grounded theory. Fourth. London: SAGE Publications Ltd.; 2015. ISBN10 1412997461
21. Glaser BG. The Constant Comparative Method of Qualitative Analysis. Soc Probl. 1965;12(4):436–45. Available from: https://www.jstor.org/stable/798843
22. O'Cathain A, Thomas KJ, Drabble SJ, Rudolph A, Goode J, Hewison J. Maximising the value of combining qualitative research and randomised controlled trials in health research: The QUAlitative Research in Trials (QUART) study - A mixed methods study. Health Technol Assess (Rockv). 2014;18(38):1–197. DOI: 10.3310/hta18380
23. Tudur Smith C, Hickey H, Clarke M, Blazeby J, Williamson P. The trials methodological research agenda: Results from a priority setting exercise. Trials. 2014;15(32). DOI: 10.1186/1745-6215-15-32
24. Newlands R, Duncan E, Treweek SD, Elliott J, Presseau J, Bower P, Maclennan G, Ogden M, Witham M, Young B, Gillies K. The Development of Theory-Informed Participant-Centred Interventions to Maximise Participant Retention in Randomised Controlled Trials. Trials [Internet]. 2022;23(268):1–16. DOI: 10.21203/rs.3.rs-917451/v1.
25. Gupta DM, Boland RJ, Aron DC. The physician's experience of changing clinical practice: A struggle to unlearn. Implement Sci. 2017;12(1):1–11. DOI: 10.1186/s13012-017-0555-2
26. Woolfall K, Young B, Frith L, Appleton R, Iyer A, Messahel S, Hickey H, Gamble C. Doing challenging research studies in a patient-centred way: A qualitative study to inform a randomised controlled trial in the paediatric emergency care setting. BMJ Open. 2014;4(e005045). DOI: 10.1136/bmjopen-2014-005045
27. Woolfall K, Frith L, Gamble C, Gilbert R, Mok Q, Young B. How parents and practitioners experience research without prior consent (deferred consent) for emergency research involving children with life threatening conditions: A mixed method study. BMJ Open. 2015;5(9):1–14. DOI: 10.1136/bmjopen-2015-008522

For peer review only

For peer review only - http://bmjopen.bmj.com/site/about/guidelines.xhtml

TOAST

Treating Oesophageal Atresia to Prevent Stricture

TOAST feasibility study

Parent/Legal Representative Information Sheet

We invite you to take part in a research study

Following repair of oesophageal atresia some babies develop a narrowing at the site of the repair in the oesophagus called a stricture. Reflux is also very common after repair of oesophageal atresia and it is believed that reflux of acid into the oesophagus may make a stricture more likely or more severe.

Antacid medication is sometimes used to supress the acid produced by the stomach and help reduce the risk of strictures. Despite this treatment being used in about half of babies with oesophageal atresia, the evidence for using this medication to reduce the chance of getting a stricture is weak. We don't really know if it works. But we would like to find out because if it does then all babies with oesophageal atresia can be given this treatment and if it does not then we can stop asking parents to give medicine to their baby that has no benefit.

We want to answer the question, "Should babies born with oesophageal atresia all be treated routinely with antacid medication to reduce strictures?" To do this we are planning a clinical trial, which will be the first of its kind, to help inform the future treatment of babies with oesophageal atresia.

- To help find out what parents think about the trial and help to design it, we would like to interview about 25 parents/legal representatives with experience of infants born with oesophageal atresia within the last three years, including those with or without stricture and those who did or did not receive routine antacid medication
- By speaking to parents/ legal representatives we hope to find out whether the trial is acceptable and, if so, how it should be done.
- The interview should take about 30-45 minutes and can be done over the telephone or online (e.g. Zoom). Should you be interviewed, you will receive a £30 Amazon voucher to thank you for your time.

How to contact us

If you have any questions and/or would like to take part in an interview, please contact: Dr Tracy Mitchell Telephone: 07379 105134 Email: tracy.mitchell@liverpool.ac.uk Further information can be found on our website: ***https://www.npeu.ox.ac.uk/toast/parents***

Why are we doing this study?

Babies may suffer reflux after surgery where the acid content of the stomach comes back into the oesophagus and can cause regurgitation of feeds and/or damage to the oesophagus. Sometimes a narrowing of the tube (called a stricture) where the oesophagus was rebuilt may be caused or made worse by reflux. Some surgeons who look after babies with oesophageal atresia use a medication to suppress the acid produced by the stomach even if there are no symptoms of reflux. A major reason for this is to reduce the risk of strictures forming. Despite this being an apparently popular option (about half of babies with oesophageal atresia are treated with this medication) the evidence for using this medication is weak. In fact, some studies of babies with oesophageal atresia have actually found that strictures are more common in babies treated with acid suppression than in those who were not. In addition, there is some suggestion that taking the medicine can increase the chance of certain types of infection. Until we conduct a clinical trial we don't really know the best way to treat babies with oesophageal atresia.

For peer review only - http://bmjopen.bmj.com/site/about/guidelines.xhtml

We want to answer the question, "Should babies born with oesophageal atresia all be treated routinely with antacid medication to reduce strictures?" To do this we are planning a two-phased study. Firstly, we will assess the feasibility of the study. We will work with surgeons, other doctors looking after babies with oesophageal atresia, families of such babies and others who are involved in their care to explore what is important to them and whether they would be willing to take part in a trial where we test our question. Secondly, the findings from this will then be used to design a trial where babies with oesophageal atresia are allocated at random to either being given acid suppressing medicine or a placebo (an inactive substance that looks the same as the medicine). This is called a randomised controlled trial and is considered the "Gold Standard" in terms of answering clinical questions like this.

Who can take part?

Parents/legal representatives with experience of a baby with oesophageal atresia, including those with or without stricture and those who did or did not receive routine antacid medication in the last three years.

How can I take part?

If you would like to take part in an interview, please contact Tracy Mitchell or Kerry Woolfall by email or telephone (contact details are on the first page). If two parents/guardians in the same family wish to take part then the interviews will be conducted separately, one at a time.

Before the interview, we will send you an information sheet (like this one) which will be used to invite parents/guardians to take part in the TOAST clinical trial.

During the interview, Tracy will ask you what you think about the information sheet and what improvements you think we could make. We would also appreciate your views on the best way to speak to parents about this research, the design of the study (such as how and when medication is given to babies with reflux), our approach to seeking consent and your views on the parent / family centred things that we plan to observe as outcomes of the trial. With your permission we would like to record the interviews for analysis purposes but all names and identifying information will be removed. Interviews will be digitally recorded which will then be written out by the researcher or a transcription service. After the interview, we will send you a £30 Amazon voucher to thank you for your time.

The views of parents with experience infants born with oesophageal atresia are very important to make sure we design this study in a way that is acceptable to parents/guardians and ensure that they understand the study information given to them in such stressful circumstances.

Taking part is completely optional and you can change your mind about being part of the study at any time by contacting the study team.

If you have any questions before deciding to take part, please do not hesitate to contact us. The study results will be made available on the study website when the study is finished.

Are there any risks in taking part?

This is a low risk study. The majority of questions will be about the proposed clinical trial. However, at the beginning of the interview we will be inviting you to discuss your child's experiences, such as when was your child's oesophageal atresia first identified or diagnosed. And did your child experience a narrowing of their oesophagus following this surgery? Such personal questions about your child's experience may be upsetting for some. If you would rather not answer such questions, please let the interviewer know. You can decide not to answer a question at any time. The interview can also be paused or stopped at any time you wish. Details of additional support such as the TOFS (Tracheo-Oesophageal Fistula Support) group for parents are provided on the last page of this information sheet.

For peer review only - http://bmjopen.bmj.com/site/about/guidelines.xhtml

Who is involved in this study?

The study is being funded by the National Institute for Health Research (NIHR) which is the research arm of the NHS. Mr Nigel Hall (University of Southampton) and Mr Iain Yardley (Evelina Children's Hospital) are the TOAST Study Chief Investigators. At a later stage, it is planned that the the National Perinatal Epidemiology Unit, Clinical Trials Unit (NPEU CTU) at the University of Oxford (which has been central to previous UK-wide surveys of babies with Oesophageal Atresia) will conduct the trial. Dr Kerry Woolfall (University of Liverpool) is leading this element of the study. The TOAST research team are qualified to do this study because they have all the specialties and skills needed. Members of team have a lot of experience in caring for children with oesophageal atresia and are very active in health research. Parents of children oesophageal atresia and TOFS have been involved in the development of this study.

How will my data be used and what happens if I want to stop taking part?

The University of Liverpool is the sponsor for this study based in the United Kingdom. We will be using information you provide us with during interviews in order to undertake this study and will act as the data controller for this study. This means that we are responsible for looking after your information and using it properly. The University of Liverpool will keep identifiable information about you for 10 years after the study has finished.

Your rights to access, change or move your information are limited, as we need to manage your information in specific ways in order for the research to be reliable and accurate. Participation in the study is voluntary and you are free to withdraw without explanation up until your data has been anonymised (approximately a week after the interview). All identifiable information removed and saved anonymously.

The University processes personal data as part of its research and teaching activities in accordance with the lawful basis of 'public task', and in accordance with the University's purpose of "advancing education, learning and research for the public benefit." Under UK data protection legislation, the University acts as the Data Controller for personal data collected as part of the University's research. Dr Kerry Woolfall, acts as the Data Processor for this study, and any queries relating to the handling of your personal data can be sent to Dr Kerry Woolfall, University of Liverpool (Tel: 0151 794 4634).

Further information on how your data will be used can be found in the table below.

How will my data be collected?	Digital recording of interviews.
How will my data be stored?	Password protected data files.
How long will my data be stored for?	10 years
What measures are in place to protect the security and confidentiality of my data?	Any identifiable information will be taken out of the interviews when written out and each participant will be assigned a number. Consent forms will be securely stored.
Will my data be anonymised?	All identifiable information will be removed, and a number assigned to each participant.
How will my data be used?	Findings will be written up for the funders NIHR. Publication will be sought with peer reviewed journals.
Who will have access to my data?	Tracy Mitchell and Kerry Woolfall
Will my data be archived for use in other research projects in the future?	No
How will my data be destroyed?	Shredded or deleted after 10 years

For peer review only - http://bmjopen.bmj.com/site/about/guidelines.xhtml

What if there is a problem?

If you are unhappy, or if there is a problem, please feel free to let us know by contacting **Kerry Woolfall, Telephone: 0151 794 4634** and we will try to help. If you remain unhappy or have a complaint which you feel you cannot come to us with then you should contact the Research Ethics and Integrity Office at ***ethics@liv.ac.uk***. When contacting the Research Ethics and Integrity Office, please provide details of the name or description of the study (so that it can be identified), the researcher(s) involved, and the details of the complaint you wish to make. The University strives to maintain the highest standards of rigour in the processing of your data. However, if you have any concerns about the way in which the University processes your personal data, it is important that you are aware of your right to lodge a complaint with the Information Commissioner's Office by calling 0303 123 1113. For NHS service advice or support please contact: Patient Advice and Liaison Services (PALS) services. Go to ***www.nhs.uk*** to find your local PALS contact details. There is also the TOFS (Tracheo-Oesophageal Fistula Support) support group for parents:

www.tofs.org.uk/about-us.aspx

Telephone +44 (0)115 961 3092

Email: ***info@tofs.org.uk***

Who has reviewed the study?

The study has been reviewed by the University of Liverpool Research Ethics Committee (REC Ref. 8510) who have agreed that the study is being conducted in a correct and appropriate manner.

Thank you for your time.
We are very grateful that you are considering taking part in this study.

For peer review only

TOAST Study Team

NPEU Clinical Trials Unit, University of Oxford,
Old Road Campus, Headington, Oxford, OX3 7LF.
01865 617919
toast@npeu.ox.ac.uk
www.npeu.ox.ac.uk/toast

TOAST is funded by the National Institute for Health Research (NIHR) Health Technology Assessment programme (project reference 131136). The views expressed are those of the author(s) and not necessarily those of the NIHR or the Department of Health and Social Care.

For peer review only - http://bmjopen.bmj.com/site/about/guidelines.xhtml

TOAST Practitioner Questionnaire

Thank you for agreeing to help us with a study assessing the feasibility of a proposed RCT into the use of proton pump inhibitor (PPI) medication (Omeprazole) to reduce anastomotic stricture formation following oesophageal atresia repair. We would like to explore your attitudes and opinions about the study concept, the trial design and ways in which we can make the project more likely to succeed. This questionnaire, which will take approximately 15 minutes to complete, will explore your views on the proposed trial design, including questions to assess your views on trial acceptability, the inclusion/exclusion criteria, and your willingness to be involved in the TOAST trial.

Your responses are important to us and will help to refine the trial protocol. Please note that by completing this survey you are giving permission for your responses to be included in the NIHR funded TOAST study. All information will be held by the University of Liverpool, anonymised and stored securely in compliance with UK GDPR.

If you have any questions, please email Dr Tracy Mitchell: Tracy.Mitchell@liverpool.ac.uk or Dr Kerry Woolfall: woolfall@liverpool.ac.uk. Further information about the study can be found here: https://www.npeu.ox.ac.uk/toast/clinicians

It is recommended that you download the 'Treatment plan for treating babies with symptoms of reflux' flowchart before commencing this questionnaire to be able to see the text more clearly, especially if completing this questionnaire on your mobile phone: https://www.npeu.ox.ac.uk/assets/downloads/toast/pathway.pdf

Once you have read and understood each statement, please click on the box to indicate consent.

* Required

1. I confirm that I have read and understood the information sheet. I have had the opportunity to consider the information, ask questions and have these answered satisfactorily *

Check all that apply.

☐ Yes

2. I agree to participate in the questionnaire *

Check all that apply.

☐ Yes

3. I understand that my responses will remain confidential and will not be used for any other purpose *

Check all that apply.

- [ ] Yes

4. I understand that I am free to withdraw from the study at any time without reason, and if so, all of my information will be disposed of *

Check all that apply.

- [ ] Yes

5. I understand that the results from the study may be published in an academic journal and that it will be kept anonymous *

Check all that apply.

- [ ] Yes

6. I agree to my data being stored on a computer at University of Liverpool for 10 years in line with the standard university procedure *

Check all that apply.

- [ ] Yes

Please read this outline of the proposed Treating Oesophageal Atresia to Prevent Stricture (TOAST) trial before completing the questionnaire

As you know, babies born with oesophageal atresia (OA) may have gastro-oesophageal reflux after surgery to repair their oesophagus. The link between reflux and the development of anastomotic stricture is contentious. Some surgeons who look after babies with oesophageal atresia use proton pump inhibitor (PPI) medication to suppress the acid produced by the stomach even if there are no symptoms of reflux. A major reason for this is to reduce the risk of strictures forming. Despite this being an apparently popular option (about half of babies with oesophageal atresia are treated with PPI medication) the evidence for using this medication is weak. In fact, some studies of babies with oesophageal atresia have actually found that strictures are more common in babies treated with PPI medication than in those who were not. In addition, there is some suggestion that taking the PPI medication can increase the chance of certain types of infection.

The primary objective of the Treating Oesophageal Atresia to Prevent Stricture (TOAST) study is to address the research question 'In babies born with oesophageal atresia does routine use of PPI medication reduce the severity or incidence of anastomotic stricture compared to placebo?'

To do this we aim to perform a multicentre double blinded randomised placebo-controlled trial (with integral internal pilot and health economic evaluation) of babies with type C oesophageal atresia (i.e., only those with distal fistula) in which half will receive PPI from the time of repair until 1 year of age and the other half will receive placebo. The primary outcome of the TOAST study is to assess the severity of anastomotic stricture through the number of dilatations performed within the first year of life, and we intend to measure a range of other relevant and meaningful outcomes for up to two years. We have selected type C only so as to have a reasonably homogenous population and because this represents the majority of babies born with OA.

The research will take place in specialist U.K. neonatal surgical units and in participant homes; it is expected that approximately 12-15 units will take part. An 18-month internal pilot phase incorporates "stop-go" criteria to evaluate feasibility of recruitment and other trial processes. The trial aims to recruit 211 infants in 90 months from the recruitment start date, equating to around 3-4 infants per month across all participating sites. Eligible infants will be recruited early in life, around the time of their surgical repair, so that the trial intervention can commence as soon as possible after surgical repair. Where a diagnosis of oesophageal atresia is made antenatally, parents may be approached regarding the trial before birth. Eligible infants will be identified by the clinical care team and recruited by appropriately trained, delegated individuals. These infants will be randomised to receive PPI medication or a placebo, daily, until one year of age.

Trial data collection will be from trial entry until 24 months of age, including screening, consent, randomisation and follow-up. Outcome information will be collected by case report forms (CRFs), paper and electronic, with clinical data collection from medical records at hospital sites and parent reported outcomes. Participant compliance with administering PPI medication or placebo will be performed via a parent report App. The App may also be used for communication to parents about data collection, reportable safety events and other trial communications. Data will be collected when the child is 3, 6, 9, 12, 18, and 24 months of age on primary care contacts, out of pocket expenses, time away from work, and parental health related quality of life using the EuroQol EQ-5D-5L questionnaire. Children's health related quality of life using the parent completed PedsQL questionnaire will be collected at 24 months.

We have designed this trial to be as pragmatic as possible, so that data are recorded during routine hospital visits as much as possible and there is little interference with your standard follow-up pathways. There key points that are important to the successful running of the trial are as follows:

- The trial will be blinded so that none of surgeons, nurses, nor parents will know which treatment is being given.
- The dose of PPI we plan to use will be 1mg/kg/day. During the trial the use

For peer review only - http://bmjopen.bmj.com/site/about/guidelines.xhtml

of other gastric acid suppression medication will generally not be allowed (e.g., other PPI or ranitidine) since we wish to maintain separation between the trial groups. To allow for symptoms of reflux in babies to be treated we have designed a pathway for escalating treatment of reflux symptoms for use in this trial. This will also prevent inadvertent overdosing with PPI.

• During the trial, we will be discouraging the use of 'routine' dilatations / calibrations, reserving these only for when an infant is symptomatic. However, we will be leaving the definition of symptomatic to clinician judgement.

The schedule of events for the proposed trial are summarised in the flow chart below.

Flow chart: Treating Oesophageal Atresia to prevent STricture (TOAST)

Inclusion criteria

- Infants with oesophageal atresia with distal trache-oesophageal fistula undergoing primary repair at the first operative intervention in the newborn period
- Written informed parental consent

Exclusion criteria

- Infants undergoing staged repair or delayed primary repair or requiring emergency ligation of trache-oesophageal fistula with primary repair later
- No realistic prospect of survival

Randomisation (1:1 allocation ratio)

Routine anti-reflux medication

Esomeprazole 0.5mg/kg whilst infants are nil by mouth, followed by omeprazole 1mg/kg

Given once daily from trial entry for one year

OR

Placebo

Given once daily from trial entry for one year

Primary Outcome

Severity of anastomotic stricture
(defined as number of dilatations performed to one year of age)

Secondary Outcomes

Measured at 3, 6, 9, 12, 18 and 24 months after trial entry

- Incidence of anastomotic stricture (0 vs 1 or more dilatations)
- Other complications directly related to oesophageal atresia
- Adverse events
- Presence of symptoms of gastro-oesophageal reflux
- Need for treatment of reflux
- Growth
- Respiratory symptoms
- Oral intake
- Episodes of infection
- Mortality
- Maternal Health Related Quality of Life (EQ-5D-5L)
- Child Health Related Quality of Life (PedsQL; parent reported, collected at age 24 months)
- Healthcare and societal costs
- Incremental cost per dilation prevented
- Other meaningful outcomes proposed during the feasibility phase

Longer-term follow up

Permission will be requested to access trial participants' healthcare and educational records for the measurement of longer-term outcomes using routinely collected data. This activity would be as part of a separately funded study.

TOAST flow chart 280721

For peer review only - http://bmjopen.bmj.com/site/about/guidelines.xhtml

Part 1 Background Information

7. Are you actively involved in the pre/post-surgical care of children with oesophageal atresia (OA) in the United Kingdom? *

Mark only one oval.

- Yes *Skip to question 8*
- No
  Skip to section 4 (Thank you for your interest in the TOAST study. Unfortunately, this study is only involving those actively involved in the pre or post surgical care of children with oesophageal atresia (OA) in the United Kingdom.)

Thank you for your interest in the TOAST study. Unfortunately, this study is only involving those actively involved in the pre or post surgical care of children with oesophageal atresia (OA) in the United Kingdom.

8. Which UK hospital do you work at? *

____

9. What is your job role? *

Mark only one oval.

- Surgeon *Skip to question 11*
- Neonatologist *Skip to question 11*
- Neonatal surgical specialist nurse *Skip to question 11*
- Other *Skip to question 10*

10. Please type your job role here *

____

11. Approximately how many babies with oesophageal atresia do you treat/are under your own individual care each year? *

12. Do you have any experience of recruitment to clinical trials? *

Mark only one oval.

- Yes *Skip to question 13*
- No *Skip to question 14*

13. How many years' experience do you have in recruiting to clinical trials? *

14. Do you routinely administer or prescribe prophylactic proton pump inhibitors following surgery in ALL babies with type C oesophageal atresia under your care? *

Mark only one oval.

- Yes *Skip to question 15*
- No *Skip to question 16*

15. Please can you explain your reasons for prescribing prophylactic PPIs following surgery? *

Skip to question 17

16. Please can you explain your reasons for not prescribing prophylactic PPIs following surgery? *

Part 2: Inclusion and exclusion criteria

The primary objective of the Treating Oesophageal Atresia to Prevent Stricture (TOAST) study is to address the research question 'In babies born with oesophageal atresia does routine use of PPI medication reduce the severity or incidence of anastomotic stricture compared to placebo?' TOAST is a multicentre double blinded randomised placebo-controlled trial of babies with type C oesophageal atresia (i.e., only those with distal fistula) in which half will receive PPI from the time of repair until 1 year of age and the other half will receive placebo.

Our proposed inclusion criteria are:
- Infants with OA with distal tracheoesophageal fistula (TOF) undergoing primary repair at the first operative intervention.
- Written parental informed consent

17. How satisfied are you with these inclusion criteria? *

Mark only one oval.

- Very satisfied
- Satisfied
- Neutral
- Not satisfied
- Not at all satisfied

18. Do you have any comments or suggested changes for proposed inclusion criteria?

Our proposed exclusion criteria are:

- Infants undergoing staged repair, delayed primary repair or requiring emergency ligation of tracheoesophageal atresia with primary repair later.
- Infants with no realistic chance of survival beyond the new-born period.

19. How satisfied are you with these exclusion criteria? *

Mark only one oval.

- Very satisfied
- Satisfied
- Neutral
- Not satisfied
- Not at all satisfied

20. Do you have any comments or suggested changes for the proposed exclusion criteria?

Part 3: Trial question and acceptability

21. Do you think the proposed trial is addressing an important research question? *

Mark only one oval.

- Yes *Skip to question 22*
- No *Skip to question 23*

22. Please explain why you think that the proposed trial is addressing an important research question? *

____
____
____
____
____

Skip to question 24

23. Please explain why you think that the proposed trial is not addressing an important research question *

____
____
____
____
____

The proposed intervention is esomeprazole (0.5mg/kg intravenously) once daily whilst infants are nil by mouth followed by omeprazole (1mg/kg orally) once daily until one year of age.

24. How acceptable do you think the use of this PPI medication is in the intervention arm of the TOAST trial? *

Mark only one oval.

- Very acceptable
- Acceptable
- Neutral
- Not acceptable
- Not at all acceptable

25. How acceptable do you think the dose of 1mg/kg of omeprazole orally once daily is? *

Mark only one oval.

- Very acceptable
- Acceptable
- Neutral
- Not acceptable
- Not at all acceptable

26. How acceptable do you think the duration of one year for intervention delivery is? *

Mark only one oval.

- Very acceptable
- Acceptable
- Neutral
- Not acceptable
- Not at all acceptable

27. How acceptable would you find administering the PPI medication to babies in your care? *

Mark only one oval.

- Very acceptable
- Acceptable
- Neutral
- Not acceptable
- Not at all acceptable

28. Please elaborate on your answers for the proposed intervention medication

The comparator to esomeprazole/omeprazole is a matched placebo (e.g., saline/cellulose/water solution with no active ingredient) given intravenously once daily whilst infants are nil by mouth followed by oral administration, once daily, until one year of age.

29. How acceptable do you think the use of a placebo is in the comparator arm of the TOAST trial? *

Mark only one oval.

- Very acceptable
- Acceptable
- Neutral
- Not acceptable
- Not at all acceptable

30. How acceptable would you find administering a placebo to babies in your care? *

Mark only one oval.

- Very acceptable
- Acceptable
- Neutral
- Not acceptable
- Not at all acceptable

31. Please elaborate on your answers for the proposed placebo

32. Do you think the TOAST trial is practically possible to conduct? *

Mark only one oval.

- Yes
- No

33. How acceptable do you think it is to conduct TOAST Trial? *

Mark only one oval.

- Very acceptable
- Acceptable
- Neutral
- Not acceptable
- Not at all acceptable

34. How acceptable would you find randomising babies with OA under your care into the TOAST trial? *

Mark only one oval.

- Very acceptable
- Acceptable
- Neutral
- Not acceptable
- Not at all acceptable

For peer review only - http://bmjopen.bmj.com/site/about/guidelines.xhtml

35. Please elaborate on any of your responses above

36. How acceptable do you feel it is to give PPI medication to half the babies who take part in this study? *

Mark only one oval.

- Very acceptable
- Acceptable
- Neutral
- Not acceptable
- Not at all acceptable

37. How acceptable do you think it is to NOT give PPI medication to half the babies in this study? *

Mark only one oval.

- Very acceptable
- Acceptable
- Neutral
- Not acceptable
- Not at all acceptable

38. Please elaborate on your answers about the acceptability of giving/not giving PPI medication to half the babies in this study

____

____

____

____

____

Treatment plan for babies with symptoms of reflux

We have developed an escalating treatment plan (please see below, or if you did not download a copy before starting this questionnaire, you can this link: https://tinyurl.com/38n4wjtv) for babies with symptoms of reflux drawing on the best available evidence for the management of reflux. Babies with reflux symptoms would first be managed with changing feed frequencies and positioning advice escalating to feed thickener (if formula feed) or Gaviscon (if breast fed) and only allowed to have additional medication (e.g., omeprazole) if these fail and symptoms are felt to be having a significant impact.

TOAST

Evidence - and guideline - based rational decision tree for treating possible reflux symptoms in postoperative OA/TOF

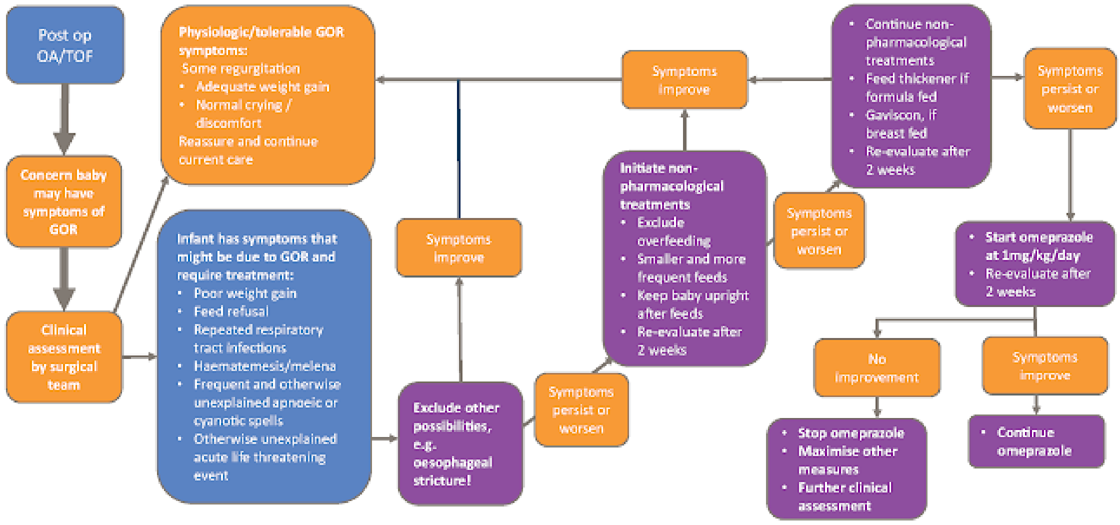

N.B. If you feel that urgent treatment is needed, clinical judgement takes precedence over the above.

39. How acceptable do you find this escalating treatment plan for babies under your care who have symptoms of reflux in the trial? *

Mark only one oval.

- Very acceptable
- Acceptable
- Neutral
- Not acceptable
- Not at all acceptable

40. Would you be happy to follow this escalating treatment plan for babies under your care who have symptoms of reflux in the trial? *

Mark only one oval.

- Yes
- No

41. Please elaborate

42. Does the pathway to managing reflux for babies in the trial address any concerns or raise any concerns for you? *

Mark only one oval.

- Yes
- No

43. Please elaborate and include whether anything was unclear or missing from the treatment pathway flow chart, or any changes that you would like to suggest.

Part 4: Trial outcomes and follow up

As we have discussed, in the TOAST study we want to find out if routinely giving babies PPI suppression medication after repair of oesophageal atresia will reduce the incidence or severity of oesophageal stricture. To do this we will collect information on outcomes such as those listed below:

TOAST TRIAL: OUTCOMES

Primary Outcome

1. Severity of anastomotic stricture

• *The number of dilations performed within one year of trial entry (Measured at 3, 6, 9, 12 months).*

Secondary Outcomes (Measured up to the first two years of life)

2. Incidence of anastomotic stricture

• *Whether the baby has any dilatation or none in the first year / 2 years of their life.*

3. All-cause mortality (up to 12 and 24 months)

• *Whether the baby survived to a certain time point (usually time point at months/years) or to a specific event (e.g., hospital discharge).*

4. Number of complications directly related to oesophageal atresia post-surgery

• *Anything that is directly related to oesophageal atresia or its repair, for instance anastomotic leak or recurrent fistula.*

5. Adverse events

• *A general term used to describe things that don't go as planned but aren't included in other outcomes.*

6. Presence of symptoms of gastro-oesophageal reflux

• *Symptoms that may be due to reflux and reported by parents (e.g., using a reflux symptom score, measured every three months).*

7. Treatment of reflux symptoms

• *Whether the baby is given any sort of medication to treat reflux symptoms (e.g., feed thickener, Gaviscon, Omeprazole).*

8. Weight (standard deviation score)

• *To monitor the baby's growth during the first year of their life (recorded by weight, length and head circumference at certain time points).*

9. Respiratory symptoms

• *Presence of respiratory symptoms such as infections, chronic cough (but not the usual TOF cough) or any other symptoms that a doctor felt required investigation or treatment.*

10. Oral feed intake

• *How the baby is progressing with advancing from milk feeds onto solids at 12 and 24-months using a validated measure (e.g., IDDSI score).*

11. Number of courses of antibiotics prescribed for episodes of infection

• *(including respiratory infections).*

12. Maternal health related quality of life (EQ-5D-5L)

• *A measure of a mother's quality of life using a specially designed questionnaire to understand how their baby's health may impact on their health and quality of life.*

13. Child health related quality of life (PedsQL; Parent reported, collected at age 24 months)

• *A measure of the baby's quality of life, as reported by a parent. This is measured using a specially designed questionnaire and is done at 2 years of age because this is the youngest age that it can reliably be done.*

44. Please rank three outcomes that you feel are most important to be measured in the TOAST trial in order of importance. The first most important outcome for the TOAST study is: *

Mark only one oval.

- Severity of anastomotic stricture
- Incidence of anastomotic stricture
- All-cause mortality
- Number of complications directly related to oesophageal atresia post-surgery
- Adverse events
- Presence of symptoms of gastro-oesophageal reflux
- Treatment of reflux symptoms
- Weight
- Respiratory symptoms
- Oral feed intake
- Number of courses of antibiotics prescribed for episodes of infection
- Maternal health related quality of life
- Child health related quality of life

45. The second most important outcome for the TOAST study is: *

Mark only one oval.

- Severity of anastomotic stricture
- Incidence of anastomotic stricture
- All-cause mortality
- Number of complications directly related to oesophageal atresia post-surgery
- Adverse events
- Presence of symptoms of gastro-oesophageal reflux
- Treatment of reflux symptoms
- Weight
- Respiratory symptoms
- Oral feed intake
- Number of courses of antibiotics prescribed for episodes of infection
- Maternal health related quality of life
- Child health related quality of life

46. The third most important outcome for the TOAST study is: *

Mark only one oval.

- Severity of anastomotic stricture
- Incidence of anastomotic stricture
- All-cause mortality
- Number of complications directly related to oesophageal atresia post-surgery
- Adverse events
- Presence of symptoms of gastro-oesophageal reflux
- Treatment of reflux symptoms
- Weight
- Respiratory symptoms
- Oral feed intake
- Number of courses of antibiotics prescribed for episodes of infection
- Maternal health related quality of life
- Child health related quality of life

47. Are there any other outcomes that you think are important primary or secondary outcomes to measure for this trial that are not included in the list above?

Mark only one oval.

- Yes *Skip to question 48*
- No *Skip to question 51*

48. Please provide your suggestion for an additional outcome here and state whether it is a primary or secondary outcome *

49. Please provide your suggestion for an additional outcome here and state whether it is a primary or secondary outcome

50. Please provide your suggestion for an additional outcome here and state whether it is a primary or secondary outcome

For peer review only - http://bmjopen.bmj.com/site/about/guidelines.xhtml

Follow up is currently planned to occur at 3, 6, 9 and 12 months to coincide with routine clinic visits and some will be measured though parental reports via an App. You will be free to see these infants more often should you wish. This will include asking specifically about the treatment of anastomotic strictures and more generally about other symptoms and adverse effects.

51. Is there anything you feel should be specifically addressed in follow up? *

Mark only one oval.

- Yes *Skip to question 52*
- No *Skip to question 53*

52. Please elaborate *

Part 5: Recruitment and consent

We propose that babies will be randomised into the medication or placebo group within 72 hours of their oesophageal surgery, when their condition has stabilised.

53. How acceptable do you think it is to approach parents to discuss the trial at this point in time? *

Mark only one oval.

- Very acceptable
- Acceptable
- Neutral
- Not acceptable
- Not at all acceptable

54. Please elaborate

55. Would you have any concerns about approaching parents to seek consent for their baby's inclusion in TOAST? *

Mark only one oval.

- Yes
- No

56. Please elaborate

57. How concerned are you about the ability to retain participants in the TOAST trial? *

Mark only one oval.

- Not at all concerned
- A little concerned
- Very concerned

58. If you have any concerns about the retention of participants in the TOAST trial and/or any suggestions to help address concerns about the retention of participants, please elaborate below

59. Do you envisage any practical or logistical challenges in delivering TOAST at your unit? *

Mark only one oval.

- Yes
- No

60. Please elaborate

Part 6: Overall acceptability and training

61. Overall, how acceptable do you find the proposed TOAST trial? *

Mark only one oval.

- Very acceptable
- Acceptable
- Neutral
- Not acceptable
- Not at all acceptable

62. Please elaborate

63. Is there anything specific that you would suggest we include in the TOAST site training package? (e.g., training content, who should be trained, whether any online resources would be needed in addition to site initiation visit) *

64. Do you have any other comments or concerns about the TOAST trial? *

Mark only one oval.

- Yes *Skip to question 65*
- No *Skip to section 27 ()*

65. Please note any other comments or concerns about the TOAST trial here

We plan to conduct further research into clinicians' attitudes to the trial via more in depth focus group or telephone interview discussions. These will be held online via Zoom or a similar platform. Please add your contact details if you would like to register interest in taking part in a focus group or interview. Your details will not be used for any other purposes and will be stored separately from questionnaire data

First name and email address

66. First name

67. Email address

Thank you for your participation in this survey.

This content is neither created nor endorsed by Google.

Google Forms

COREQ (COnsolidated criteria for REporting Qualitative research) Checklist

A checklist of items that should be included in reports of qualitative research. You must report the page number in your manuscript where you consider each of the items listed in this checklist. If you have not included this information, either revise your manuscript accordingly before submitting or note N/A.

Topic	Item No.	Guide Questions/Description	Reported on Page No.
**Domain 1: Research team and reflexivity**			
Personal characteristics			
Interviewer/facilitator	1	Which author/s conducted the interview or focus group?	3-4
Credentials	2	What were the researcher's credentials? E.g. PhD, MD	3
Occupation	3	What was their occupation at the time of the study?	3
Gender	4	Was the researcher male or female?	3
Experience and training	5	What experience or training did the researcher have?	3
Relationship with participants			
Relationship established	6	Was a relationship established prior to study commencement?	Supp File 1
Participant knowledge of the interviewer	7	What did the participants know about the researcher? e.g. personal goals, reasons for doing the research	Supp File 1
Interviewer characteristics	8	What characteristics were reported about the inter viewer/facilitator? e.g. Bias, assumptions, reasons and interests in the research topic	Supp File 1
**Domain 2: Study design**			
Theoretical framework			
Methodological orientation and Theory	9	What methodological orientation was stated to underpin the study? e.g. grounded theory, discourse analysis, ethnography, phenomenology, content analysis	3-4
Participant selection			
Sampling	10	How were participants selected? e.g. purposive, convenience, consecutive, snowball	3
Method of approach	11	How were participants approached? e.g. face-to-face, telephone, mail, email	3
Sample size	12	How many participants were in the study?	4
Non-participation	13	How many people refused to participate or dropped out? Reasons?	4
Setting			
Setting of data collection	14	Where was the data collected? e.g. home, clinic, workplace	4
Presence of non-participants	15	Was anyone else present besides the participants and researchers?	N/A
Description of sample	16	What are the important characteristics of the sample? e.g. demographic data, date	2-3, 4-5
Data collection			
Interview guide	17	Were questions, prompts, guides provided by the authors? Was it pilot tested?	3-4
Repeat interviews	18	Were repeat inter views carried out? If yes, how many?	N/A
Audio/visual recording	19	Did the research use audio or visual recording to collect the data?	4
Field notes	20	Were field notes made during and/or after the inter view or focus group?	N/A
Duration	21	What was the duration of the inter views or focus group?	4
Data saturation	22	Was data saturation discussed?	4
Transcripts returned	23	Were transcripts returned to participants for comment and/or	N/A

Topic	Item No.	Guide Questions/Description	Reported on Page No.
		correction?	
**Domain 3: analysis and findings**			
Data analysis			
Number of data coders	24	How many data coders coded the data?	4
Description of the coding tree	25	Did authors provide a description of the coding tree?	N/A
Derivation of themes	26	Were themes identified in advance or derived from the data?	4
Software	27	What software, if applicable, was used to manage the data?	4
Participant checking	28	Did participants provide feedback on the findings?	3 (PPI)
Reporting			
Quotations presented	29	Were participant quotations presented to illustrate the themes/findings? Was each quotation identified? e.g. participant number	5-10
Data and findings consistent	30	Was there consistency between the data presented and the findings?	5-13
Clarity of major themes	31	Were major themes clearly presented in the findings?	5-10
Clarity of minor themes	32	Is there a description of diverse cases or discussion of minor themes?	N/A

Developed from: Tong A, Sainsbury P, Craig J. Consolidated criteria for reporting qualitative research (COREQ): a 32-item checklist for interviews and focus groups. *International Journal for Quality in Health Care*. 2007. Volume 19, Number 6: pp. 349 – 357

Once you have completed this checklist, please save a copy and upload it as part of your submission. DO NOT include this checklist as part of the main manuscript document. It must be uploaded as a separate file.

For peer review only

TOAST

Treating Oesophageal Atresia to Prevent Stricture

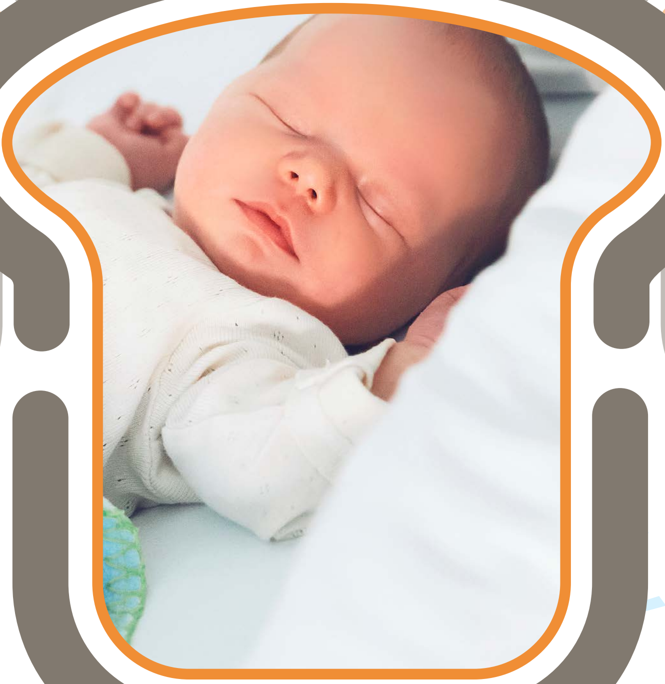

Parent Information Leaflet

For peer review only - http://bmjopen.bmj.com/site/about/guidelines.xhtml

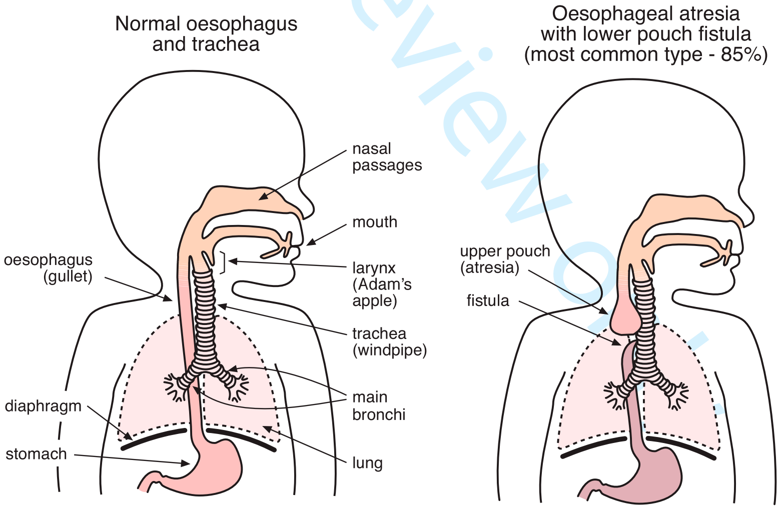

From Jaffray, B. (2016) 'Introduction to OA/TOF', in Martin, V. and Crabbe, D. (Eds.) The TOF Book. 2nd Edition. Nottingham, UK: TOFS., p. 17. ISBN 978-0-9536265-1-9. Copyright 2016 by TOFS. Reprinted with permission.

TOAST

We understand that this is a difficult time for you and your family. We would like to invite you to take part in a study this hospital is taking part in for babies who have been born with oesophageal atresia. Before you decide it is important that you understand why the research is being done and what it will mean for you and your baby. Please ask us if there is anything else that is not clear or you would like to know.

Why do we do research in healthcare?

Research is really important so that we can improve how we treat patients. If no research took place, then it would be difficult to improve outcomes for patients. All research in the NHS is voluntary.

Why are we being asked to take part?

Your baby has been diagnosed with oesophageal atresia, where the oesophagus (swallowing tube) has not formed properly and so your baby's mouth is not connected to their stomach. The treatment for this condition involves surgery to reconstruct the oesophagus. The team looking after your baby will have talked to you about what this surgery involves.

We are asking you to help us with a research project to learn more about how treatment following this surgery can be made better. The study is called TOAST which stands for Treating Oesophageal Atresia to prevent STricture.

Why are we doing this research?

One of the complications that can follow surgery to reconstruct the oesophagus is a narrowing called a stricture at the point where the repair is made. A stricture in the oesophagus would make it difficult for your baby to feed. Between 40% and 50% of babies get a stricture in the months after repair of oesophageal atresia. A stricture is usually treated by stretching the narrow section to make it wider in a procedure called a "dilatation". This requires an admission to hospital and is done under general anaesthetic. Some babies require several dilations during the first year of life.

Gastro-oesophageal reflux (where the stomach contents go back up into the

For peer review only - http://bmjopen.bmj.com/site/about/guidelines.xhtml

oesophagus) is also very common after repair of oesophageal atresia and some surgeons think that reflux of acid into the oesophagus may make a stricture more likely or more severe. To try and prevent strictures from forming some surgeons use gastric acid suppression medication (e.g. omeprazole, 1ml/kg/day) in all babies, even if they have no symptoms of reflux, after repair of oesophageal atresia. The aim of using this medicine is to reduce stomach acid secretion so that even if there is reflux it is not as acidic.

Despite the common use of gastric acid suppression medication, we do not know for certain if there is any benefit to its use in babies following surgery. Indeed, some studies have indicated that babies routinely given gastric acid suppression medication may be more likely to get a stricture, but the evidence is not conclusive. There are other reasons why giving gastric acid suppression medication may not be a good idea, including that they may slightly increase the risk of infections and concerns about giving medicines to babies without proven benefit.

For these reasons, we are carrying out the TOAST study to find out if giving gastric acid suppression medication does help babies with oesophageal atresia. If it does, then all babies with oesophageal atresia can be given this treatment. If it does not, then we can stop asking parents to give medicine to their baby that has no benefit.

What will happen if we take part?

The study we are asking you to give permission for your child to take part in is a randomised controlled trial (RCT). In the trial half of the babies will be given an gastric acid suppression medication from soon after their surgery to repair the oesophageal atresia and the other half will be given a cellulose/water solution as a 'dummy' medication called a placebo. We will use a computer to decide at random whether your baby receives gastric acid suppression medication or the placebo. The chance of your baby receiving either gastric acid suppression medication or placebo is equal. No one will know whether your baby is receiving the gastric acid suppression medication or the placebo. This includes you, the medical team and the research team. The reason a placebo is important is to make sure we are carrying out a fair test.

Your baby will be given the treatment each day by the medical team in hospital. When your baby is discharged home, we will ask you to give the treatment to your baby once daily for one year after their surgical repair. The doctors and nurses will show you how to do this. You will be asked to complete questionnaires about your baby's progress, how much treatment you have given, hospital visits and how you are feeling. These will be completed through an app, online or

For peer review only - http://bmjopen.bmj.com/site/about/guidelines.xhtml

on paper if you prefer. The doctors and nurses will provide you with further treatment supplies at your routine hospital visits and will be available to answer any questions you might have.

We will then follow your baby up for the first two years of their life to see if they develop a stricture and to monitor their progress. You will be asked to complete questionnaires at 3, 6, 9, 12, and 18 months, and 2 years about your baby's progress, how much treatment you have given, hospital visits and how you are feeling. These will be completed through an app, online or on paper if you prefer. Your baby will be followed up for the duration of their childhood whether they take part in this study or not and we will use these routine follow up visits to collect information relevant to the study so you will have the minimum inconvenience. If your baby does develop a stricture then they will need treatment and your doctors will explain this to you. At the end of the study in around 2028 we hope to have included about 211 babies born with oesophageal atresia. This is a number which we believe is big enough to give convincing results, and is far bigger than reported in any similar studies done worldwide to date.

We have tried to make taking part in the study as easy as possible for you and your family. The follow up has been designed around routine follow up visits that would happen for all babies born with oesophageal atresia so it should not mean extra visits to the hospital.

What are the possible benefits or risks of taking part?

The treatments used in the study are used routinely for babies born with oesophageal atresia in UK hospitals and are known to be safe.

This study will not bring any immediate benefit to your child. We hope that we will get information about how best to treat children born with oesophageal atresia in the future. By participating, you will be helping us to learn whether treatment with gastric acid suppression medication is better than none so that we will know whether to offer this routinely as a treatment for other children in the future. After we have finished the study, we can let you know the results if you would like.

Do we have to take part?

No, taking part is completely voluntary. If you decide not to take part, you do not have to give a reason and your child's care will not be affected in any way. If you agree to take part, we will ask you to sign a consent form which indicates your agreement to take part in the study and to let the researchers look at your child's health records. We will put a copy of this research consent form in your

For peer review only - http://bmjopen.bmj.com/site/about/guidelines.xhtml

child's health records. We will give you a copy for your files.

How long do I have to decide?

Because we would like to start treating your child soon after their operation to repair the oesophagus we will need to know if you are willing to take part within the next few days. To help you decide please talk about the study to your doctor. Please ask them any questions you may have.

What if I change my mind?

You can change your mind about taking part at any time and remove your baby from the study without giving a reason. We would then only use the information we have collected up to the point when you withdraw. This would not affect the care your baby receives in any way.

Will my details be kept confidential?

Your GP will be told that your baby is taking part in this study. We will collect personal information about you and your baby; the details will be kept securely and will only be seen by the research team, the study organisers in Oxford and people from the sponsor or regulatory authorities who check on studies such as this - this is a routine process of research in the NHS.

All information that we collect about you and your baby during the study will be kept strictly confidential and stored securely. We might ask for permission to contact you again in the future to find out how your baby is getting on as they grow up. If you decide to take part, we will collect some personal information about you and your baby, including name, NHS number, date of birth, address and contact details (e.g., email address and telephone number). This information will be sent to the Study Coordinating Centre at the University of Oxford, National Perinatal Epidemiology Unit Clinical Trials Unit (NPEU CTU). Authorised members of the research team will hold this data and store it securely for the purposes of running the trial. Authorised staff from the University of Oxford (as Coordinating Centre and Sponsor), funder, regulatory bodies, and your hospital may be given access to data for monitoring and/or audit of the study to ensure the research is complying with applicable regulations. Personal identifiable information including your telephone number and email address will be shared with Blue Frontier App provider for the purposes of recording the amount of medication given. The Study Coordinating Centre in Oxford will keep identifiable information about you and your baby from this study for 25 years after the study has finished. For more information on how we process and protect you and your baby's data, please see our website: ***https://www.npeu.ox.ac.uk/ctu/privacy-notice***

For peer review only - http://bmjopen.bmj.com/site/about/guidelines.xhtml

Further information can also be found at the NHS Health Research Authority's website:

https://www.hra.nhs.uk/planning-and-improving-research/policies-standards-legislation/data-protection-and-information-governance/gdpr-guidance/templates/template-wording-for-generic-information-document/

Please see our website for further information about how we protect your data: ***https://www.npeu.ox.ac.uk/ctu/privacy-notice***

Who is organising and funding the study?

The study is funded by the National Institute of Health Research (NIHR) which is the research arm of the NHS. The study is sponsored by the University of Oxford and is being run by the National Perinatal Epidemiology Unit Clinical Trials Unit (NPEU CTU) at the University of Oxford. The researchers involved in the study are all experienced researchers, interested in improving the care given to babies born with oesophageal atresia. A number of parents have also helped design the study, as have TOFS, the UK support group for families affected by oesophageal atresia.

All research in the NHS is looked at by an independent group of people, called a Research Ethics Committee, to protect your interests and make sure it is done to the highest standards. This trial has been reviewed and given favourable opinion by a Research Ethics Committee.

What will happen to the results of the research study?

At the end of the study, the results will be analysed and published in a medical journal. We will write our reports in a way that no one can work out who took part in the study. We will also share the results at medical and surgical conferences and with families of children with oesophageal atresia via the TOFS support group, who have been involved in setting up the trial. We will send you a copy of the results at the end of the study and we will also share them on our website. Unidentifiable data from this study may be shared with other groups who are carrying out similar work in the future.

What happens if I have a complaint because of my baby taking part in this study?

In the first instance you can talk to the clinical team looking after your baby who will help you with your concern. You can also contact the local research team, either the research nurses or the principal investigator, their contact details are on the back page of this leaflet. If you wish to complain about any aspect of

For peer review only

For peer review only - http://bmjopen.bmj.com/site/about/guidelines.xhtml

the way that you or your baby has been treated you may use the normal National Health Service complaints procedures, the Patient Advice and Liaison Service at your hospital will advise you about this (their contact details are also on the back page of this leaflet).

In the unlikely event your baby has been harmed by taking part in this study, you may have grounds for legal action and could seek compensation through the Research Sponsor, the University of Oxford, who has appropriate insurance-related arrangements in place. If your baby is harmed and it is due to any routine clinical treatment or negligence then the usual NHS indemnity arrangements will apply.

Contact Information

Chief Investigators:

Mr Nigel Hall, University of Southampton

Mr Iain Yardley, Evelina London Children's Hospital.

Local Contact Details:

Principal Investigator:

{_LEAD_}

Thank you for taking the time to read this information sheet

TOAST Study Team

NPEU Clinical Trials Unit,
National Perinatal Epidemiology Unit, University of Oxford,
Old Road Campus, OXFORD, OX3 7LF

01865 617919 toast@npeu.ox.ac.uk www.npeu.ox.ac.uk/toast

For more information about the TOFS support group please visit their website:
www.tofs.org.uk/about-us.aspx

FUNDED BY NIHR | National Institute for Health Research

TOAST is funded by the National Institute for Health Research (NIHR) Health Technology Assessment programme (project reference 131136).
The views expressed are those of the author(s) and not necessarily those of the NIHR or the Department of Health and Social Care.

For peer review only - http://bmjopen.bmj.com/site/about/guidelines.xhtml

TOAST

Evidence - and guideline - based rational decision tree for treating possible reflux symptoms in postoperative OA/TOF

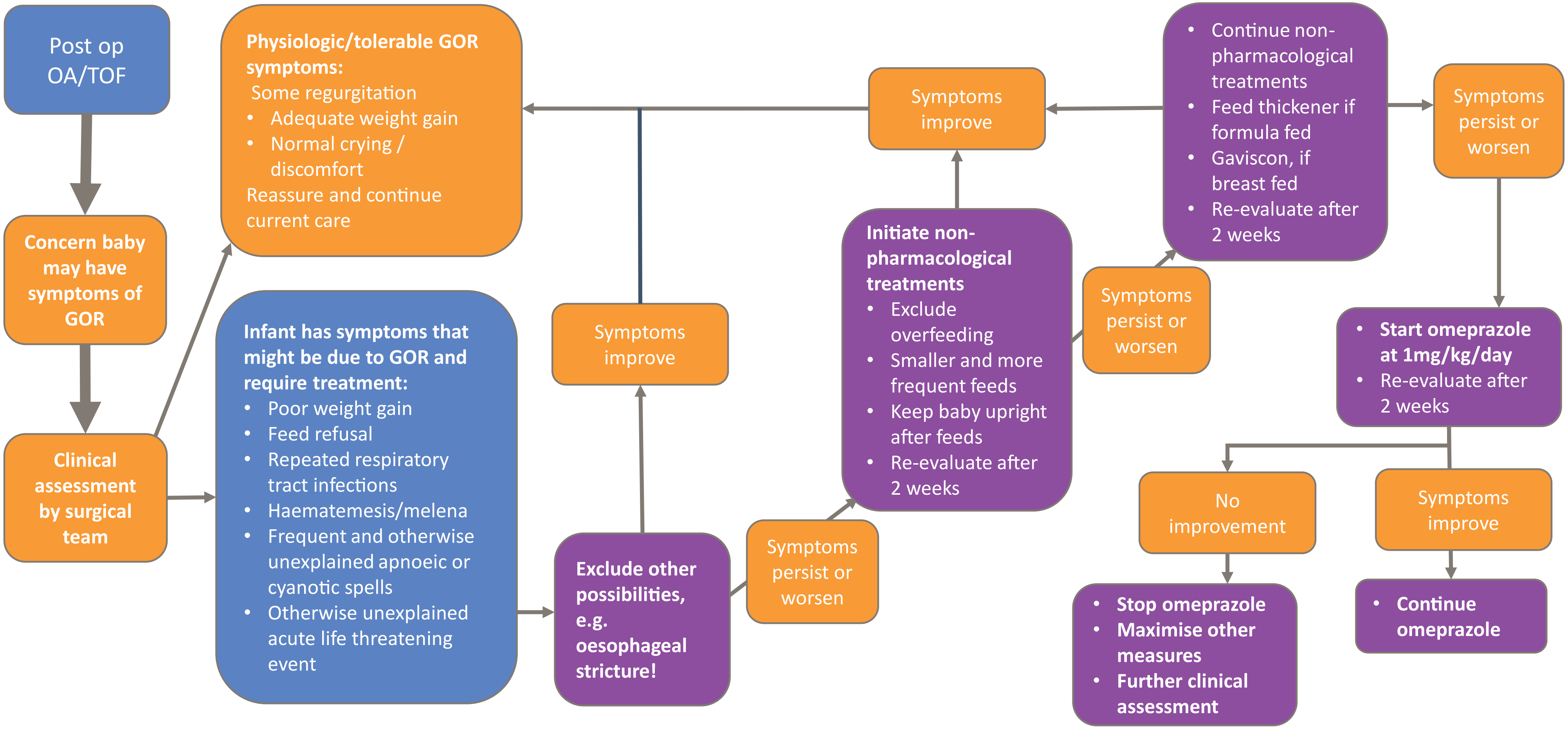

N.B. If you feel that urgent treatment is needed, clinical judgement takes precedence over the above.

TOAST
Treating Oesophageal Atresia to Prevent Stricture

Toast Trial: Outcomes

Severity of anastomotic stricture
- The number of dilations performed within one year of trial entry.

Incidence of anastomotic stricture
- Whether your baby has any dilatation or none in the first year / 2 years of their life.

Other complications directly related to oesophageal atresia
- Anything that is directly related to oesophageal atresia or its repair, for instance leakage from the join in the oesophagus.

Adverse events
- A general term used to describe things that don't go as planned but aren't included in other outcomes.

Presence of symptoms of gastro-oesophageal reflux
- Symptoms that may be due to reflux and reported by parents.

Need for treatment of reflux
- Whether your baby is given any sort of medication to treat reflux (e.g., feed thickener, Gaviscon).

Growth
- Your baby's growth during the first year of their life (recorded by weight, length and head circumference at certain time points).

Respiratory symptoms
- Presence of respiratory symptoms such as infections, chronic cough (but not the usual TOF cough) or any other symptoms that a doctor felt required investigation or treatment.

Oral Intake
- How your baby is progressing with advancing from milk feeds onto solids at specific ages.

Episodes of infection
- Any episode of proven infection (including respiratory infections).

Survival
- Whether your child survived to a certain time point (usually time point at months/years) or to a specific event (e.g., hospital discharge).

Maternal Health Related Quality of Life (EQ-5D-5L)
- A measure of a mother's quality of life using a specially designed questionnaire to understand how their baby's health may impact on their health and quality of life.

Child Health Related Quality of Life (PedsQL; Parent reported, collected at age 24 months)
- A measure of your baby's quality of life, as reported by a parent. This is measured using a specially designed questionnaire and is done at 2 years of age because this is the youngest age that it can reliably be done.

TOAST Study Team
NPEU Clinical Trials Unit, University of Oxford,
Old Road Campus, Headington, Oxford, OX3 7LF.
01865 617919 toast@npeu.ox.ac.uk
www.npeu.ox.ac.uk/toast

TOAST is funded by the National Institute for Health Research (NIHR) Health Technology Assessment programme (project reference 131136). The views expressed are those of the author(s) and not necessarily those of the NIHR or the Department of Health and Social Care.

For peer review only - http://bmjopen.bmj.com/site/about/guidelines.xhtml

SUPPLEMENTARY FILE 7

Table 1 Parent suggested edits to the symptomatic reflux treatment pathway

In the Blue box ***Infant has symptoms that might be due to GOR and require treatment*** six parents suggested the following be added:<br>• *'Clear visible pain'*/ *'clear discomfort'* (P4, mother)/ being really *'uncomfortable'* (P3; P5, mothers) or having a *'sore tummy'* (P9, mother) - *'arching their back'* (P3; P4; P6, mothers), *'pushing their tummy out'* (P5, mother) or *'squirming'* (P3; P5, mothers).<br>• *'Coughing'* (P12; P3, mothers) (not the usual TOF cough) and *'retching'* (P3, mother).<br>• *'Abnormal'* (P4, mother) or *'extreme'* (P3, mother) *'crying'* (P4; P5, mothers) or *'screaming'* (P4, mother).<br>• *'Not settling after feeds'* (P5, mother).<br>• *'Bringing up milk, even after holding them up for half an hour'* (P6, mother).<br>• *'Dry nappies'* (P9, mother).
**In the purple *Exclude other possibilities, e.g., oesophageal stricture box***, add *'if they [GOR symptoms] are in combination with other usual stricture signs*' (P5, mother) (albeit, some parents did not receive much information about (P3, mother) or know what a stricture was at the beginning of their journey with a child with OA (P3; P9; P10, mothers). Some mothers did not know the signs of stricture (see below) to look out for (P5; P9, mothers). Furthermore, some parents were not told the word *stricture* or *dilatation* – terms such as *'narrowing'* or *'tightening'* (P12, mother) and *'stretch'* were used instead (P9, mother). Parents said that the other difficulty is that most signs of stricture are similar to those of reflux, such as:<br>• Coughing (P3; P5, mothers) or choking (P5; P15, mothers; P8, father), refusing (P5, mother), or not being able to swallow milk, food (P8, father; P12, mother) or saliva (P11, mother).<br>• Milk coming out of their nose (P5, mother).<br>• Not finishing milk feeds (P3, mother).<br>• Vomiting after feeds (P8; P17, fathers; P9; P13; P15, mothers).<br>• 'Blue events' when their child stopped/were struggling to breathe when trying to feed or lying down (P4; P5; P9; P12, mothers; P16; P18, fathers), or upset (P18, father):
**Have a further box under *Start omeprazole - symptoms improve*** for when children *'are better than [they were but are] still having a problem'* (P10, mother) and need the dosage increasing (P5, mother). Four mothers (P4; P5; P10; P12) felt that *'Omeprazole at 1mg per kilogram a day… [is] still quite a low dose of Omeprazole'* (P4, mother), with one child being *'started on 2mg per kilogram when he was in hospital'* (P12, mother) and another being on *'30mg per day … Why would they not pursue 'increase to maximum dose' and then 'symptoms improve, continue.' 'No improvement, stop Omeprazole.' Why stop the treatment before you've reached the maximum dose?'* (P4, mother).
Under ***Initiate Non-Pharmacological Treatments***, as well as holding the baby upright after feeds (P3; P5, mothers; P16, father), add:<br>• *'Keep stopping every five sucks'* if bottle fed to pace the feed (P3, mother).<br>• Remove *'animal milk protein from the mother's diet if the child is being breastfed, or from the formula, if the child is formula-fed'* (P4, mother).<br>• *'Incline the bed'* (P12, mother; P16, father)/*'Tip your cot at an angle'* (P4, mother) to ensure that the crib/cot/bed is higher at the head end (P10, mother). *'Things like propping them up and wedges … are standard things for a lot of TOFS children that might not get mentioned in a medical sense'* (P11, mother).

For peer review only - http://bmjopen.bmj.com/site/about/guidelines.xhtml

• *'Any of the above [reflux] symptoms when parent has already initiated non-pharmacological treatment'* (P4, mother).
Explain acronyms such as *'GOR'* (P9; P10; P14, mothers; P18, father) or to have a *'key to terms'* (P12, mother) at the bottom of the diagram.<br>Explain medical terminology such as haematemesis/melena and unexplained apnoeic or cyanotic spells – perhaps word these as *'blue events'* (P12, mother), *'life-threatening events'* (P5, mother) or *'desaturations'* (P3, mother) because *'different hospitals … use slightly different terms'* (P12, mother) and some parents did not know what these were when worded this way (P3; P10, mothers), even though they learn *'pretty quickly'* (P10) what these are.
Consider accessibility:<br>*'Some people are colour blind and might find it hard to read on all the different colours', so it might be best to make the colours a darker shade to increase the contrast between the colours and the text'* (P13, mother).<br>*Left align all text (rather than some being 'centred')* (P13, mother).<br>Send the arrow from the first *Symptoms improve* box directly to the Physiologic/tolerable GOR symptoms box.

For peer review only - http://bmjopen.bmj.com/site/about/guidelines.xhtml

Table 2 Clinician suggested edits to the symptomatic reflux treatment pathway

'I think the signs in the blue box need splitting into minor and minor. For example, poor weight gain or food refusal' (C20, surgeon, survey).
'Good flow chart - maybe mention in other conditions: consider tracheomalacia, as this would mimic reflux closely, but in some cases may require urgent surgical intervention' (C22, surgeon, survey).
'I think it should be more explicitly pro breast feeding. Breastfeeding should come above artificial feeding in each list. Some may advocate investigation (e.g., pH study, contrast swallow etc) at some point. Where will that fall? Feeding in a more upright position as well as nursing after feeding in an upright position may help. In the normal physiology box, you could add something about regular bowels and wet nappies (to be reassuring)' (C41, neonatologist, survey).
'Not clear in pathway when formal evaluation of GOR (pH/impedance)' (C46, surgeon, survey).
'In the pathway you have put exclude oesophageal stricture before excluding overfeeding and keeping the baby upright after feeds. I think this is the wrong way round because excluding stricture would involve endoscopy or contrast - not sure you would go to that step before you prop the baby up a little?' (C35, surgeon, survey). This quote supports that of one mother who said *'Obviously with the reflux and the GOR and things like that, they will go off and do the contrast studies… diagnosing whether reflux is an issue or not'* (P11, mother).
'What if a baby has gross clinical reflux / BRUEs consistent with severe reflux? Normally I would maximise PPI before taking the next steps? What if I perform a fundoplication in the first year of life?' (C4, surgeon, survey).
'What about anti-reflux surgeries? At what point would these be considered?' (C27, surgeon, survey).

For peer review only - http://bmjopen.bmj.com/site/about/guidelines.xhtml

SUPPLEMENTARY FILE 8 Edits and additions for predefined outcomes

Predefined outcome	Suggested edits or additions
'Presence of symptoms of gastro-oesophageal reflux'	Collect data on tube feeding:<br>*[Child's name]'s reflux was a lot worst when he had the nasogastric tube and when the tube come out his instances of vomiting dramatically decreased. So, if children go home with a nasogastric tube, it might be interesting as part of the study to see the ones that had the antacid medicine and the ones that didn't to see the sort of effects of both* (P13, mother, interview).<br>Collect data on the *'number of [hospital] admissions related to GER'* (C25, surgeon, survey).
'Oral intake' and 'Growth'	Measure the age of solid food intake (P4; P15, mothers, interviews; P17, father, interview).<br>Collect data on *'the use or non-use of a gastrostomy'* (P17, father, interview), '*nasogastric tube'* (P13, mother, interview) or *'jejunal feeding without fundoplication'* (C47, surgeon, survey) because *'neonatologists and paediatricians will be… interested in feeding, nasogastric tube feeding and growth parameters'* (C41, neonatologist, focus group 2) / *'weight gain velocity'* (C21, surgeon, survey). '*That is the first question that comes into your mind when you are thinking about the quality of life for those children during the first and second year of their life'* (C41, neonatologist, focus group 2).
'Respiratory symptoms'	*'Add bronchiolitis'* (P15, mother, interview).<br>Measure *'any near-death attacks'* (C25, surgeon, survey) / *'ALTE'* [apparent life-threatening event/BRUE – brief, resolved, unexplained event] (C47, surgeon, survey).
'Episodes of infection'	*'Add bronchiolitis'* (P15, mother, interview) and aspiration (P5; P9, mothers, interviews; P18, father, interview):<br>*'You already have respiratory symptoms as an outcome but I wonder if evidence of aspiration would be better because it is really aspiration that we are worried about in the small infant post OA/TOF repair who may have reflux'* (C35, surgeon, survey).<br>Gastrointestinal infections:<br>*'G.I. infection including NEC later'* (C21, surgeon, survey).<br>*'Sepsis/G.I. infections. There is evidence of increased risk of sepsis in neonates who are given PPIs so I think it would be important to look at this specifically'* (C40, surgeon, survey).<br>*'Gastro [episodes of diarrhoea and vomiting] … because of the lack of acid it [PPI] gives bugs, which will be going into the gut first'* (C39, surgeon, focus group 2).
'Child health related quality of life'	Measure the time that children are hospitalised for (P11; P12, mothers, interviews), child's quality of sleep (P10; P11, mothers, interviews) and child's mental health:<br>'*His mental wellbeing is just as important as his physical wellbeing. In preventing strictures or reducing the severity of a stricture, ultimately, you're reducing hospital stays, you're reducing stress. He developed a fear of hospitals when he was about nine months old. As soon as he would see the entrance, he'd just be an absolute mess'* (P12, mother, interview).

For peer review only - http://bmjopen.bmj.com/site/about/guidelines.xhtml

SUPPLEMENTARY FILE 8 Edits and additions for predefined outcomes

	Measure *'long term outcomes including Barrett's oesophagus'* (C11, surgeon, survey).<br>*'I think longer term follow-up would also be important as a secondary outcome at 5 and 10 years'* (C16, surgeon, survey).<br>Also see the edits or additions in 'Oral intake' and 'Growth' as these are overlapping.
'Maternal Health Related Quality of Life'	Amend to measure *'Parental'* health related quality of life (P12; P13, mothers, interviews):<br>*I don't think it should be limited to mothers, because obviously dads go through the same thing. I think it should be parental health related quality of life, rather than maternal. Actually, I work and my husband stays at home and looks after [Child's name] ... We don't have traditional gender roles at home. There are probably more families like that as well, so it's probably worth including dads, grandparents or whoever it is. That's probably the only thing I'd change is that it was parental rather than maternal* (P12, mother, interview).<br>*Maybe not just maternal, maybe it needs to be parental because it has an effect on certainly both parents* (P13, mother, interview).
'Other complications directly related to oesophageal atresia'	*'Other surgery – aortopexy / tracheopexy'* (C49, surgeon, survey).
'Severity of anastomotic stricture'	*'Anastomotic tension'* (C14, surgeon, survey).<br>Overlapping with 'Respiratory symptoms':<br>*'Any near-death attacks'* (C25, surgeon, survey).<br>*'ALTE'* [apparent life-threatening event/BRUE – brief, resolved, unexplained event] (C47, surgeon, survey).
'Need for treatment of reflux'	*'Fundoplication'* (C47, surgeon, survey)<br>*'Other surgery – fundoplication'* (C49, surgeon, survey).

For peer review only - http://bmjopen.bmj.com/site/about/guidelines.xhtml

BMJ Open

A mixed methods feasibility study to inform a randomised controlled trial of proton pump inhibitors to reduce strictures following neonatal surgery for oesophageal atresia

Journal:	*BMJ Open*
Manuscript ID	bmjopen-2022-066070.R1
Article Type:	Original research
Date Submitted by the Author:	04-Jan-2023
Complete List of Authors:	Mitchell, Tracy; University of Liverpool, Public Health, Policy and Systems<br>Hall, Nigel; University of Southampton Faculty of Medicine, University Surgery Unit<br>Yardley, Iain; Evelina Children's Hospital, Guy's & St. Thomas's NHS Foundation Trust,<br>Cole, Christina; University of Oxford, National Perinatal Epidemiology Unit, Clinical Trials Unit, Nuffield Department of Population Health<br>Hardy, Pollyanna; University of Oxford, Clinical Trials<br>King, Andy; University of Oxford,<br>Murray, David; University of Oxford, National Perinatal Epidemiology Unit, Clinical Trials Unit, Nuffield Department of Population Health<br>Nuthall, Elizabeth; University of Oxford, National Perinatal Epidemiology Unit, Clinical Trials Unit, Nuffield Department of Population Health<br>Roehr, Charles; University of Oxford, National Perinatal Epidemiology Unit, Clinical Trials Unit, Nuffield Department of Population Health<br>Stanbury, Kayleigh; National Perinatal Epidemiology Unit, Clinical Trials Unit, Nuffield Department of Population Health, University of Oxford, Old Road Campus<br>Williams, Rachel; University of Oxford, National Perinatal Epidemiology Unit, Clinical Trials Unit, Nuffield Department of Population Health<br>Pearce, John; TOFS<br>Woolfall, Kerry; University of Liverpool, Institute of Population, Health and Society
**Primary Subject Heading**:	Paediatrics
Secondary Subject Heading:	Gastroenterology and hepatology, Qualitative research, Research methods
Keywords:	QUALITATIVE RESEARCH, Paediatric gastroenterology < PAEDIATRICS, STATISTICS & RESEARCH METHODS, NEONATOLOGY, Neonatal intensive & critical care < INTENSIVE & CRITICAL CARE

SCHOLARONE™
Manuscripts

I, the Submitting Author has the right to grant and does grant on behalf of all authors of the Work (as defined in the below author licence), an exclusive licence and/or a non-exclusive licence for contributions from authors who are: i) UK Crown employees; ii) where BMJ has agreed a CC-BY licence shall apply, and/or iii) in accordance with the terms applicable for US Federal Government officers or employees acting as part of their official duties; on a worldwide, perpetual, irrevocable, royalty-free basis to BMJ Publishing Group Ltd ("BMJ") its licensees and where the relevant Journal is co-owned by BMJ to the co-owners of the Journal, to publish the Work in this journal and any other BMJ products and to exploit all rights, as set out in our licence.

The Submitting Author accepts and understands that any supply made under these terms is made by BMJ to the Submitting Author unless you are acting as an employee on behalf of your employer or a postgraduate student of an affiliated institution which is paying any applicable article publishing charge ("APC") for Open Access articles. Where the Submitting Author wishes to make the Work available on an Open Access basis (and intends to pay the relevant APC), the terms of reuse of such Open Access shall be governed by a Creative Commons licence – details of these licences and which Creative Commons licence will apply to this Work are set out in our licence referred to above.

Other than as permitted in any relevant BMJ Author's Self Archiving Policies, I confirm this Work has not been accepted for publication elsewhere, is not being considered for publication elsewhere and does not duplicate material already published. I confirm all authors consent to publication of this Work and authorise the granting of this licence.

For peer review only

A mixed methods feasibility study to inform a randomised controlled trial of proton pump inhibitors to reduce strictures following neonatal surgery for oesophageal atresia

Tracy K. Mitchell [a], Nigel J. Hall [b], Iain Yardley [c], Christina Cole [d], Pollyanna Hardy [d], Andy King [d], David Murray [d], Elizabeth Nuthall [d], Charles C. Roehr [d], Kayleigh Stanbury [d], Rachel Williams [d], John Pearce [e], Kerry Woolfall [a]

Academic affiliations

[a] Institute of Population Health, Department of Public Health, Policy and Systems, University of Liverpool, UK.
[b] University Surgery Unit, Faculty of Medicine, University of Southampton, UK
[c] Evelina Children's Hospital, London, UK; Faculty of Life Sciences and Medicine, King's College London, UK
[d] National Perinatal Epidemiology Unit, Nuffield Department of Population Health, University of Oxford, UK
[e] TOFS, Nottingham, UK

Email addresses

tracy.mitchell@liverpool.ac.uk
n.j.hall@soton.ac.uk
Iain.Yardley@gstt.nhs.uk
christina.cole@npeu.ox.ac.uk
pollyanna.hardy@npeu.ox.ac.uk
andy.king@npeu.ox.ac.uk
david.murray@npeu.ox.ac.uk
elizabeth.nuthall@npeu.ox.ac.uk
charles.roehr@npeu.ox.ac.uk
kayleigh.stanbury@npeu.ox.ac.uk
rachel.williams@npeu.ox.ac.uk
johnpearcetofs@googlemail.com
woolfall@liverpool.ac.uk

ORCID IDs

Tracy Karen Mitchell 0000-0003-0014-8016
Nigel J. Hall 0000-0001-8570-9374
Iain Yardley 0000-0002-6928-2267
Christina Cole 0000-0002-8798-2136
Pollyanna Hardy 0000-0003-2937-8368
Andrew R. King 0000-0001-5180-7179
David Murray 0000-0001-9010-2905
Elizabeth Nuthall 0000-0002-5092-7643
Charles C. Roehr 0000-0001-7965-4637
Kayleigh Stanbury 0000-0002-8726-2411
Rachel Williams 0000-0002-5872-1690
Kerry Woolfall 0000-0002-5726-5304

For peer review only

Corresponding author

Tracy Mitchell, Public Health, Policy, and Systems, Institute of Population Health, University of Liverpool, Room 213, Second Floor, Whelan Building, Quadrangle, Liverpool L69 3GB, UK; tracy.mitchell@liverpool.ac.uk Twitter @trakmitch

Key words

Qualitative research, Paediatric gastroenterology, Paediatric surgery, Statistics and research methods, Neonatology, Neonatal intensive and critical care

Abstract

Objectives: This mixed methods feasibility study aimed to explore parents' and medical practitioners' views on the acceptability and design of a clinical trial to determine whether routine prophylactic proton pump inhibitors (PPI) reduce the incidence of anastomotic stricture in infants with oesophageal atresia (OA).

Design: Semi-structured interviews with UK parents of an infant with OA and an online survey, telephone interviews and focus groups with clinicians. Data were analysed using reflexive thematic analysis and descriptive statistics.

Participants We interviewed 18 parents of infants with OA. Fifty-one clinicians (49 surgeons, 2 neonatologists) from 20/25 (80%) units involved in OA repair completed an online survey and 10 took part in one of two focus groups. Interviews were conducted with 2 clinicians whose survey responses indicated they had concerns about the trial.

Results: All parents and most clinicians found the use, dose and duration of omeprazole as the intervention medication, and the placebo control, as acceptable. Parents stated they would hypothetically consent to their child's participation in the trial. Concerns of a few parents and clinicians about infants suffering with symptomatic reflux, and the impact of this for study retention, appeared to be alleviated through the symptomatic reflux treatment pathway. Hesitant clinician views appeared to change through discussion of parental support for the study and by highlighting existing research that questions current practice of PPI treatment. Parents and clinicians ranked the same top four outcomes as important to measure for this study.

Conclusions Our findings indicate that parents and most clinicians view the proposed TOAST trial to be feasible and acceptable so long as infants can be given PPI if clinicians deem it clinically necessary. This insight into parent and clinician views and concerns will inform pilot phase trial monitoring, staff training and the development of the trial protocol.

Article summary

Strengths and limitations of this study

- A mixed methods approach including a survey, interviews and focus groups enabled comprehensive insight into key stakeholder views.
- Despite the difficulties experienced in arranging interviews we continued to interview parents until the point of information power and to involve parents of infants with OA at all stages, including study design and conduct, as members of the study team.

For peer review only - http://bmjopen.bmj.com/site/about/guidelines.xhtml

- Our sample may comprise of experienced parents with an interest in OA research and may not reflect the potential TOAST sample who will also have less awareness of PPI and treatment options for symptoms of reflux at the time the trial is discussed.
- Our study includes the perspectives of clinicians involved in the treatment of OA representing the majority of UK surgical units.

Introduction

Oesophageal atresia (OA) is a rare congenital anomaly that affects a baby's oesophagus, where the upper part of the oesophagus does not connect with the lower part. As this is life threatening, surgery is usually carried out shortly after birth. Approximately 150 babies are born with OA annually in the United Kingdom (UK) [1]. Stricture (narrowing) at the anastomosis (new connection) is the most common post-operative complication in the months following surgical repair [1,2], which requires admission to hospital for investigation and dilatation of the narrowed segment under general anaesthesia [2].

Some international guidelines [3] recommend routine proton pump inhibitors (PPI) for all infants with OA for the first year of life to reduce the incidence of anastomotic stricture; Currently, just over 50% of surgeons in the UK prescribe PPI prophylactically to babies with OA [1]. Babies are then managed by surgeons and neonatologists following hospital discharge. Some studies [1,4,5] and a systematic review and meta-analysis of the evidence [6], however, indicate that infants routinely given PPI are no less likely to get a stricture. The evidence to support the use of PPI is not conclusive and stems from only a small number of low quality, observational or single centre studies [6]. Furthermore, PPI can increase the risk of gastrointestinal [1,5,7,8] and respiratory infections [3,7], raising concerns about giving medication to infants that has no benefit.

A randomised clinical trial is needed to answer the question 'In infants born with oesophageal atresia, does the routine use of PPI compared to matched placebo impact the incidence or severity of anastomotic stricture?' The chances of successful trial completion are improved if the trial is deemed to be acceptable to parents and clinicians. This paper presents the findings of a mixed methods feasibility study which aimed to explore parent and practitioner views on the feasibility, acceptability and design of a proposed randomised controlled trial: Treating Oesophageal Atresia with prophylactic proton pump inhibitors to prevent STricture (TOAST).

Methods

Study Design

We conducted a mixed methods study involving interviews with parents of an infant born with OA in the last three years (June – September 2021), as well as an online survey (August – October 2021), interviews (October 2021) and focus groups (November – December 2021) with clinicians caring for infants with OA.

We used previous research [9,10] to develop participant information sheets (PIS) (see **online supplementary file 1**), protocol and online survey (see **online supplementary file 2**), whilst ongoing findings were used to develop topic guides (see **online supplementary file 3**) and as part of an iterative process. Topic guides and the survey included questions on the proposed trial design, information materials, trial acceptability, willingness to be involved/provide consent, the approach to consent and parent prioritised outcomes for the

For peer review only - http://bmjopen.bmj.com/site/about/guidelines.xhtml

proposed trial. The research was conducted in the UK between June and December 2021. The consolidated criteria for reporting qualitative research (COREQ) checklist [11] was used to aid reporting (see **online supplementary file 4**).

Patient and Public Involvement

Our parent advisory group (PAG) involved members of TOFS charity, who support infants born with OA/TOF (Tracheo-Oesophageal Fistula). The PAG met regularly before and during the study, providing valuable input into the design of research materials (including topic guide and draft reflux treatment pathway) and the conduct, progress and findings of this study. JP (TOFS Trustee) was a member of the TOAST study management team and a TOFS representative for all aspects of study development and conduct.

Recruitment and sampling procedure

Based on previous feasibility studies [9,10,12] we anticipated that we would need to interview 15-25 parents to reach information power [13], which is the point at which data addresses the study aims; sample specificity (e.g., participants' experience relevant to the study aims and sample diversity) [11]; our reflexive and interpretive approach to theory and analysis [14,15]; and sufficient quality of interview dialogue [13]. Parents were recruited via direct email from our collaborating support group TOFS, as well as via social media and study website advertising.

We aimed to recruit at least 50 clinicians to the online survey from approximately 18/25 (75%) of UK units. IY (male, Paediatric Surgeon) distributed an invitation to participate in the survey through the UK Children's Upper Gastrointestinal Surgery (CHUGS) network with a request to cascade the survey link to clinicians involved in the care of OA infants. We aimed to purposively sample clinicians who raised concerns about the proposed trial design in their survey responses and invite them to participate in a telephone interview to further explore their concerns and discuss potential ways these could be addressed to assist 'buy in'. Finally, we invited survey participants to attend an online or face-to-face focus group.

Eligibility screening and conduct

TKM (female, Research Methodologist) responded to parents' email and social media responses in sequential order, confirmed eligibility and emailed them a proposed trial Parent Information Leaflet (PIL) (see **online supplementary file 5**), draft treatment pathway for symptomatic reflux (see **online supplementary file 6**), and potential list of outcome measures (see **online supplementary file 7**) derived from a review of the literature. KW (female, Social Scientist) contacted clinicians to arrange interviews and IY sent invitations to attend a focus group. TKM and KW facilitated interviews and focus groups. Respondent validation was used to add unanticipated topics to the topic guide as interviewing and analysis progressed [16]. Findings from parent interviews and online survey were used to develop the topic guide for the clinician interviews and focus group. Interviews stopped when information power [13] was achieved and all clinicians who responded to the invite were interviewed. Parents were sent a £30 shopping voucher after their interview to thank them for their time.

Analysis

TKM led the analysis with oversight from KW. Analysis of direct questioning and indirect discussion was broadly interpretive and inductive, informed by the Theoretical Framework of

For peer review only - http://bmjopen.bmj.com/site/about/guidelines.xhtml

Acceptability (TFA), and adapted version for paediatrics [9,17]. NVivo V.12 software [18] was used to assist the organisation and coding of data. TKM and KW met regularly to discuss interpretation and develop the coding framework. Outcome measures prioritised as being most important were given a score of three, second most important a score of two, and third most important a score of one. Outcomes were then ranked. Quantitative data were entered into Microsoft Excel [19]. Descriptive statistics are presented with frequencies and percentages. Synthesis of qualitative and quantitative data for mapping findings to the TFA drew on the constant comparative method [20,21].

Results

Sample

A total of 39 parents registered interest and were screened. Three parents were deemed ineligible, three booked interviews but cancelled due to their child's hospital readmission and 15 parents did not respond to initial contact. Information power was reached at 18 parent interviews (representing 17 children), which took place via telephone (n=15) or online (n=3), lasting between 40 and 92.5 minutes, median 65 minutes (see **Table 1** for parent and child characteristics). Nine parents were recruited through TOFS, three from social media (Facebook) and six could not recollect whether TOFS email or Facebook.

Table 1 Parent and child characteristics

Parent	Mother (n=13)<br>Father (n=5)
Parent age	Between 29 and 42 years (mean = 36 years; median = 36 years)
Child age	Between 4 weeks and 34 months (mean = 14.1 months old; median = 17 months)
Gestation	Term (n=14)<br>Premature (n=3) 31^{+0} weeks, 33^{+0} weeks and 33^{+3} weeks
Country of residence	England (n=15)<br>Scotland (n=3)
Ethnic group	White British (n=15)<br>White Scottish (n=1)<br>White Other (n=1)<br>Indian (n=1)

Fifty-one clinicians (49 paediatric surgeons; 2 neonatologists) from 20/25 (80%) sites completed the online survey. Four of the six clinicians (paediatric surgeons) who indicated in the survey that they did not find the trial acceptable had provided their contact details and were contacted to take part in an interview. Two did not respond to contact and two surgeons from different sites took part in an online Zoom interview (lasting 23 and 27 minutes). Ten clinicians from nine different sites (36%) took part in one of two focus groups, one face-to-face (n=5 surgeons), one online via Zoom (n=4 surgeons; n=1 neonatologist). Both focus groups lasted one hour. See **Table 2** for clinician characteristics.

Table 2 Clinician characteristics

Method of data generation	No. of clinicians and role	No. of sites represented
Online survey	n=51: 49 paediatric surgeons and 2 neonatologists	20 (80%)
Interview	n=2: consultant paediatric surgeons	2 (8%)

For peer review only - http://bmjopen.bmj.com/site/about/guidelines.xhtml

Focus group	n=10<br>Focus group 1: 5 consultant paediatric surgeons.<br>Focus group 2: 2 consultant paediatric surgeons, 2 paediatric surgeons and 1 consultant neonatologist.	9 (36%)

Trial research question

Across the research methods, the majority of parents and clinicians (through survey responses) described or indicated that the proposed trial would answer an important research question and help address '*how little evidence there is*' (P10, mother, interview). Some parents spoke of their hope that the study would help future babies with OA, whilst both parents and clinicians stated the trial was needed to help standardise practice, and prevent babies from taking potentially unnecessary medication, whilst also reducing costs and burden for families and the NHS.

> *'I think it [the proposed trial] is a good thing, because I hear a lot of parents on the TOFS site and things, and they are obviously getting different medical care and their concerns about that really. I think it is something that needs to be standardised'* (P11, mother, interview).

> *'If significant difference found then potential to decrease burden on families and providers'* (C44, surgeon, survey).

> *'If your child doesn't need to be on a medication, then you don't really want them to be on it'* (P13, mother, interview).

Parent information

Interviews and focus groups involved a review of a draft trial Participant Information Leaflet (PIL). The majority of parents said that they found the proposed PIL to be clear and understandable. However, some stated it was *'quite long'* (P10, mother, interview) and '*text heavy'* (P16, father, interview), whilst acknowledging all necessary information was included. Recommended changes included adding a one-page overview of the study; highlighting the differences in treatment that are already happening; and not using acronyms.

Symptomatic reflux treatment pathway

The study team recognised the need to develop a tool to assist clinicians in making decisions about how to treat babies who had symptoms of reflux during the proposed trial. A symptomatic reflux treatment pathway was developed, which included options for non-pharmacological treatments (e.g., exclude overfeeding, keep baby upright after feeds) and time frames for re-evaluation (e.g., every two weeks). During interviews and focus groups the draft symptomatic reflux treatment pathway was described as *'helpful for parents and clinicians*' (C51, surgeon, survey). Parents' suggestions for improvement were mainly around the additional symptoms of reflux, signs of stricture, other non-pharmacological treatments that could be initiated, and accessibility of the document (see **online supplementary file 8a**). Clinicians suggested adding conditions such as tracheomalacia (C22, surgeon, survey), the timing of/whether babies have *'anti-reflux'* surgery (C27, surgeon, survey), and prioritising breastfeeding over formula feeding (C41, neonatologist, survey) (see **online supplementary file 8b)**.

For peer review only - http://bmjopen.bmj.com/site/about/guidelines.xhtml

Although the treatment pathway had been originally designed for use by clinicians during the trial, parents highlighted how it would be helpful to refer to and *'be aware of the things that are written down… just as a reminder of, for example, it says, "Are they gaining weight adequately? … Is he crying normally?"'* (P7, mother, interview). One father suggested that the pathway will *'make them [parents in the trial] feel more comfortable'* (P8, interview) about trial participation. Parents said they would be happy to follow the symptomatic reflux treatment pathway, *'so long as no child is being left to suffer'* (P13, mother, interview) and '*the health of the individual child would trump… being in the study*' (P18, father, interview).

Most clinicians (n=38/51, 74.5%) indicated in the survey that they would be happy to follow the treatment pathway (see **online supplementary file 6**); others raised concerns about the potential 4-week timeframe to PPI (n=5); the severity of reflux symptoms (n=4); and retention of participants (n=3):

> *'Slight concern that it may be difficult to get TOFOA parents to agree to … wait a further 4 weeks … if their child is symptomatic'* (C49, surgeon, survey)
>
> '*This would be fine for minor symptoms but inappropriate for severe symptoms'* (C12, surgeon, survey)
>
> *'I think if there is a clinician who wants to take someone out of it [the trial], you could use that escalation policy [symptomatic reflux treatment pathway] to do so...That's the difficult thing, I think'* (C29, surgeon, interview).

Support for Omeprazole as the intervention, but some concerns about side effects

Most clinicians (including 60.8% of survey participants) routinely administered or prescribed PPI following surgery in all babies with type C OA under their care. During interviews and focus groups some clinicians stated that they did not prescribe PPI following surgery due to a lack of evidence about stricture formation, or when patients did not have any symptoms of reflux. Side effects of PPI, such as the increased risk of infections, not knowing the long-term risks, or wanting to minimise unnecessary drug use were also reasons not to use PPI.

Nevertheless, all parents and the vast majority of clinicians, found Omeprazole acceptable as the trial intervention as it was a *'routinely used by many teams with a very safe profile'* (C35, surgeon, survey). The dose of 1mg/kg omeprazole orally once daily for one year was also described as being acceptable, although four mothers and two clinicians perceived 1mg/kg Omeprazole per day to be a low dose, and had *'slight concern that it may be difficult to get parents [of children with OA] to agree to that dose'* (C49, surgeon, survey). Some parents, however, wondered whether PPI *'actually had any effect'* (P17, father, interview) because their child *'still had a stricture'* (P12, mother, interview) even though they had taken PPI from birth, whilst one father (P1, interview) and one surgeon (C21, focus group 1) said that children were left off PPI and *'nothing happened anyway'* (C21, surgeon, focus group 1). A minority of parents and clinicians had concerns about side effects, such as *'it [omeprazole] seemed to thicken her mucus a lot, so it produced more blue episodes'* (P16, father, interview), *'a very sore tummy'* (P9, mother, interview) and *'sepsis/G.I. infections'* (C40, surgeon, survey), and were concerned about the trial length due to the long-term impacts of the medication. One surgeon said that infants should take PPI for '*6 months only to avoid side effects*' (C26, survey).

For peer review only - http://bmjopen.bmj.com/site/about/guidelines.xhtml

Treating reflux in the comparator arm and the challenge of changing practice

The use of a placebo in the comparator arm of the proposed trial was acceptable to both groups, although parents stated that babies should not be *'left to suffer'* with reflux (P13, mother, interview) and the symptomatic reflux treatment pathway should *'be implemented sensibly'* (P4, mother, interview), however, clinicians should not *'automatically assume you need'* PPI (P9, mother, interview). A minority of clinicians were concerned about a change in practice and placing babies at risk of negative outcomes if they were not given PPI in the trial, particularly if they have symptomatic reflux and tight anastomosis:

> *'Babies in the placebo group are exposed to a high risk of complications… It is not safe to have a baby post-TOF without PPI'* (C9, surgeon, survey).

> *'I would struggle to join a clinical trial where I know that there is a randomisation of my symptoms who were not using PPIs… I was taught the importance of the PPIs [during my career] and I think it make sense to use PPIs in this condition [OA]…if you do any repair of a tissue, you don't want to spill acid on it'* (C9, surgeon, interview).

Inclusion and Exclusion Criteria

Most clinicians who took part in the survey and focus groups were satisfied with the proposed inclusion and exclusion criteria (see **Box 1**). Recommendations for improvement covered five key areas: 1) Include babies who require *'delayed'* or *'staged'* repairs (n=11) '*as PPI might be beneficial in those'* (C43, surgeon, survey); 2) Exclude babies with tight anastomosis because of the perceived increased risk of *'reflux'* (C23, surgeon, survey) and *'stricture formation'* (C3, surgeon, survey) (n=7); 3) Exclude pre-term babies - *'Use of PPI in preterm is not neutral, and has been shown to be associated with NEC [necrotising enterocolitis] and fungal in sepsis'* (C33, neonatologist, survey) (n=5); 4) Exclude babies with other anomalies (n=9) such as '*co-existent duodenal atresia or ARM'* (C45, surgeon, survey), *'cardiac / renal / neurological / chromosomal'* (C7, surgeon, survey), '*congenital oesophageal stenosis (COS)*' (C27, surgeon, survey), '*HIE/ major brain injury* …and *VACTERL* [vertebral defects, anal atresia, cardiac defects, tracheo-oesophageal fistula, renal anomalies, and limb abnormalities]*'* (C4, surgeon, survey); and, 5) Include but consider *'the homogeneity of the… population'* of (C27, surgeon, survey) babies who have thoracoscopic rather than open repairs (n=2).

Box 1 Proposed inclusion and exclusion criteria

Inclusion criteria	Exclusion criteria
• Infants with OA with distal tracheo-oesophageal fistula undergoing primary repair at the first operative intervention in the newborn period<br>• Written informed parental consent	• Infants undergoing staged repair or delayed primary repair or requiring emergency ligation of tracheo-oesophageal fistula with primary repair later<br>• No realistic prospect of survival

The importance of not discussing the trial on the day of surgery

Parents were then asked to consider when would be the most acceptable time to be approached about the proposed trial. Most stated that two to three days after birth would be best, as long as they have received *'the good news of [their baby having] a successful repair'* (P18, father, interview) and when their baby is *'starting to look stable'* (P4, mother, interview) and is off the ventilator. A clear message from parents was that trial discussions on the day of

For peer review only - http://bmjopen.bmj.com/site/about/guidelines.xhtml

surgery would be too overwhelming. Some suggested the trial could be discussed with parents prior to birth if OA is diagnosed antenatally. Clinicians made similar recommendations to broach the discussion within 72 hours post-surgery. The concerns of the three clinicians who did not find it acceptable to approach parents at this time were, once again, around the safety of infants with OA who do not receive PPI:

> *'I usually start PPI from the time of surgery. It is not acceptable to leave the baby without PPI for 72 hours. The baby should be randomised before the surgery'* (C9, surgeon, survey).

The use of a mobile application to assist trial retention

Parents' views were sought on the use of a mobile phone application (app), which would include reminders to administer the trial intervention when they had left hospital. Most parents thought that the app was a good idea and would be a *'massive bonus'* (P7, mother, interview) and so *'useful'* (P13, mother, interview) *'to offer with'* the trial (P18, father, interview). Seven parents felt that the app is not needed, although were not averse to having an app for the study, so long as it would not be a mandatory requirement for parents to use it.

Furthermore, when questioned about content that might be useful in an app, most made a number of suggestions such as: *'hints'* (P12, mother, interview), *'tips'* (P6, mother, interview) *'and advice … on how to [prepare and] administer'* (P13, mother, interview) the intervention; reminder notifications; symptoms tracker and a medical history page because *'the days all merge… [and] sometimes I'll be like, "Oh yes, he's been coughing." And then the surgeon will be like, "So how long has that been going on for?" And I'm like, "Oh Gosh, I don't know"'* (P3, mother, interview). Other suggestions included information about the study and the main signs of reflux and stricture and *'a guide to CPR because I know a lot of parents are very, very anxious about that'* (P4, mother, interview).

Shared views on outcomes of importance

Parents and clinicians were then asked to consider a list of potential outcomes sent prior to interview and focus groups, as well as any additional outcomes they felt should be included. Parents and clinicians suggested edits or additions to most of the predefined outcomes, as shown in **online supplementary file 9**.

Participants were asked to rank the outcomes that were most, second and third most important to be measured in the TOAST trial. After weighting, 'Severity of anastomotic stricture', 'Incidence of anastomotic stricture', 'Need for treatment of reflux' and the 'Presence of symptoms of reflux' remained the four most important outcome measures for the TOAST trial for both parents and clinicians.

Potential barriers to trial success

In the survey, 38 (38/51; 74.5%) clinicians stated they were a little (n=32) or very (n=6) concerned about the retention of babies in the trial if they have symptomatic reflux and were receiving the placebo, reflecting the concerns of some parents and clinicians interviewed:

For peer review only - http://bmjopen.bmj.com/site/about/guidelines.xhtml

> *'You might put a baby in a placebo group, but if they have reflux, they'll have to have the antacid medication, and reflux is really- well, as far as I'm aware, really common'* (P3, mother, interview).

> *'If babies become symptomatic then parents may ask to come out of trial and be assured that they're on a PPI'* (C20, surgeon, survey).

Some clinicians' concerns about retention of participants in the trial related to stricture management in babies with signs of reflux:

> *'If a patient has a particularly difficult stricture, and signs of reflux I would want to know if they are being treated or just on placebo, as at this point, I would definitely want them on a PPI'* (C15, surgeon, survey).

Other clinicians' concerns were about geographical challenges, highlighting the need for '*as many continuity sites as possible'* (C33, surgeon, survey) to be tertiary centres and *how 'some of our remote / poorer patients would struggle to travel to face-to-face follow-up'* (C4, surgeon, survey). A combination of external factors and trial setup queries were discussed including: the *'differing views of… surgical and neonatal (and other) colleagues'* (C21, surgeon, survey) about preference for use of PPI and prescribing outside of the trial; quality of intervention blinding and sourcing; staffing and research support issues, especially *'out of hours'* (C2; C30, surgeons, survey); access to training and support needs; and, reflecting the concerns of a small number of parents, the pro-medication influence of TOFS Facebook group members:

> *'It will be interesting to see what the parents have said, and what, like the TOFS group says, because I think most of the parents will be members of that group, and what they feel about reflux and how willing they would be if they go on the forum and say, "Oh, I think my kid's refluxing and he's on this trial. What should I do?" What advice they're going to be given from the parent groups because I think that would be a big factor'* (C29, surgeon, interview).

> *'It was only when I joined the TOFS Facebook group that I thought, "Oh dear there's a lot of stuff going on and a lot of complications and a lot of people talking about medication the whole time"'* (P9, mother, interview).

Overall views on trial acceptability

Towards the end of the interview, survey or focus group participants were asked to consider the overall acceptability of the trial. All eighteen parents stated that the proposed trial was acceptable; three with the proviso that their child could access PPI medication if clinically necessary and so long as *'a nice, softly-softly approach'* (P16, father, interview) was taken by an experienced and *'trusted doctor or surgeon'* (P9, mother, interview). Having trust in the opinions of health professionals about their child's involvement in the trial was mentioned (unprompted) by over half of parents.

Almost all clinicians stated that they found the proposed trial to be acceptable overall, despite the potential barriers to success described above. The views of the two clinicians who found the trial 'not acceptable' in the survey appeared to shift in favour of the trial during their

For peer review only - http://bmjopen.bmj.com/site/about/guidelines.xhtml

subsequent interview, during which the evidence which questioned the use Omeprazole was discussed and changes to the reflux treatment pathway were explained, including parent views.

Finally, our findings were considered against the adapted Theoretical Framework of Acceptability (TFA) for paediatric trials [9:9,18:522], which consists of eight component constructs (see **Table 3**).

Table 3 Adapted Theoretical Framework of Acceptability [9,18]

Construct	Definition
Affective attitude	How an individual feels about the intervention.
Burden	The perceived amount of effort that is required to participate in the intervention.
Ethicality	The extent to which the intervention has a good fit with an individual's value system
Intervention coherence	The extent to which the participant understands the intervention and how it works.
Opportunity costs	The extent to which benefits, profits, or values must be given up to engage in the intervention.
Perceived effectiveness	The extent to which the intervention is perceived likely to achieve its purpose.
Self-efficacy	The participant's confidence that they can perform the behaviour(s) required to participate in the intervention.
Trust*	The extent to which the participant (or parent/guardian) trusts those delivering the intervention to put the needs of patient before the requirements of the study

Analysis of feasibility study data indicates that five out of eight constructs of the TFA (affective attitude, burden, intervention coherence, self-efficacy and trust) for the TOAST trial were fully met for parents. Concerns of a minority related to the ethicality construct and the proposed Omeprazole dose (1mg/kg) being insufficient to treat reflux symptoms and potential side effects. The remaining constructs were largely met, or could be met, if suggestions for changes to the trial materials and protocol are addressed by the team.

Although almost all clinicians stated they found the proposed TOAST trial acceptable overall, only two out of seven constructs of the TFA (affective attitude and burden) were fully met for clinicians who completed the survey and three met (affective attitude, burden and opportunity costs) for those who took part in the focus group or interviews. As the themes presented in this paper highlight, wider issues impacted upon anticipated acceptability including: the ability to retain patients in the trial due to concerns about a potential 4-week timeframe to PPI for babies with symptomatic reflux; a change in practice; and the need to amend the inclusion criteria to make the trial more acceptable for some.

Discussion

This study provides insight into the acceptability of the proposed TOAST trial for parents and clinicians who care for infants with OA. Like other studies that highlight the value of feasibility work [10] and 'conducting pre-trial research with key stakeholders' [9:9,22] to improve recruitment and retention in clinical trials [22,23], involving parents and clinicians in this

For peer review only - http://bmjopen.bmj.com/site/about/guidelines.xhtml

feasibility study provided valuable insight into potential barriers and solutions to recruitment and retention of infants in the TOAST trial.

Overall, the majority of parents and clinicians who took part in this feasibility study supported the proposed trial as they felt it would help address an area of clinical uncertainty. Parents and clinicians ranked the same top four outcomes (‘Severity of anastomotic stricture’, ‘Incidence of anastomotic stricture’, ‘Need for treatment of reflux’ and the ‘Presence of symptoms of reflux’) as important to measure for this study. Our findings highlight the need to carefully consider how symptomatic reflux would be treated in all trial participants. Although all parents found the use, dose and duration of omeprazole as the intervention medication and placebo control acceptable, some parents whose child had experienced signs of symptomatic reflux [8] had concerns about being able to access PPI if their child was in discomfort. Parents of children who had experienced commonly reported side effects of PPI, such as infections, wind or an upset stomach [1,3,5,7,8], or a previously unreported side effects, such as thick mucus that made breathing difficult, stated they would still hypothetically consent for their child to take part in the trial even if they had a 50/50 chance of receiving PPI.

Our findings show that despite clinicians stating that they found the trial acceptable, multiple constructs in the Theoretical Framework of Acceptability were not fully met due to concerns or perceived challenges to conducting the trial. Some were external factors that they felt parents may face, such as the ability of families to travel to follow-up appointments, or the pro-medication influence of TOFS Facebook group impacting on the views of new parents of OA infants invited to participate. As also found by others [24], most other challenges raised related to changing usual individual clinical practice, and for this study, clinician equipoise and specifically a wish to access PPI when children were showing signs of reflux, in line with existing guidelines [3]. As described above, these findings echo the concerns of some parents. Refining the inclusion and exclusion criteria and developing a symptomatic reflux pathway that clinicians would find acceptable will be key to ensuring they are willing to enrol infants in their care into the trial. Whilst reviewing this pathway with parents during interviews, it became apparent that they also viewed the pathway as an important resource for parents in the trial, which may assist with participant retention. Many felt it would bring reassurance that babies ‘would not be left to suffer’ with symptoms of reflux if they took part in the TOAST trial. Parents supported the use of an ‘opt in’ mobile phone application that would send reminders to administer the trial intervention, as well as host the symptomatic reflux pathway and other related trial information, all of which may help with protocol adherence and help prevent withdrawal from the trial.

Previous research has shown the importance of identifying when is an appropriate time to discuss trial participation, as a poorly timed approach can cause additional burden for distressed families, which may also increase the likelihood that parents will decline trial participation [25,26]. Parents stated that it would not be acceptable for clinicians to broach the trial on the day of surgery as it would be too overwhelming. Timepoints before surgery, when a baby is diagnosed with OA during pregnancy, and two to three days after surgery were recommended by both parents and clinicians.

We found that the views of a minority of clinicians whose survey responses suggested they did not find the trial acceptable appeared to shift in favour of the trial during subsequent interviews. During these conversations the evidence that indicated PPIs may increase stricture

For peer review only - http://bmjopen.bmj.com/site/about/guidelines.xhtml

rates [5,6], parent views, and the proposed reflux treatment pathway were explained. This finding alone highlights content that should be included in staff training and trial resources, as well as wider findings that demonstrate parental support for the trial. Inclusion of a statement on the treatment pathway which states that if clinicians 'feel that urgent treatment is needed, clinical judgement takes precedence' is also likely to help address concerns about the potential 4-week timeframe to administer PPI and therefore make the trial seem more acceptable. However, it is also important to recognise that such a statement may also lead to cross over between trial arms, or patient withdrawal, which should be closely monitored in the pilot trial phase.

Strengths and limitations of this study

A mixed methods approach including a survey, interviews and focus groups enabled comprehensive insight into key stakeholder views, as well as the ability to explore clinician concerns that were evident in the survey in more depth through interviews. Although 39 parents registered interest in an interview, nearly half did not respond to further correspondence and three cancelled due to their child being readmitted to hospital, which highlights the challenges of engaging parents of such vulnerable children in research. Despite the difficulties experienced in arranging interviews we continued to interview parents until the point of information power and our study included parents with recent relevant experience. As the majority of parents were recruited through the TOFS support group, our sample may comprise of experienced parents with an interest in OA research and may not reflect the potential TOAST sample who will also have less awareness of PPI and treatment options for symptoms of reflux at the time the trial is discussed. The clinicians involved were overwhelmingly surgeons, with only two neonatologists taking part in the survey. However, as surgeons will predominantly be deciding which babies to approach for the TOAST trial, this is unlikely to a be significant limitation to assessing the feasibility of the trial.

Conclusions

All parents and most clinicians viewed the proposed trial as being feasible and acceptable, so long as infants can access PPI if clinically required. Our findings will inform the trial protocol for the internal pilot phase of the main trial as well as the main trial itself and site training materials to ensure the trial is family centred and to assist clinician engagement. Recruitment, retention and protocol adherence data should be closely monitored during the pilot phase to inform decisions about progression to a full trial.

Author contributions

TKM, NJH, IY, CC, PH, AK, DM, CCR, KS, RW, JP and KW were involved in the conception or design of the study; or the acquisition, analysis, or interpretation of data for the work. TKM and KW collected the data. TKM analysed and interpreted the data with oversight from KW. TKM and KW drafted the article and TKM, NJH, IY, CC, AK, EN, CCR, KS, RW, JP and KW critically revised it for important intellectual content. TKM, NJH, IY, CC, PH, AK, DM, EN, CCR, KS, RW, JP and KW gave approval of the version to be published. TKM and KW agree to be accountable for all aspects of the work in ensuring that questions related to the accuracy or integrity of any part of the work are appropriately investigated and resolved. TKM, KW, NJH and IY revised the article after peer review.

For peer review only - http://bmjopen.bmj.com/site/about/guidelines.xhtml

Competing interests' statement

National Institute for Health Research (NIHR) Health Technology Assessment (HTA) Programme grant payments to support the conduct of this study were made to the institutions of all authors (except JP). TKM and EN employment roles were funded by the NIHR HTA programme grant payment made to their institutions.

Funding statement

The TOAST feasibility study was funded by the National Institute for Health Research Health Technology Assessment Programme (project number NIHR 131136; link to Protocol: https://fundingawards.nihr.ac.uk/award/NIHR131136) and coordinated by the University of Liverpool. The views and opinions expressed herein are those of the authors and do not necessarily reflect those of the HTA Programme, NIHR, NHS or the Department of Health. The funders had no role in the collection, analysis and interpretation of data or in the decision to submit this article for publication.

Data availability statement

No data are available. The datasets generated and analysed for this study are not publicly available due to participants being from a small, specialised population, which creates ethical and privacy concerns about being too identifiable.

Acknowledgements

Special thanks are extended to TOFS parent advisory group members for their involvement in the design, conduct, progress and findings of the feasibility study and the development and conduct of the proposed trial. Gratitude is extended to Alan Downs[d] for his involvement in this study.

Supplementary material

This content has been supplied by the authors. It has not been vetted by BMJ Publishing Group Limited (BMJ) and may not have been peer-reviewed. Any opinions or recommendations discussed are solely those of the authors and are not endorsed by BMJ. BMJ disclaims all liability and responsibility arising from any reliance placed on the content. Where the content includes any translated material, BMJ does not warrant the accuracy and reliability of the translations (including, but not limited to, local regulations, clinical guidelines, terminology, drug names and drug dosages), and is not responsible for any error and/or omissions arising from translation and adaptation or otherwise.

Open access

This is an open access article distributed in accordance with the Creative Commons Attribution 4.0 International (CC BY 4.0) license, which permits others to copy, redistribute, remix, transform and build upon this work for any purpose, provided the original work is properly cited and appropriate credit given, a link to the licence is given, and indication of whether changes were made. See: https://creativecommons.org/licenses/by/4.0/.

For peer review only - http://bmjopen.bmj.com/site/about/guidelines.xhtml

Ethics approval

The TOAST feasibility study was approved by the University of Liverpool Research Ethics Committee (Reference number: 8510).

Reference list

1. Allin B, Knight M, Johnson P, Burge D. Outcomes at one-year post anastomosis from a national cohort of infants with oesophageal atresia. PLoS One. 2014;9(8). DOI: 10.1371/journal.pone.0106149.
2. Tambucci R, Angelino G, De Angelis P, Torroni F, Caldaro T, Balassone V, Chiara Contini A., Romeo E, Rea F, Faraci S, Federici di Abriola G, Dall'Oglio L. Anastomotic Strictures after Esophageal Atresia Repair: Incidence, Investigations, and Management, Including Treatment of Refractory and Recurrent Strictures. In: Krishnan U, Faure C, editors. Update on Oesophageal Atresia-Tracheoesophageal Fistula. Frontiers in Pediatrics; 2017. p. 48–61. DOI:10.3389/978-2-88945-304-7
3. Krishnan U, Mousa H, Dall'Oglio L, Homaira N, Rosen R, Faure C, Gottrand F. ESPGHAN-NASPGHAN guidelines for the evaluation and treatment of gastrointestinal and nutritional complications in children with esophageal atresia-tracheoesophageal fistula. J Pediatr Gastroenterol Nutr. 2016;63(5):550–70. DOI: 10.1097/MPG.0000000000001401
4. Hagander L, Muszynska C, Arnbjornsson E, Sandgren K. Prophylactic treatment with proton pump inhibitors in children operated on for oesophageal atresia. Eur J Pediatr Surg. 2012;22(2):139–42. DOI: 10.1055/s-0032-1308698
5. Jones CE, Smyth R, Drewett M, Burge DM, Hall NJ. Association Between Administration of Antacid Medication and Anastomotic Stricture Formation After Repair of Esophageal Atresia. J Surg Res. 2020;254:334–9. DOI:10.1016/j.jss.2020.05.004.
6. Miyake H, Chen Y, Hock A, Seo S, Koike Y, Pierro A. Are prophylactic anti-reflux medications effective after esophageal atresia repair? Systematic review and meta-analysis. Pediatr Surg Int. 2018;34(5):491–7. DOI:10.1007/s00383-018-4242-4.
7. Santana RNS, Santos VS, Ribeiro-Júnior RF, Freire MS, Menezes MAS, Cipolotti R, Gurgel RQ. Use of ranitidine is associated with infections in newborns hospitalized in a neonatal intensive care unit: A cohort study. BMC Infect Dis. 2017;17(1):1–6. DOI: 10.1186/s12879-017-2482-x
8. National Institute for Health and Care Excellence (NICE). GORD in children: Omeprazole. GORD in children: Omeprazole. 2020 [cited 2022 May 19]. Available from: https://cks.nice.org.uk/topics/gord-in-children/prescribing-information/omeprazole/
9. Deja E, Peters MJ, Khan I, Mouncey PR, Agbeko R, Fenn B, Watkins J, Ramnarayan P, Tibby SM, Thorburn K, Tume LN, Rowan KM, Woolfall K. Establishing and augmenting views on the acceptability of a paediatric critical care randomised controlled trial (the FEVER trial): A mixed methods study. BMJ Open. 2021;11(3):1–11. DOI: 10.1136/bmjopen-2020-041952
10. O'Hara CB, Canter RR, Mouncey PR, Carter A, Jones N, Nadel S, Peters MJ, Lyttle MD, Harrison DA, Rowan KM, Inwald D, Woolfall K. A qualitative feasibility study to inform a randomised controlled trial of fluid bolus therapy in septic shock. Arch Dis Child. 2018;103(1):28–32. DOI: 10.1136/archdischild-2016-312515
11. Tong A, Sainsbury P, Craig J. Consolidated criteria for reporting qualitative research (COREQ): A 32-item checklist for interviews and focus groups. Int J Qual Heal Care. 2007;19(6):349–57. DOI: 10.1093/intqhc/mzm042
12. Deja E, Roper L, Tume LN, Dorling J, Gale C, Arch B, Latten L, Pathan N, Eccleson H, Hickey H, Preston J, Beissel A, Andrzejewska I, Valla FV, Woolfall K. Can they stomach it? Parent and practitioner acceptability of a trial comparing gastric residual volume measurement versus no gastric residual volume in UK NNU and PICUs: a feasibility

For peer review only - http://bmjopen.bmj.com/site/about/guidelines.xhtml

study. Pilot Feasibility Stud. 2021;7(1):1–9. DOI: 10.1186/s40814-021-00784-5
13. Malterud K, Siersma VD, Guassora AD. Sample Size in Qualitative Interview Studies: Guided by Information Power. Qual Health Res. 2016;26(13):1753–60. DOI: 10.1177/1049732315617444
14. Braun V, Clarke V. Reflecting on reflexive thematic analysis. Qual Res Sport Exerc Heal. 2019;11(4):589–97. DOI:10.1080/2159676X.2019.1628806.
15. Angen MJ. Evaluating interpretive inquiry: Reviewing the validity debate and opening the dialogue. Qual Health Res. 2000;10(3):378–95. DOI: 10.1177/104973230001000308
16. Mays N, Pope C. Qualitative research in health care: Assessing quality in qualitative research. Br Med J. 2000;320(7226):50–2. DOI: 10.1136/bmj.320.7226.50
17. Sekhon M, Cartwright M, Francis JJ. Acceptability of health care interventions: A theoretical framework and proposed research agenda. Br J Health Psychol. 2018;23(3):519–31. DOI: 10.1111/bjhp.12295
18. QSR International. What is NVivo? Introducing the NVivo Suite. 2022 [cited 2022 May 19]. Available from: About NVivo (NVivo 11 for Windows) (qsrinternational.com)
19. Microsoft Corporation. Microsoft ® Excel ® 2019 MSO. Microsoft Office Professional Plus 2019.
20. Corbin J, Strauss A. Basics of qualitative research: Techniques and procedures for developing grounded theory. Fourth. London: SAGE Publications Ltd.; 2015. ISBN10 1412997461
21. Glaser BG. The Constant Comparative Method of Qualitative Analysis. Soc Probl. 1965;12(4):436–45. Available from: https://www.jstor.org/stable/798843
22. Tudur Smith C, Hickey H, Clarke M, Blazeby J, Williamson P. The trials methodological research agenda: Results from a priority setting exercise. Trials. 2014;15(32). DOI: 10.1186/1745-6215-15-32
23. Newlands R, Duncan E, Treweek SD, Elliott J, Presseau J, Bower P, Maclennan G, Ogden M, Witham M, Young B, Gillies K. The Development of Theory-Informed Participant-Centred Interventions to Maximise Participant Retention in Randomised Controlled Trials. Trials [Internet]. 2022;23(268):1–16. DOI: 10.21203/rs.3.rs-917451/v1.
24. Gupta DM, Boland RJ, Aron DC. The physician's experience of changing clinical practice: A struggle to unlearn. Implement Sci. 2017;12(1):1–11. DOI: 10.1186/s13012-017-0555-2
25. Woolfall K, Young B, Frith L, Appleton R, Iyer A, Messahel S, Hickey H, Gamble C. Doing challenging research studies in a patient-centred way: A qualitative study to inform a randomised controlled trial in the paediatric emergency care setting. BMJ Open. 2014;4(e005045). DOI: 10.1136/bmjopen-2014-005045
26. Woolfall K, Frith L, Gamble C, Gilbert R, Mok Q, Young B. How parents and practitioners experience research without prior consent (deferred consent) for emergency research involving children with life threatening conditions: A mixed method study. BMJ Open. 2015;5(9):1–14. DOI: 10.1136/bmjopen-2015-008522

For peer review only - http://bmjopen.bmj.com/site/about/guidelines.xhtml

Treating Oesophageal Atresia to Prevent Stricture

TOAST feasibility study

Parent/Legal Representative Information Sheet

We invite you to take part in a research study

Following repair of oesophageal atresia some babies develop a narrowing at the site of the repair in the oesophagus called a stricture. Reflux is also very common after repair of oesophageal atresia and it is believed that reflux of acid into the oesophagus may make a stricture more likely or more severe.

Antacid medication is sometimes used to supress the acid produced by the stomach and help reduce the risk of strictures. Despite this treatment being used in about half of babies with oesophageal atresia, the evidence for using this medication to reduce the chance of getting a stricture is weak. We don't really know if it works. But we would like to find out because if it does then all babies with oesophageal atresia can be given this treatment and if it does not then we can stop asking parents to give medicine to their baby that has no benefit.

We want to answer the question, "Should babies born with oesophageal atresia all be treated routinely with antacid medication to reduce strictures?" To do this we are planning a clinical trial, which will be the first of its kind, to help inform the future treatment of babies with oesophageal atresia.

- To help find out what parents think about the trial and help to design it, we would like to interview about 25 parents/legal representatives with experience of infants born with oesophageal atresia within the last three years, including those with or without stricture and those who did or did not receive routine antacid medication
- By speaking to parents/ legal representatives we hope to find out whether the trial is acceptable and, if so, how it should be done.
- The interview should take about 30-45 minutes and can be done over the telephone or online (e.g. Zoom). Should you be interviewed, you will receive a £30 Amazon voucher to thank you for your time.

How to contact us

If you have any questions and/or would like to take part in an interview, please contact: Dr Tracy Mitchell Telephone: 07379 105134 Email: tracy.mitchell@liverpool.ac.uk Further information can be found on our website: ***https://www.npeu.ox.ac.uk/toast/parents***

Why are we doing this study?

Babies may suffer reflux after surgery where the acid content of the stomach comes back into the oesophagus and can cause regurgitation of feeds and/or damage to the oesophagus. Sometimes a narrowing of the tube (called a stricture) where the oesophagus was rebuilt may be caused or made worse by reflux. Some surgeons who look after babies with oesophageal atresia use a medication to suppress the acid produced by the stomach even if there are no symptoms of reflux. A major reason for this is to reduce the risk of strictures forming. Despite this being an apparently popular option (about half of babies with oesophageal atresia are treated with this medication) the evidence for using this medication is weak. In fact, some studies of babies with oesophageal atresia have actually found that strictures are more common in babies treated with acid suppression than in those who were not. In addition, there is some suggestion that taking the medicine can increase the chance of certain types of infection. Until we conduct a clinical trial we don't really know the best way to treat babies with oesophageal atresia.

For peer review only - http://bmjopen.bmj.com/site/about/guidelines.xhtml

We want to answer the question, "Should babies born with oesophageal atresia all be treated routinely with antacid medication to reduce strictures?" To do this we are planning a two-phased study. Firstly, we will assess the feasibility of the study. We will work with surgeons, other doctors looking after babies with oesophageal atresia, families of such babies and others who are involved in their care to explore what is important to them and whether they would be willing to take part in a trial where we test our question. Secondly, the findings from this will then be used to design a trial where babies with oesophageal atresia are allocated at random to either being given acid suppressing medicine or a placebo (an inactive substance that looks the same as the medicine). This is called a randomised controlled trial and is considered the "Gold Standard" in terms of answering clinical questions like this.

Who can take part?

Parents/legal representatives with experience of a baby with oesophageal atresia, including those with or without stricture and those who did or did not receive routine antacid medication in the last three years.

How can I take part?

If you would like to take part in an interview, please contact Tracy Mitchell or Kerry Woolfall by email or telephone (contact details are on the first page). If two parents/guardians in the same family wish to take part then the interviews will be conducted separately, one at a time.

Before the interview, we will send you an information sheet (like this one) which will be used to invite parents/guardians to take part in the TOAST clinical trial.

During the interview, Tracy will ask you what you think about the information sheet and what improvements you think we could make. We would also appreciate your views on the best way to speak to parents about this research, the design of the study (such as how and when medication is given to babies with reflux), our approach to seeking consent and your views on the parent / family centred things that we plan to observe as outcomes of the trial. With your permission we would like to record the interviews for analysis purposes but all names and identifying information will be removed. Interviews will be digitally recorded which will then be written out by the researcher or a transcription service. After the interview, we will send you a £30 Amazon voucher to thank you for your time.

The views of parents with experience infants born with oesophageal atresia are very important to make sure we design this study in a way that is acceptable to parents/guardians and ensure that they understand the study information given to them in such stressful circumstances.

Taking part is completely optional and you can change your mind about being part of the study at any time by contacting the study team.

If you have any questions before deciding to take part, please do not hesitate to contact us. The study results will be made available on the study website when the study is finished.

Are there any risks in taking part?

This is a low risk study. The majority of questions will be about the proposed clinical trial. However, at the beginning of the interview we will be inviting you to discuss your child's experiences, such as when was your child's oesophageal atresia first identified or diagnosed. And did your child experience a narrowing of their oesophagus following this surgery? Such personal questions about your child's experience may be upsetting for some. If you would rather not answer such questions, please let the interviewer know. You can decide not to answer a question at any time. The interview can also be paused or stopped at any time you wish. Details of additional support such as the TOFS (Tracheo-Oesophageal Fistula Support) group for parents are provided on the last page of this information sheet.

For peer review only - http://bmjopen.bmj.com/site/about/guidelines.xhtml

Who is involved in this study?

The study is being funded by the National Institute for Health Research (NIHR) which is the research arm of the NHS. Mr Nigel Hall (University of Southampton) and Mr Iain Yardley (Evelina Children's Hospital) are the TOAST Study Chief Investigators. At a later stage, it is planned that the the National Perinatal Epidemiology Unit, Clinical Trials Unit (NPEU CTU) at the University of Oxford (which has been central to previous UK-wide surveys of babies with Oesophageal Atresia) will conduct the trial. Dr Kerry Woolfall (University of Liverpool) is leading this element of the study. The TOAST research team are qualified to do this study because they have all the specialties and skills needed. Members of team have a lot of experience in caring for children with oesophageal atresia and are very active in health research. Parents of children oesophageal atresia and TOFS have been involved in the development of this study.

How will my data be used and what happens if I want to stop taking part?

The University of Liverpool is the sponsor for this study based in the United Kingdom. We will be using information you provide us with during interviews in order to undertake this study and will act as the data controller for this study. This means that we are responsible for looking after your information and using it properly. The University of Liverpool will keep identifiable information about you for 10 years after the study has finished.

Your rights to access, change or move your information are limited, as we need to manage your information in specific ways in order for the research to be reliable and accurate. Participation in the study is voluntary and you are free to withdraw without explanation up until your data has been anonymised (approximately a week after the interview). All identifiable information removed and saved anonymously.

The University processes personal data as part of its research and teaching activities in accordance with the lawful basis of 'public task', and in accordance with the University's purpose of "advancing education, learning and research for the public benefit." Under UK data protection legislation, the University acts as the Data Controller for personal data collected as part of the University's research. Dr Kerry Woolfall, acts as the Data Processor for this study, and any queries relating to the handling of your personal data can be sent to Dr Kerry Woolfall, University of Liverpool (Tel: 0151 794 4634).

Further information on how your data will be used can be found in the table below.

How will my data be collected?	Digital recording of interviews.
How will my data be stored?	Password protected data files.
How long will my data be stored for?	10 years
What measures are in place to protect the security and confidentiality of my data?	Any identifiable information will be taken out of the interviews when written out and each participant will be assigned a number. Consent forms will be securely stored.
Will my data be anonymised?	All identifiable information will be removed, and a number assigned to each participant.
How will my data be used?	Findings will be written up for the funders NIHR. Publication will be sought with peer reviewed journals.
Who will have access to my data?	Tracy Mitchell and Kerry Woolfall
Will my data be archived for use in other research projects in the future?	No
How will my data be destroyed?	Shredded or deleted after 10 years

For peer review only - http://bmjopen.bmj.com/site/about/guidelines.xhtml

What if there is a problem?

If you are unhappy, or if there is a problem, please feel free to let us know by contacting **Kerry Woolfall, Telephone: 0151 794 4634** and we will try to help. If you remain unhappy or have a complaint which you feel you cannot come to us with then you should contact the Research Ethics and Integrity Office at ***ethics@liv.ac.uk***. When contacting the Research Ethics and Integrity Office, please provide details of the name or description of the study (so that it can be identified), the researcher(s) involved, and the details of the complaint you wish to make. The University strives to maintain the highest standards of rigour in the processing of your data. However, if you have any concerns about the way in which the University processes your personal data, it is important that you are aware of your right to lodge a complaint with the Information Commissioner's Office by calling 0303 123 1113. For NHS service advice or support please contact: Patient Advice and Liaison Services (PALS) services. Go to ***www.nhs.uk*** to find your local PALS contact details. There is also the TOFS (Tracheo-Oesophageal Fistula Support) support group for parents:

www.tofs.org.uk/about-us.aspx

Telephone +44 (0)115 961 3092

Email: ***info@tofs.org.uk***

Who has reviewed the study?

The study has been reviewed by the University of Liverpool Research Ethics Committee (REC Ref. 8510) who have agreed that the study is being conducted in a correct and appropriate manner.

Thank you for your time.
We are very grateful that you are considering taking part in this study.

For peer review only

TOAST Study Team
NPEU Clinical Trials Unit, University of Oxford,
Old Road Campus, Headington, Oxford, OX3 7LF.
01865 617919
toast@npeu.ox.ac.uk
www.npeu.ox.ac.uk/toast

TOAST is funded by the National Institute for Health Research (NIHR) Health Technology Assessment programme (project reference 131136). The views expressed are those of the author(s) and not necessarily those of the NIHR or the Department of Health and Social Care.

For peer review only - http://bmjopen.bmj.com/site/about/guidelines.xhtml

TOAST Practitioner Questionnaire

Thank you for agreeing to help us with a study assessing the feasibility of a proposed RCT into the use of proton pump inhibitor (PPI) medication (Omeprazole) to reduce anastomotic stricture formation following oesophageal atresia repair. We would like to explore your attitudes and opinions about the study concept, the trial design and ways in which we can make the project more likely to succeed. This questionnaire, which will take approximately 15 minutes to complete, will explore your views on the proposed trial design, including questions to assess your views on trial acceptability, the inclusion/exclusion criteria, and your willingness to be involved in the TOAST trial.

Your responses are important to us and will help to refine the trial protocol. Please note that by completing this survey you are giving permission for your responses to be included in the NIHR funded TOAST study. All information will be held by the University of Liverpool, anonymised and stored securely in compliance with UK GDPR.

If you have any questions, please email Dr Tracy Mitchell: Tracy.Mitchell@liverpool.ac.uk or Dr Kerry Woolfall: woolfall@liverpool.ac.uk. Further information about the study can be found here: https://www.npeu.ox.ac.uk/toast/clinicians

It is recommended that you download the 'Treatment plan for treating babies with symptoms of reflux' flowchart before commencing this questionnaire to be able to see the text more clearly, especially if completing this questionnaire on your mobile phone: https://www.npeu.ox.ac.uk/assets/downloads/toast/pathway.pdf

Once you have read and understood each statement, please click on the box to indicate consent.

* Required

1. I confirm that I have read and understood the information sheet. I have had the opportunity to consider the information, ask questions and have these answered satisfactorily *

Check all that apply.

☐ Yes

2. I agree to participate in the questionnaire *

Check all that apply.

☐ Yes

3. I understand that my responses will remain confidential and will not be used for any other purpose *

Check all that apply.

- [ ] Yes

4. I understand that I am free to withdraw from the study at any time without reason, and if so, all of my information will be disposed of *

Check all that apply.

- [ ] Yes

5. I understand that the results from the study may be published in an academic journal and that it will be kept anonymous *

Check all that apply.

- [ ] Yes

6. I agree to my data being stored on a computer at University of Liverpool for 10 years in line with the standard university procedure *

Check all that apply.

- [ ] Yes

Please read this outline of the proposed Treating Oesophageal Atresia to Prevent Stricture (TOAST) trial before completing the questionnaire

As you know, babies born with oesophageal atresia (OA) may have gastro-oesophageal reflux after surgery to repair their oesophagus. The link between reflux and the development of anastomotic stricture is contentious. Some surgeons who look after babies with oesophageal atresia use proton pump inhibitor (PPI) medication to suppress the acid produced by the stomach even if there are no symptoms of reflux. A major reason for this is to reduce the risk of strictures forming. Despite this being an apparently popular option (about half of babies with oesophageal atresia are treated with PPI medication) the evidence for using this medication is weak. In fact, some studies of babies with oesophageal atresia have actually found that strictures are more common in babies treated with PPI medication than in those who were not. In addition, there is some suggestion that taking the PPI medication can increase the chance of certain types of infection.

The primary objective of the Treating Oesophageal Atresia to Prevent Stricture (TOAST) study is to address the research question ‘In babies born with oesophageal atresia does routine use of PPI medication reduce the severity or incidence of anastomotic stricture compared to placebo?’

To do this we aim to perform a multicentre double blinded randomised placebo-controlled trial (with integral internal pilot and health economic evaluation) of babies with type C oesophageal atresia (i.e., only those with distal fistula) in which half will receive PPI from the time of repair until 1 year of age and the other half will receive placebo. The primary outcome of the TOAST study is to assess the severity of anastomotic stricture through the number of dilatations performed within the first year of life, and we intend to measure a range of other relevant and meaningful outcomes for up to two years. We have selected type C only so as to have a reasonably homogenous population and because this represents the majority of babies born with OA.

The research will take place in specialist U.K. neonatal surgical units and in participant homes; it is expected that approximately 12-15 units will take part. An 18-month internal pilot phase incorporates “stop-go” criteria to evaluate feasibility of recruitment and other trial processes. The trial aims to recruit 211 infants in 90 months from the recruitment start date, equating to around 3-4 infants per month across all participating sites. Eligible infants will be recruited early in life, around the time of their surgical repair, so that the trial intervention can commence as soon as possible after surgical repair. Where a diagnosis of oesophageal atresia is made antenatally, parents may be approached regarding the trial before birth. Eligible infants will be identified by the clinical care team and recruited by appropriately trained, delegated individuals. These infants will be randomised to receive PPI medication or a placebo, daily, until one year of age.

Trial data collection will be from trial entry until 24 months of age, including screening, consent, randomisation and follow-up. Outcome information will be collected by case report forms (CRFs), paper and electronic, with clinical data collection from medical records at hospital sites and parent reported outcomes. Participant compliance with administering PPI medication or placebo will be performed via a parent report App. The App may also be used for communication to parents about data collection, reportable safety events and other trial communications. Data will be collected when the child is 3, 6, 9, 12, 18, and 24 months of age on primary care contacts, out of pocket expenses, time away from work, and parental health related quality of life using the EuroQol EQ-5D-5L questionnaire. Children’s health related quality of life using the parent completed PedsQL questionnaire will be collected at 24 months.

We have designed this trial to be as pragmatic as possible, so that data are recorded during routine hospital visits as much as possible and there is little interference with your standard follow-up pathways. There key points that are important to the successful running of the trial are as follows:

- The trial will be blinded so that none of surgeons, nurses, nor parents will know which treatment is being given.
- The dose of PPI we plan to use will be 1mg/kg/day. During the trial the use

For peer review only - http://bmjopen.bmj.com/site/about/guidelines.xhtml

of other gastric acid suppression medication will generally not be allowed (e.g., other PPI or ranitidine) since we wish to maintain separation between the trial groups. To allow for symptoms of reflux in babies to be treated we have designed a pathway for escalating treatment of reflux symptoms for use in this trial. This will also prevent inadvertent overdosing with PPI.

• During the trial, we will be discouraging the use of 'routine' dilatations / calibrations, reserving these only for when an infant is symptomatic. However, we will be leaving the definition of symptomatic to clinician judgement.

The schedule of events for the proposed trial are summarised in the flow chart below.

Flow chart: Treating Oesophageal Atresia to prevent STricture (TOAST)

Inclusion criteria

- Infants with oesophageal atresia with distal trache-oesophageal fistula undergoing primary repair at the first operative intervention in the newborn period
- Written informed parental consent

Exclusion criteria

- Infants undergoing staged repair or delayed primary repair or requiring emergency ligation of trache-oesophageal fistula with primary repair later
- No realistic prospect of survival

Randomisation (1:1 allocation ratio)

Routine anti-reflux medication

Esomeprazole 0.5mg/kg whilst infants are nil by mouth, followed by omeprazole 1mg/kg

Given once daily from trial entry for one year

OR

Placebo

Given once daily from trial entry for one year

Primary Outcome

Severity of anastomotic stricture
(defined as number of dilatations performed to one year of age)

Secondary Outcomes

Measured at 3, 6, 9, 12, 18 and 24 months after trial entry

- Incidence of anastomotic stricture (0 vs 1 or more dilatations)
- Other complications directly related to oesophageal atresia
- Adverse events
- Presence of symptoms of gastro-oesophageal reflux
- Need for treatment of reflux
- Growth
- Respiratory symptoms
- Oral intake
- Episodes of infection
- Mortality
- Maternal Health Related Quality of Life (EQ-5D-5L)
- Child Health Related Quality of Life (PedsQL; parent reported, collected at age 24 months)
- Healthcare and societal costs
- Incremental cost per dilation prevented
- Other meaningful outcomes proposed during the feasibility phase

Longer-term follow up

Permission will be requested to access trial participants' healthcare and educational records for the measurement of longer-term outcomes using routinely collected data. This activity would be as part of a separately funded study.

TOAST flow chart 280721

Part 1 Background Information

7. Are you actively involved in the pre/post-surgical care of children with oesophageal atresia (OA) in the United Kingdom? *

Mark only one oval.

- Yes *Skip to question 8*
- No *Skip to section 4 (Thank you for your interest in the TOAST study. Unfortunately, this study is only involving those actively involved in the pre or post surgical care of children with oesophageal atresia (OA) in the United Kingdom.)*

Thank you for your interest in the TOAST study. Unfortunately, this study is only involving those actively involved in the pre or post surgical care of children with oesophageal atresia (OA) in the United Kingdom.

8. Which UK hospital do you work at? *

____

9. What is your job role? *

Mark only one oval.

- Surgeon *Skip to question 11*
- Neonatologist *Skip to question 11*
- Neonatal surgical specialist nurse *Skip to question 11*
- Other *Skip to question 10*

10. Please type your job role here *

____

11. Approximately how many babies with oesophageal atresia do you treat/are under your own individual care each year? *

12. Do you have any experience of recruitment to clinical trials? *

Mark only one oval.

- Yes *Skip to question 13*
- No *Skip to question 14*

13. How many years' experience do you have in recruiting to clinical trials? *

14. Do you routinely administer or prescribe prophylactic proton pump inhibitors following surgery in ALL babies with type C oesophageal atresia under your care? *

Mark only one oval.

- Yes *Skip to question 15*
- No *Skip to question 16*

15. Please can you explain your reasons for prescribing prophylactic PPIs following surgery? *

Skip to question 17

16. Please can you explain your reasons for not prescribing prophylactic PPIs following surgery? *

Part 2: Inclusion and exclusion criteria

The primary objective of the Treating Oesophageal Atresia to Prevent Stricture (TOAST) study is to address the research question 'In babies born with oesophageal atresia does routine use of PPI medication reduce the severity or incidence of anastomotic stricture compared to placebo?' TOAST is a multicentre double blinded randomised placebo-controlled trial of babies with type C oesophageal atresia (i.e., only those with distal fistula) in which half will receive PPI from the time of repair until 1 year of age and the other half will receive placebo.

Our proposed inclusion criteria are:

- Infants with OA with distal tracheoesophageal fistula (TOF) undergoing primary repair at the first operative intervention.
- Written parental informed consent

17. How satisfied are you with these inclusion criteria? *

Mark only one oval.

- Very satisfied
- Satisfied
- Neutral
- Not satisfied
- Not at all satisfied

18. Do you have any comments or suggested changes for proposed inclusion criteria?

For peer review only - http://bmjopen.bmj.com/site/about/guidelines.xhtml

Our proposed exclusion criteria are:

- Infants undergoing staged repair, delayed primary repair or requiring emergency ligation of tracheoesophageal atresia with primary repair later.
- Infants with no realistic chance of survival beyond the new-born period.

19. How satisfied are you with these exclusion criteria? *

Mark only one oval.

- Very satisfied
- Satisfied
- Neutral
- Not satisfied
- Not at all satisfied

20. Do you have any comments or suggested changes for the proposed exclusion criteria?

Part 3: Trial question and acceptability

21. Do you think the proposed trial is addressing an important research question? *

Mark only one oval.

- Yes *Skip to question 22*
- No *Skip to question 23*

22. Please explain why you think that the proposed trial is addressing an important research question? *

Skip to question 24

23. Please explain why you think that the proposed trial is not addressing an important research question *

The proposed intervention is esomeprazole (0.5mg/kg intravenously) once daily whilst infants are nil by mouth followed by omeprazole (1mg/kg orally) once daily until one year of age.

24. How acceptable do you think the use of this PPI medication is in the intervention arm of the TOAST trial? *

Mark only one oval.

- Very acceptable
- Acceptable
- Neutral
- Not acceptable
- Not at all acceptable

25. How acceptable do you think the dose of 1mg/kg of omeprazole orally once daily is? *

Mark only one oval.

- Very acceptable
- Acceptable
- Neutral
- Not acceptable
- Not at all acceptable

26. How acceptable do you think the duration of one year for intervention delivery is? *

Mark only one oval.

- Very acceptable
- Acceptable
- Neutral
- Not acceptable
- Not at all acceptable

27. How acceptable would you find administering the PPI medication to babies in your care? *

Mark only one oval.

- Very acceptable
- Acceptable
- Neutral
- Not acceptable
- Not at all acceptable

28. Please elaborate on your answers for the proposed intervention medication

The comparator to esomeprazole/omeprazole is a matched placebo (e.g., saline/cellulose/water solution with no active ingredient) given intravenously once daily whilst infants are nil by mouth followed by oral administration, once daily, until one year of age.

29. How acceptable do you think the use of a placebo is in the comparator arm of the TOAST trial? *

Mark only one oval.

- Very acceptable
- Acceptable
- Neutral
- Not acceptable
- Not at all acceptable

30. How acceptable would you find administering a placebo to babies in your care? *

Mark only one oval.

- Very acceptable
- Acceptable
- Neutral
- Not acceptable
- Not at all acceptable

31. Please elaborate on your answers for the proposed placebo

32. Do you think the TOAST trial is practically possible to conduct? *

Mark only one oval.

- Yes
- No

33. How acceptable do you think it is to conduct TOAST Trial? *

Mark only one oval.

- Very acceptable
- Acceptable
- Neutral
- Not acceptable
- Not at all acceptable

34. How acceptable would you find randomising babies with OA under your care into the TOAST trial? *

Mark only one oval.

- Very acceptable
- Acceptable
- Neutral
- Not acceptable
- Not at all acceptable

35. Please elaborate on any of your responses above

36. How acceptable do you feel it is to give PPI medication to half the babies who take part in this study? *

Mark only one oval.

- Very acceptable
- Acceptable
- Neutral
- Not acceptable
- Not at all acceptable

37. How acceptable do you think it is to NOT give PPI medication to half the babies in this study? *

Mark only one oval.

- Very acceptable
- Acceptable
- Neutral
- Not acceptable
- Not at all acceptable

38. Please elaborate on your answers about the acceptability of giving/not giving PPI medication to half the babies in this study

____
____
____
____
____

Treatment plan for babies with symptoms of reflux

We have developed an escalating treatment plan (please see below, or if you did not download a copy before starting this questionnaire, you can this link: https://tinyurl.com/38n4wjtv) for babies with symptoms of reflux drawing on the best available evidence for the management of reflux. Babies with reflux symptoms would first be managed with changing feed frequencies and positioning advice escalating to feed thickener (if formula feed) or Gaviscon (if breast fed) and only allowed to have additional medication (e.g., omeprazole) if these fail and symptoms are felt to be having a significant impact.

TOAST

Evidence - and guideline - based rational decision tree for treating possible reflux symptoms in postoperative OA/TOF

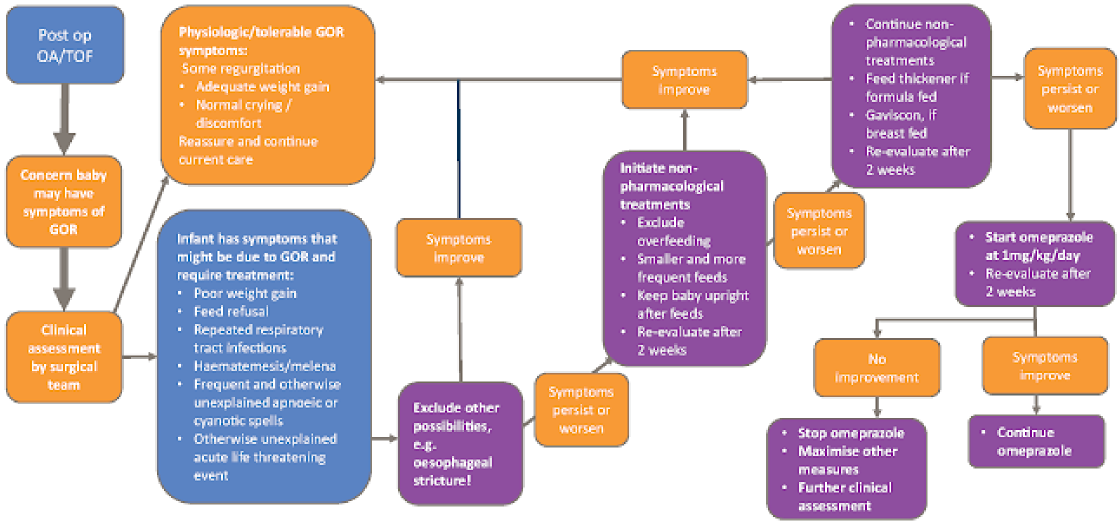

N.B. If you feel that urgent treatment is needed, clinical judgement takes precedence over the above.

39. How acceptable do you find this escalating treatment plan for babies under your care who have symptoms of reflux in the trial? *

Mark only one oval.

- Very acceptable
- Acceptable
- Neutral
- Not acceptable
- Not at all acceptable

40. Would you be happy to follow this escalating treatment plan for babies under your care who have symptoms of reflux in the trial? *

Mark only one oval.

- Yes
- No

41. Please elaborate

____
____
____
____
____

42. Does the pathway to managing reflux for babies in the trial address any concerns or raise any concerns for you? *

Mark only one oval.

- Yes
- No

43. Please elaborate and include whether anything was unclear or missing from the treatment pathway flow chart, or any changes that you would like to suggest.

Part 4: Trial outcomes and follow up

As we have discussed, in the TOAST study we want to find out if routinely giving babies PPI suppression medication after repair of oesophageal atresia will reduce the incidence or severity of oesophageal stricture. To do this we will collect information on outcomes such as those listed below:

TOAST TRIAL: OUTCOMES

Primary Outcome

1. Severity of anastomotic stricture

• *The number of dilations performed within one year of trial entry (Measured at 3, 6, 9, 12 months).*

Secondary Outcomes (Measured up to the first two years of life)

2. Incidence of anastomotic stricture

• *Whether the baby has any dilatation or none in the first year / 2 years of their life.*

3. All-cause mortality (up to 12 and 24 months)

• *Whether the baby survived to a certain time point (usually time point at months/years) or to a specific event (e.g., hospital discharge).*

4. Number of complications directly related to oesophageal atresia post-surgery

• *Anything that is directly related to oesophageal atresia or its repair, for instance anastomotic leak or recurrent fistula.*

5. Adverse events

• *A general term used to describe things that don't go as planned but aren't included in other outcomes.*

6. Presence of symptoms of gastro-oesophageal reflux

• *Symptoms that may be due to reflux and reported by parents (e.g., using a reflux symptom score, measured every three months).*

7. Treatment of reflux symptoms

• *Whether the baby is given any sort of medication to treat reflux symptoms (e.g., feed thickener, Gaviscon, Omeprazole).*

8. Weight (standard deviation score)

• *To monitor the baby's growth during the first year of their life (recorded by weight, length and head circumference at certain time points).*

9. Respiratory symptoms

• *Presence of respiratory symptoms such as infections, chronic cough (but not the usual TOF cough) or any other symptoms that a doctor felt required investigation or treatment.*

10. Oral feed intake

• *How the baby is progressing with advancing from milk feeds onto solids at 12 and 24-months using a validated measure (e.g., IDDSI score).*

11. Number of courses of antibiotics prescribed for episodes of infection

• *(including respiratory infections).*

12. Maternal health related quality of life (EQ-5D-5L)

• *A measure of a mother's quality of life using a specially designed questionnaire to understand how their baby's health may impact on their health and quality of life.*

13. Child health related quality of life (PedsQL; Parent reported, collected at age 24 months)

• *A measure of the baby's quality of life, as reported by a parent. This is measured using a specially designed questionnaire and is done at 2 years of age because this is the youngest age that it can reliably be done.*

44. Please rank three outcomes that you feel are most important to be measured in the TOAST trial in order of importance. The first most important outcome for the TOAST study is: *

Mark only one oval.

- Severity of anastomotic stricture
- Incidence of anastomotic stricture
- All-cause mortality
- Number of complications directly related to oesophageal atresia post-surgery
- Adverse events
- Presence of symptoms of gastro-oesophageal reflux
- Treatment of reflux symptoms
- Weight
- Respiratory symptoms
- Oral feed intake
- Number of courses of antibiotics prescribed for episodes of infection
- Maternal health related quality of life
- Child health related quality of life

45. The second most important outcome for the TOAST study is: *

Mark only one oval.

- Severity of anastomotic stricture
- Incidence of anastomotic stricture
- All-cause mortality
- Number of complications directly related to oesophageal atresia post-surgery
- Adverse events
- Presence of symptoms of gastro-oesophageal reflux
- Treatment of reflux symptoms
- Weight
- Respiratory symptoms
- Oral feed intake
- Number of courses of antibiotics prescribed for episodes of infection
- Maternal health related quality of life
- Child health related quality of life

46. The third most important outcome for the TOAST study is: *

Mark only one oval.

- Severity of anastomotic stricture
- Incidence of anastomotic stricture
- All-cause mortality
- Number of complications directly related to oesophageal atresia post-surgery
- Adverse events
- Presence of symptoms of gastro-oesophageal reflux
- Treatment of reflux symptoms
- Weight
- Respiratory symptoms
- Oral feed intake
- Number of courses of antibiotics prescribed for episodes of infection
- Maternal health related quality of life
- Child health related quality of life

47. Are there any other outcomes that you think are important primary or secondary outcomes to measure for this trial that are not included in the list above?

Mark only one oval.

- Yes *Skip to question 48*
- No *Skip to question 51*

48. Please provide your suggestion for an additional outcome here and state whether it is a primary or secondary outcome *

____

____

____

____

____

49. Please provide your suggestion for an additional outcome here and state whether it is a primary or secondary outcome

____

____

____

____

____

50. Please provide your suggestion for an additional outcome here and state whether it is a primary or secondary outcome

____

____

____

____

____

For peer review only - http://bmjopen.bmj.com/site/about/guidelines.xhtml

Follow up is currently planned to occur at 3, 6, 9 and 12 months to coincide with routine clinic visits and some will be measured though parental reports via an App. You will be free to see these infants more often should you wish. This will include asking specifically about the treatment of anastomotic strictures and more generally about other symptoms and adverse effects.

51. Is there anything you feel should be specifically addressed in follow up? *

Mark only one oval.

- Yes *Skip to question 52*
- No *Skip to question 53*

52. Please elaborate *

Part 5: Recruitment and consent

We propose that babies will be randomised into the medication or placebo group within 72 hours of their oesophageal surgery, when their condition has stabilised.

53. How acceptable do you think it is to approach parents to discuss the trial at this point in time? *

Mark only one oval.

- Very acceptable
- Acceptable
- Neutral
- Not acceptable
- Not at all acceptable

For peer review only - http://bmjopen.bmj.com/site/about/guidelines.xhtml

54. Please elaborate

55. Would you have any concerns about approaching parents to seek consent for their baby's inclusion in TOAST? *

Mark only one oval.

- Yes
- No

56. Please elaborate

57. How concerned are you about the ability to retain participants in the TOAST trial? *

Mark only one oval.

- Not at all concerned
- A little concerned
- Very concerned

58. If you have any concerns about the retention of participants in the TOAST trial and/or any suggestions to help address concerns about the retention of participants, please elaborate below

59. Do you envisage any practical or logistical challenges in delivering TOAST at your unit? *

Mark only one oval.

- Yes
- No

60. Please elaborate

Part 6: Overall acceptability and training

61. Overall, how acceptable do you find the proposed TOAST trial? *

Mark only one oval.

- Very acceptable
- Acceptable
- Neutral
- Not acceptable
- Not at all acceptable

62. Please elaborate

63. Is there anything specific that you would suggest we include in the TOAST site training package? (e.g., training content, who should be trained, whether any online resources would be needed in addition to site initiation visit) *

64. Do you have any other comments or concerns about the TOAST trial? *

Mark only one oval.

- Yes *Skip to question 65*
- No *Skip to section 27 ()*

65. Please note any other comments or concerns about the TOAST trial here

We plan to conduct further research into clinicians' attitudes to the trial via more in depth focus group or telephone interview discussions. These will be held online via Zoom or a similar platform. Please add your contact details if you would like to register interest in taking part in a focus group or interview. Your details will not be used for any other purposes and will be stored separately from questionnaire data

First name and email address

66. First name

67. Email address

Thank you for your participation in this survey.

This content is neither created nor endorsed by Google.

Google Forms

TOAST Parent interview topic guide

Please note: ***Italic text indicates instruction for researcher and will not be read to participant***

Intro: My name is xxx and I am a researcher from the University of xxx. Many thanks for agreeing to help us with the design of the TOAST study.<br>Before we begin the interview I need to obtain your consent for the study is that ok? (*Refer to instructions at top of Participant Consent Form (re: reading 8 statements, sending a copy and consent for audio recording of this discussion).*

- **Obtain consent here**

You can stop the interview at any time. Before we start do you have any questions?<br>Have you had chance to look at the draft participant information sheet I sent to you for the TOAST trial? (If no- read through sheet with parent)

**Section one- Parent details and experience**	
1.1	For administrative purposes: your age, occupation, first part of post code, ethnic background, number and ages of children, where saw the study advertised.
1.2	My notes from when you registered interest in taking part in this study state that your child was born with oesophageal atresia (*insert month and year*). Is that correct?<br>I have a few questions about your child's experience is that ok?<br>*Explore:*<br>When was your child's oesophageal atresia first identified or diagnosed?<br>When did your child have surgery to reconstruct their oesophagus? (Days or weeks after birth)<br>What treatments or medication did your child receive after this surgery? (Prompt; explore if they received gastric acid suppression medication or if it had been discussed by the doctor/surgeon?)<br>Did your child experience a narrowing of their oesophagus following this surgery? *If yes,* could you tell me a little bit about this? How did the stricture impact on your child? *If relevant,* how many dilations has your child had?<br>Has your child had (or expected to have) treatments for other conditions, which may have impacted upon the oesophageal surgery?<br>Do you access support groups, for example, online groups such as TOFS? (If yes: explore if the group have provided any useful advice about treatments or medication?)
	**We are in the process of developing a type of study called a clinical trial. Have you ever heard of a clinical trial before?**<br>*Explain:* This is a type of medical research which provides information on the safety and effects of a drug or medical device (for example, a needle or line used to administer drugs). They are used to find out the best way to treat patients in the future. Trials are carried out to test many different medicines or treatments for children. Some test medicines or treatments which we think will be low risk, because doctors have already been using them for some time. Some trials test new medicines or ones where we don't know exactly what effects they will have in a particular situation.

For peer review only - http://bmjopen.bmj.com/site/about/guidelines.xhtml

	**Have you ever been asked if your child could take part in a clinical trial?** *If yes,*<br>Could you tell me a little more about that?<br>What was the trial looking at?<br>At what point where you approached and asked if you would give consent for your child to take part in the trial?<br>Could you tell me a bit more about what happened? How did you feel at the time?
**Section two- Baseline Knowledge of TOAST**	
2.1	Have you had chance to look at the draft participant information sheet I sent to you for the proposed TOAST study, which is a clinical trial (If no- read through sheet with parent)?
2.2	Based on the participant information sheet please describe your understanding of what the TOAST study is aiming to do?
**Section three- TOAST**	
3.1	**Talk though the draft trial PIL** *Prompt:* Is each of these sections clear? Is there anything we need to add or change?
3.2	Looking at the information sheet, are there any parts of the study design that you think parents may find difficult to understand?
3.3	What would you think if someone approached you about a study like this, explained to you whilst your child was in NICU?<br>What would be your initial thoughts about this proposed study?<br>When do you think would be best time to be approached about the study? (*Prompt, after initial response explore views on time frame proposed*).
3.4	Who do you think would be the best person to approach parents to discuss the TOAST study *(explore views on it being a research nurse, doctor, surgeon)?*
3.5	Would you have any concerns about the proposed TOAST study?
3.6	Would you have any questions about the TOAST study?
3.7	How much time would you need to consider the information before making a decision about the TOAST study?
3.8	For babies who are sent home from hospital, parents will be asked to give their child the gastric acid suppression medication every day as part of the TOAST trial. We are designing an app which will send reminders to parents to help them remember to give the medication<br>*(Prompt explore thoughts on this, timing of notification, how many per day and for how long, storage of medication in fridge and anything parents think might help stop people dropping out of the trial over time, preparation of meds, expiry dates (although we are limited on this), whether gave medication or not, dosing with weight changes, follow up at clinic at 3,6,9,12 months and additional questionnaires)*

For peer review only - http://bmjopen.bmj.com/site/about/guidelines.xhtml

3.9	The information sheet describes how each parent would be approached and permission would be sought for their baby's involvement in the TOAST trial. If informed consent is provided, their child will be randomly allocated to either group A (no gastric acid suppression medication) or group B (Gastric acid suppression medication).<br><br>An alternative approach is called 'opt out' consent. This is when all eligible babies are automatically randomly allocated to either group A of group B following their surgery. There will be posters up on the walls of NICU explaining the trial and then parents will be approached with an information sheet and asked if they would like to opt their baby out of the trial. If they 'opt out' then no further information is collected from that point forward and their baby will receive whatever the usual treatment is in that NICU.<br><br>*(Check understanding and clarify any queries)*<br><br>What are your thoughts about an opt out approach for the TOAST trial?<br><br>Do you think the TOAST trial should use an informed consent or opt out approach? *(Explore reasons).*
	Does your child currently take any antacid medications?<br><br>In TOAST, if participating children are given additional gastric acid suppression medication this will make the results hard to interpret. So they would be given anti-reflux and other medications first (see flow chart) and only allowed to have additional medication (e.g. omeprazole) if these fail. How would you feel about that? Would that influence your views on taking part in the study? *(Explore any information that parents feel we should prioritise in the trial PIS)*
3.10	How acceptable do you feel it is to give anti reflux medication to half the babies who take part in this study?
3.11	How acceptable do you think it is to not give anti reflux medication to half the babies in this study?
3.12	What are your thoughts about the suggested pathway to managing symptoms of reflux flowchart? *Explore if:* it addressed any concerns, raised any concerns they may not have had, is it easy to understand? Anything unclear or missing?
3.13	Is there anything you would find useful when deciding whether or not to allow your child's participation? (*Prompt:* what would be the top two/three issues that would make you positive about giving permission for your child to take part? And what would be the main two/three points that might make you want to say no?
3.14	Would you have given your permission for your child's to take part in the TOAST Trial? *(Prompt: Could you tell me a bit more about your reasons for this?)*
**Section 4- Outcome measures**	
As we have discussed, in the TOAST study we want to find out if routinely giving babies gastric acid suppression medication after repair of oesophageal atresia will reduce the incidence or severity of oesophageal stricture.<br><br>To do this we will collect information on Outcomes *(the list that I sent to you prior to interview).*<br><br>By collecting information on these main things, we hope to find out which should be used in the future. These are called outcome measures.	

For peer review only - http://bmjopen.bmj.com/site/about/guidelines.xhtml

However, these outcomes have come from research papers and don't really give us much information on how children or families feel, or what is important to them. It is important that we include outcome measures that matter to children and their families.		
4.1	Thinking about your experience of your child's surgery and their health since that point in time, what would you hope the gastric acid suppression medication would do to help your child? (*Prompt: what effect would the medication have to be useful?*)	
4.2	What would you be looking for as an indicator that the medication was helping your child?	
4.3	What do you think about the outcome measures (*re-cap measures in the list provided*)<br>Is there another outcome measure that you think is important to families which we should be collecting information about in the TOAST Study?	
4.4	*Recap on outcomes measured and ask them to put in order of importance* (e.g. So far you have mentioned x outcomes, X, Y & Z. which would you say is the most important for this study? Second most important for this study?)	
**Section 5- Concluding comments**		
Finally, is there anything else you would like to say about this proposed trial?		
If we do find that it is feasible to do a trial, would you potentially be interested in being on a parent advisory group for the study? We would of course contact you and provide further information about what would be involved and would check this with you again as it may be some time in the future.		

For peer review only - http://bmjopen.bmj.com/site/about/guidelines.xhtml

COREQ (COnsolidated criteria for REporting Qualitative research) Checklist

A checklist of items that should be included in reports of qualitative research. You must report the page number in your manuscript where you consider each of the items listed in this checklist. If you have not included this information, either revise your manuscript accordingly before submitting or note N/A.

Topic	Item No.	Guide Questions/Description	Reported on Page No.
**Domain 1: Research team and reflexivity**			
Personal characteristics			
Interviewer/facilitator	1	Which author/s conducted the interview or focus group?	
Credentials	2	What were the researcher's credentials? E.g. PhD, MD	
Occupation	3	What was their occupation at the time of the study?	
Gender	4	Was the researcher male or female?	
Experience and training	5	What experience or training did the researcher have?	
Relationship with participants			
Relationship established	6	Was a relationship established prior to study commencement?	
Participant knowledge of the interviewer	7	What did the participants know about the researcher? e.g. personal goals, reasons for doing the research	
Interviewer characteristics	8	What characteristics were reported about the inter viewer/facilitator? e.g. Bias, assumptions, reasons and interests in the research topic	
**Domain 2: Study design**			
Theoretical framework			
Methodological orientation and Theory	9	What methodological orientation was stated to underpin the study? e.g. grounded theory, discourse analysis, ethnography, phenomenology, content analysis	
Participant selection			
Sampling	10	How were participants selected? e.g. purposive, convenience, consecutive, snowball	
Method of approach	11	How were participants approached? e.g. face-to-face, telephone, mail, email	
Sample size	12	How many participants were in the study?	
Non-participation	13	How many people refused to participate or dropped out? Reasons?	
Setting			
Setting of data collection	14	Where was the data collected? e.g. home, clinic, workplace	
Presence of non-participants	15	Was anyone else present besides the participants and researchers?	
Description of sample	16	What are the important characteristics of the sample? e.g. demographic data, date	
Data collection			
Interview guide	17	Were questions, prompts, guides provided by the authors? Was it pilot tested?	
Repeat interviews	18	Were repeat inter views carried out? If yes, how many?	
Audio/visual recording	19	Did the research use audio or visual recording to collect the data?	
Field notes	20	Were field notes made during and/or after the inter view or focus group?	
Duration	21	What was the duration of the inter views or focus group?	
Data saturation	22	Was data saturation discussed?	
Transcripts returned	23	Were transcripts returned to participants for comment and/or	

For peer review only

Topic	Item No.	Guide Questions/Description	Reported on Page No.
		correction?	
**Domain 3: analysis and findings**			
Data analysis			
Number of data coders	24	How many data coders coded the data?	
Description of the coding tree	25	Did authors provide a description of the coding tree?	
Derivation of themes	26	Were themes identified in advance or derived from the data?	
Software	27	What software, if applicable, was used to manage the data?	
Participant checking	28	Did participants provide feedback on the findings?	
Reporting			
Quotations presented	29	Were participant quotations presented to illustrate the themes/findings? Was each quotation identified? e.g. participant number	
Data and findings consistent	30	Was there consistency between the data presented and the findings?	
Clarity of major themes	31	Were major themes clearly presented in the findings?	
Clarity of minor themes	32	Is there a description of diverse cases or discussion of minor themes?	

Developed from: Tong A, Sainsbury P, Craig J. Consolidated criteria for reporting qualitative research (COREQ): a 32-item checklist for interviews and focus groups. *International Journal for Quality in Health Care*. 2007. Volume 19, Number 6: pp. 349 – 357

Once you have completed this checklist, please save a copy and upload it as part of your submission. DO NOT include this checklist as part of the main manuscript document. It must be uploaded as a separate file.

For peer review only

TOAST

Treating Oesophageal Atresia to Prevent Stricture

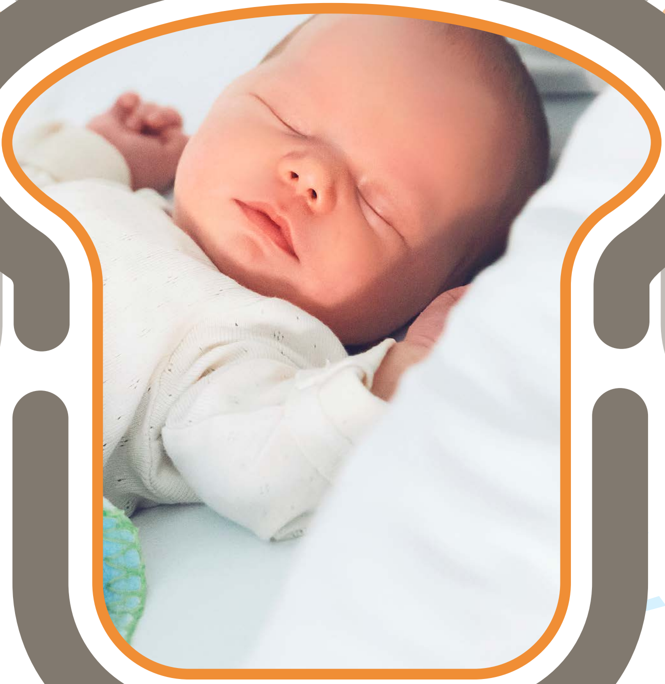

Parent Information Leaflet

For peer review only - http://bmjopen.bmj.com/site/about/guidelines.xhtml

For peer review only

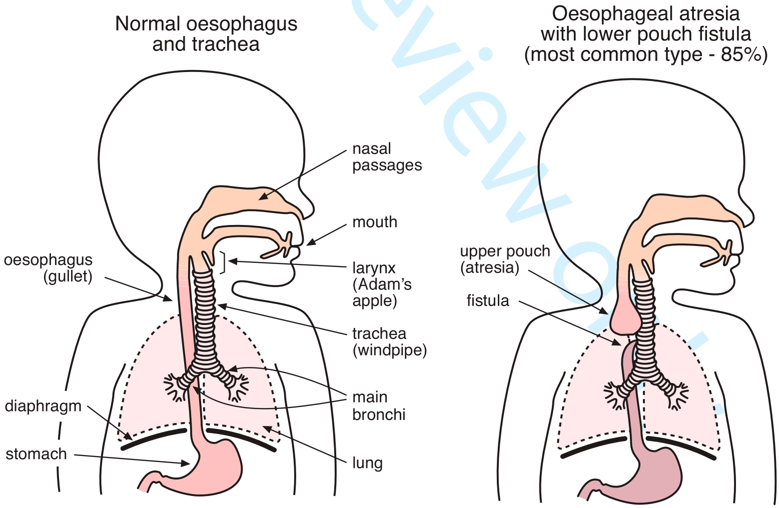

From Jaffray, B. (2016) 'Introduction to OA/TOF', in Martin, V. and Crabbe, D. (Eds.) The TOF Book. 2nd Edition. Nottingham, UK: TOFS., p. 17. ISBN 978-0-9536265-1-9. Copyright 2016 by TOFS. Reprinted with permission.

We understand that this is a difficult time for you and your family. We would like to invite you to take part in a study this hospital is taking part in for babies who have been born with oesophageal atresia. Before you decide it is important that you understand why the research is being done and what it will mean for you and your baby. Please ask us if there is anything else that is not clear or you would like to know.

Why do we do research in healthcare?

Research is really important so that we can improve how we treat patients. If no research took place, then it would be difficult to improve outcomes for patients. All research in the NHS is voluntary.

Why are we being asked to take part?

Your baby has been diagnosed with oesophageal atresia, where the oesophagus (swallowing tube) has not formed properly and so your baby's mouth is not connected to their stomach. The treatment for this condition involves surgery to reconstruct the oesophagus. The team looking after your baby will have talked to you about what this surgery involves.

We are asking you to help us with a research project to learn more about how treatment following this surgery can be made better. The study is called TOAST which stands for Treating Oesophageal Atresia to prevent STricture.

Why are we doing this research?

One of the complications that can follow surgery to reconstruct the oesophagus is a narrowing called a stricture at the point where the repair is made. A stricture in the oesophagus would make it difficult for your baby to feed. Between 40% and 50% of babies get a stricture in the months after repair of oesophageal atresia. A stricture is usually treated by stretching the narrow section to make it wider in a procedure called a "dilatation". This requires an admission to hospital and is done under general anaesthetic. Some babies require several dilations during the first year of life.

Gastro-oesophageal reflux (where the stomach contents go back up into the

For peer review only - http://bmjopen.bmj.com/site/about/guidelines.xhtml

oesophagus) is also very common after repair of oesophageal atresia and some surgeons think that reflux of acid into the oesophagus may make a stricture more likely or more severe. To try and prevent strictures from forming some surgeons use gastric acid suppression medication (e.g. omeprazole, 1ml/kg/day) in all babies, even if they have no symptoms of reflux, after repair of oesophageal atresia. The aim of using this medicine is to reduce stomach acid secretion so that even if there is reflux it is not as acidic.

Despite the common use of gastric acid suppression medication, we do not know for certain if there is any benefit to its use in babies following surgery. Indeed, some studies have indicated that babies routinely given gastric acid suppression medication may be more likely to get a stricture, but the evidence is not conclusive. There are other reasons why giving gastric acid suppression medication may not be a good idea, including that they may slightly increase the risk of infections and concerns about giving medicines to babies without proven benefit.

For these reasons, we are carrying out the TOAST study to find out if giving gastric acid suppression medication does help babies with oesophageal atresia. If it does, then all babies with oesophageal atresia can be given this treatment. If it does not, then we can stop asking parents to give medicine to their baby that has no benefit.

What will happen if we take part?

The study we are asking you to give permission for your child to take part in is a randomised controlled trial (RCT). In the trial half of the babies will be given an gastric acid suppression medication from soon after their surgery to repair the oesophageal atresia and the other half will be given a cellulose/water solution as a ‘dummy’ medication called a placebo. We will use a computer to decide at random whether your baby receives gastric acid suppression medication or the placebo. The chance of your baby receiving either gastric acid suppression medication or placebo is equal. No one will know whether your baby is receiving the gastric acid suppression medication or the placebo. This includes you, the medical team and the research team. The reason a placebo is important is to make sure we are carrying out a fair test.

Your baby will be given the treatment each day by the medical team in hospital. When your baby is discharged home, we will ask you to give the treatment to your baby once daily for one year after their surgical repair. The doctors and nurses will show you how to do this. You will be asked to complete questionnaires about your baby’s progress, how much treatment you have given, hospital visits and how you are feeling. These will be completed through an app, online or

For peer review only - http://bmjopen.bmj.com/site/about/guidelines.xhtml

on paper if you prefer. The doctors and nurses will provide you with further treatment supplies at your routine hospital visits and will be available to answer any questions you might have.

We will then follow your baby up for the first two years of their life to see if they develop a stricture and to monitor their progress. You will be asked to complete questionnaires at 3, 6, 9, 12, and 18 months, and 2 years about your baby's progress, how much treatment you have given, hospital visits and how you are feeling. These will be completed through an app, online or on paper if you prefer. Your baby will be followed up for the duration of their childhood whether they take part in this study or not and we will use these routine follow up visits to collect information relevant to the study so you will have the minimum inconvenience. If your baby does develop a stricture then they will need treatment and your doctors will explain this to you. At the end of the study in around 2028 we hope to have included about 211 babies born with oesophageal atresia. This is a number which we believe is big enough to give convincing results, and is far bigger than reported in any similar studies done worldwide to date.

We have tried to make taking part in the study as easy as possible for you and your family. The follow up has been designed around routine follow up visits that would happen for all babies born with oesophageal atresia so it should not mean extra visits to the hospital.

What are the possible benefits or risks of taking part?

The treatments used in the study are used routinely for babies born with oesophageal atresia in UK hospitals and are known to be safe.

This study will not bring any immediate benefit to your child. We hope that we will get information about how best to treat children born with oesophageal atresia in the future. By participating, you will be helping us to learn whether treatment with gastric acid suppression medication is better than none so that we will know whether to offer this routinely as a treatment for other children in the future. After we have finished the study, we can let you know the results if you would like.

Do we have to take part?

No, taking part is completely voluntary. If you decide not to take part, you do not have to give a reason and your child's care will not be affected in any way. If you agree to take part, we will ask you to sign a consent form which indicates your agreement to take part in the study and to let the researchers look at your child's health records. We will put a copy of this research consent form in your

For peer review only - http://bmjopen.bmj.com/site/about/guidelines.xhtml

child's health records. We will give you a copy for your files.

How long do I have to decide?

Because we would like to start treating your child soon after their operation to repair the oesophagus we will need to know if you are willing to take part within the next few days. To help you decide please talk about the study to your doctor. Please ask them any questions you may have.

What if I change my mind?

You can change your mind about taking part at any time and remove your baby from the study without giving a reason. We would then only use the information we have collected up to the point when you withdraw. This would not affect the care your baby receives in any way.

Will my details be kept confidential?

Your GP will be told that your baby is taking part in this study. We will collect personal information about you and your baby; the details will be kept securely and will only be seen by the research team, the study organisers in Oxford and people from the sponsor or regulatory authorities who check on studies such as this - this is a routine process of research in the NHS.

All information that we collect about you and your baby during the study will be kept strictly confidential and stored securely. We might ask for permission to contact you again in the future to find out how your baby is getting on as they grow up. If you decide to take part, we will collect some personal information about you and your baby, including name, NHS number, date of birth, address and contact details (e.g., email address and telephone number). This information will be sent to the Study Coordinating Centre at the University of Oxford, National Perinatal Epidemiology Unit Clinical Trials Unit (NPEU CTU). Authorised members of the research team will hold this data and store it securely for the purposes of running the trial. Authorised staff from the University of Oxford (as Coordinating Centre and Sponsor), funder, regulatory bodies, and your hospital may be given access to data for monitoring and/or audit of the study to ensure the research is complying with applicable regulations. Personal identifiable information including your telephone number and email address will be shared with Blue Frontier App provider for the purposes of recording the amount of medication given. The Study Coordinating Centre in Oxford will keep identifiable information about you and your baby from this study for 25 years after the study has finished. For more information on how we process and protect you and your baby's data, please see our website: ***https://www.npeu.ox.ac.uk/ctu/privacy-notice***

For peer review only - http://bmjopen.bmj.com/site/about/guidelines.xhtml

Further information can also be found at the NHS Health Research Authority's website:

https://www.hra.nhs.uk/planning-and-improving-research/policies-standards-legislation/data-protection-and-information-governance/gdpr-guidance/templates/template-wording-for-generic-information-document/

Please see our website for further information about how we protect your data: ***https://www.npeu.ox.ac.uk/ctu/privacy-notice***

Who is organising and funding the study?

The study is funded by the National Institute of Health Research (NIHR) which is the research arm of the NHS. The study is sponsored by the University of Oxford and is being run by the National Perinatal Epidemiology Unit Clinical Trials Unit (NPEU CTU) at the University of Oxford. The researchers involved in the study are all experienced researchers, interested in improving the care given to babies born with oesophageal atresia. A number of parents have also helped design the study, as have TOFS, the UK support group for families affected by oesophageal atresia.

All research in the NHS is looked at by an independent group of people, called a Research Ethics Committee, to protect your interests and make sure it is done to the highest standards. This trial has been reviewed and given favourable opinion by a Research Ethics Committee.

What will happen to the results of the research study?

At the end of the study, the results will be analysed and published in a medical journal. We will write our reports in a way that no one can work out who took part in the study. We will also share the results at medical and surgical conferences and with families of children with oesophageal atresia via the TOFS support group, who have been involved in setting up the trial. We will send you a copy of the results at the end of the study and we will also share them on our website. Unidentifiable data from this study may be shared with other groups who are carrying out similar work in the future.

What happens if I have a complaint because of my baby taking part in this study?

In the first instance you can talk to the clinical team looking after your baby who will help you with your concern. You can also contact the local research team, either the research nurses or the principal investigator, their contact details are on the back page of this leaflet. If you wish to complain about any aspect of

For peer review only - http://bmjopen.bmj.com/site/about/guidelines.xhtml

the way that you or your baby has been treated you may use the normal National Health Service complaints procedures, the Patient Advice and Liaison Service at your hospital will advise you about this (their contact details are also on the back page of this leaflet).

In the unlikely event your baby has been harmed by taking part in this study, you may have grounds for legal action and could seek compensation through the Research Sponsor, the University of Oxford, who has appropriate insurance-related arrangements in place. If your baby is harmed and it is due to any routine clinical treatment or negligence then the usual NHS indemnity arrangements will apply.

Contact Information

Chief Investigators:

Mr Nigel Hall, University of Southampton

Mr Iain Yardley, Evelina London Children's Hospital.

Local Contact Details:

Principal Investigator:

{_LEAD_}

Thank you for taking the time to read this information sheet

TOAST Study Team

NPEU Clinical Trials Unit,
National Perinatal Epidemiology Unit, University of Oxford,
Old Road Campus, OXFORD, OX3 7LF

01865 617919 toast@npeu.ox.ac.uk www.npeu.ox.ac.uk/toast

For more information about the TOFS support group please visit their website:
www.tofs.org.uk/about-us.aspx

FUNDED BY NIHR | National Institute for Health Research

TOAST is funded by the National Institute for Health Research (NIHR) Health Technology Assessment programme (project reference 131136).
The views expressed are those of the author(s) and not necessarily those of the NIHR or the Department of Health and Social Care.

For peer review only - http://bmjopen.bmj.com/site/about/guidelines.xhtml

TOAST

Evidence - and guideline - based rational decision tree for treating possible reflux symptoms in postoperative OA/TOF

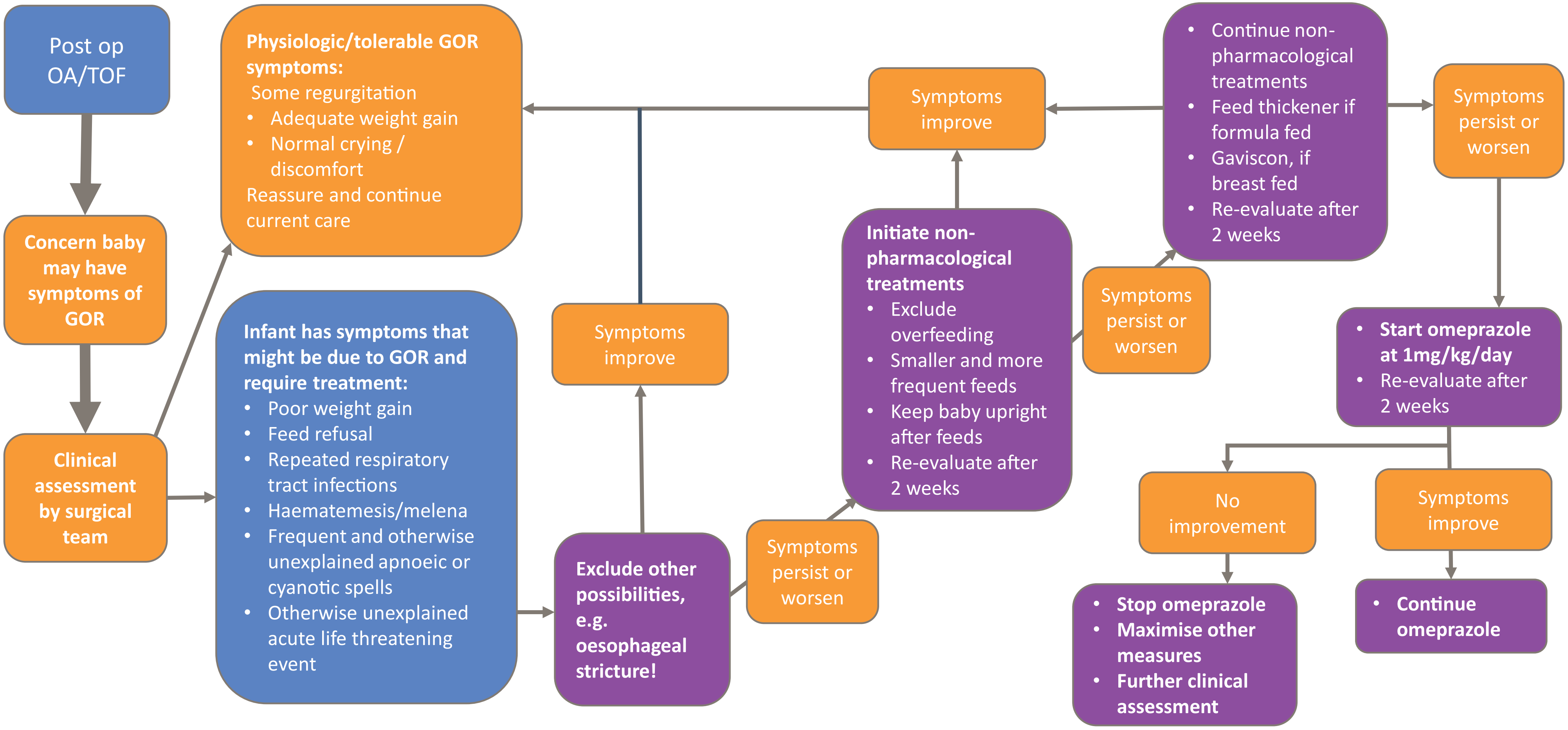

N.B. If you feel that urgent treatment is needed, clinical judgement takes precedence over the above.

TOAST

Treating Oesophageal Atresia to Prevent Stricture

Toast Trial: Outcomes

Severity of anastomotic stricture

- The number of dilations performed within one year of trial entry.

Incidence of anastomotic stricture

- Whether your baby has any dilatation or none in the first year / 2 years of their life.

Other complications directly related to oesophageal atresia

- Anything that is directly related to oesophageal atresia or its repair, for instance leakage from the join in the oesophagus.

Adverse events

- A general term used to describe things that don't go as planned but aren't included in other outcomes.

Presence of symptoms of gastro-oesophageal reflux

- Symptoms that may be due to reflux and reported by parents.

Need for treatment of reflux

- Whether your baby is given any sort of medication to treat reflux (e.g., feed thickener, Gaviscon).

Growth

- Your baby's growth during the first year of their life (recorded by weight, length and head circumference at certain time points).

Respiratory symptoms

- Presence of respiratory symptoms such as infections, chronic cough (but not the usual TOF cough) or any other symptoms that a doctor felt required investigation or treatment.

Oral Intake

- How your baby is progressing with advancing from milk feeds onto solids at specific ages.

Episodes of infection

- Any episode of proven infection (including respiratory infections).

Survival

- Whether your child survived to a certain time point (usually time point at months/years) or to a specific event (e.g., hospital discharge).

Maternal Health Related Quality of Life (EQ-5D-5L)

- A measure of a mother's quality of life using a specially designed questionnaire to understand how their baby's health may impact on their health and quality of life.

Child Health Related Quality of Life (PedsQL; Parent reported, collected at age 24 months)

- A measure of your baby's quality of life, as reported by a parent. This is measured using a specially designed questionnaire and is done at 2 years of age because this is the youngest age that it can reliably be done.

TOAST Study Team

NPEU Clinical Trials Unit, University of Oxford,
Old Road Campus, Headington, Oxford, OX3 7LF.
01865 617919 toast@npeu.ox.ac.uk
www.npeu.ox.ac.uk/toast

TOAST is funded by the National Institute for Health Research (NIHR) Health Technology Assessment programme (project reference 131136). The views expressed are those of the author(s) and not necessarily those of the NIHR or the Department of Health and Social Care.

For peer review only - http://bmjopen.bmj.com/site/about/guidelines.xhtml

SUPPLEMENTARY FILE 8

a) Parent suggested edits to the symptomatic reflux treatment pathway

In the Blue box ***Infant has symptoms that might be due to GOR and require treatment*** six parents suggested the following be added:<br>• *'Clear visible pain'*/ *'clear discomfort'* (P4, mother)/ being really *'uncomfortable'* (P3; P5, mothers) or having a *'sore tummy'* (P9, mother) - *'arching their back'* (P3; P4; P6, mothers), *'pushing their tummy out'* (P5, mother) or *'squirming'* (P3; P5, mothers).<br>• *'Coughing'* (P12; P3, mothers) (not the usual TOF cough) and *'retching'* (P3, mother).<br>• *'Abnormal'* (P4, mother) or *'extreme'* (P3, mother) *'crying'* (P4; P5, mothers) or *'screaming'* (P4, mother).<br>• *'Not settling after feeds'* (P5, mother).<br>• *'Bringing up milk, even after holding them up for half an hour'* (P6, mother).<br>• *'Dry nappies'* (P9, mother).
**In the purple *Exclude other possibilities, e.g., oesophageal stricture box***, add *'if they [GOR symptoms] are in combination with other usual stricture signs*' (P5, mother) (albeit, some parents did not receive much information about (P3, mother) or know what a stricture was at the beginning of their journey with a child with OA (P3; P9; P10, mothers). Some mothers did not know the signs of stricture (see below) to look out for (P5; P9, mothers). Furthermore, some parents were not told the word *stricture* or *dilatation* – terms such as *'narrowing'* or *'tightening'* (P12, mother) and *'stretch'* were used instead (P9, mother). Parents said that the other difficulty is that most signs of stricture are similar to those of reflux, such as:<br>• Coughing (P3; P5, mothers) or choking (P5; P15, mothers; P8, father), refusing (P5, mother), or not being able to swallow milk, food (P8, father; P12, mother) or saliva (P11, mother).<br>• Milk coming out of their nose (P5, mother).<br>• Not finishing milk feeds (P3, mother).<br>• Vomiting after feeds (P8; P17, fathers; P9; P13; P15, mothers).<br>• 'Blue events' when their child stopped/were struggling to breathe when trying to feed or lying down (P4; P5; P9; P12, mothers; P16; P18, fathers), or upset (P18, father):
**Have a further box under *Start omeprazole - symptoms improve*** for when children *'are better than [they were but are] still having a problem'* (P10, mother) and need the dosage increasing (P5, mother). Four mothers (P4; P5; P10; P12) felt that *'Omeprazole at 1mg per kilogram a day… [is] still quite a low dose of Omeprazole'* (P4, mother), with one child being *'started on 2mg per kilogram when he was in hospital'* (P12, mother) and another being on *'30mg per day … Why would they not pursue 'increase to maximum dose' and then 'symptoms improve, continue.' 'No improvement, stop Omeprazole.' Why stop the treatment before you've reached the maximum dose?'* (P4, mother).
Under ***Initiate Non-Pharmacological Treatments***, as well as holding the baby upright after feeds (P3; P5, mothers; P16, father), add:<br>• *'Keep stopping every five sucks'* if bottle fed to pace the feed (P3, mother).<br>• Remove *'animal milk protein from the mother's diet if the child is being breastfed, or from the formula, if the child is formula-fed'* (P4, mother).<br>• *'Incline the bed'* (P12, mother; P16, father)/*'Tip your cot at an angle'* (P4, mother) to ensure that the crib/cot/bed is higher at the head end (P10, mother). *'Things like propping them up and wedges … are standard things for a lot of TOFS children that might not get mentioned in a medical sense'* (P11, mother).

For peer review only - http://bmjopen.bmj.com/site/about/guidelines.xhtml

• *'Any of the above [reflux] symptoms when parent has already initiated non-pharmacological treatment'* (P4, mother).
Explain acronyms such as *'GOR'* (P9; P10; P14, mothers; P18, father) or to have a *'key to terms'* (P12, mother) at the bottom of the diagram.<br>Explain medical terminology such as haematemesis/melena and unexplained apnoeic or cyanotic spells – perhaps word these as *'blue events'* (P12, mother), *'life-threatening events'* (P5, mother) or *'desaturations'* (P3, mother) because *'different hospitals … use slightly different terms'* (P12, mother) and some parents did not know what these were when worded this way (P3; P10, mothers), even though they learn *'pretty quickly'* (P10) what these are.
Consider accessibility:<br>*'Some people are colour blind and might find it hard to read on all the different colours', so it might be best to make the colours a darker shade to increase the contrast between the colours and the text'* (P13, mother).<br>*Left align all text (rather than some being 'centred')* (P13, mother).<br>Send the arrow from the first *Symptoms improve* box directly to the Physiologic/tolerable GOR symptoms box.

b) Clinician suggestions for edits to the symptomatic reflux treatment pathway

'I think the signs in the blue box need splitting into minor and minor. For example, poor weight gain or food refusal' (C20, surgeon, survey).
'Good flow chart - maybe mention in other conditions: consider tracheomalacia, as this would mimic reflux closely, but in some cases may require urgent surgical intervention' (C22, surgeon, survey).
'I think it should be more explicitly pro breast feeding. Breastfeeding should come above artificial feeding in each list. Some may advocate investigation (e.g., pH study, contrast swallow etc) at some point. Where will that fall? Feeding in a more upright position as well as nursing after feeding in an upright position may help. In the normal physiology box, you could add something about regular bowels and wet nappies (to be reassuring)' (C41, neonatologist, survey).
'Not clear in pathway when formal evaluation of GOR (pH/impedance)' (C46, surgeon, survey).
'In the pathway you have put exclude oesophageal stricture before excluding overfeeding and keeping the baby upright after feeds. I think this is the wrong way round because excluding stricture would involve endoscopy or contrast - not sure you would go to that step before you prop the baby up a little?' (C35, surgeon, survey). This quote supports that of one mother who said *'Obviously with the reflux and the GOR and things like that, they will go off and do the contrast studies… diagnosing whether reflux is an issue or not'* (P11, mother).
'What if a baby has gross clinical reflux / BRUEs consistent with severe reflux? Normally I would maximise PPI before taking the next steps? What if I perform a fundoplication in the first year of life?' (C4, surgeon, survey).
'What about anti-reflux surgeries? At what point would these be considered?' (C27, surgeon, survey).

For peer review only - http://bmjopen.bmj.com/site/about/guidelines.xhtml

SUPPLEMENTARY FILE 8 Edits and additions for predefined outcomes

Predefined outcome	Suggested edits or additions
'Presence of symptoms of gastro-oesophageal reflux'	Collect data on tube feeding:<br>*[Child's name]'s reflux was a lot worst when he had the nasogastric tube and when the tube come out his instances of vomiting dramatically decreased. So, if children go home with a nasogastric tube, it might be interesting as part of the study to see the ones that had the antacid medicine and the ones that didn't to see the sort of effects of both* (P13, mother, interview).<br>Collect data on the *'number of [hospital] admissions related to GER'* (C25, surgeon, survey).
'Oral intake' and 'Growth'	Measure the age of solid food intake (P4; P15, mothers, interviews; P17, father, interview).<br>Collect data on *'the use or non-use of a gastrostomy'* (P17, father, interview), '*nasogastric tube'* (P13, mother, interview) or *'jejunal feeding without fundoplication'* (C47, surgeon, survey) because *'neonatologists and paediatricians will be… interested in feeding, nasogastric tube feeding and growth parameters'* (C41, neonatologist, focus group 2) / *'weight gain velocity'* (C21, surgeon, survey). '*That is the first question that comes into your mind when you are thinking about the quality of life for those children during the first and second year of their life'* (C41, neonatologist, focus group 2).
'Respiratory symptoms'	*'Add bronchiolitis'* (P15, mother, interview).<br>Measure *'any near-death attacks'* (C25, surgeon, survey) / *'ALTE'* [apparent life-threatening event/BRUE – brief, resolved, unexplained event] (C47, surgeon, survey).
'Episodes of infection'	*'Add bronchiolitis'* (P15, mother, interview) and aspiration (P5; P9, mothers, interviews; P18, father, interview):<br>*'You already have respiratory symptoms as an outcome but I wonder if evidence of aspiration would be better because it is really aspiration that we are worried about in the small infant post OA/TOF repair who may have reflux'* (C35, surgeon, survey).<br>Gastrointestinal infections:<br>*'G.I. infection including NEC later'* (C21, surgeon, survey).<br>*'Sepsis/G.I. infections. There is evidence of increased risk of sepsis in neonates who are given PPIs so I think it would be important to look at this specifically'* (C40, surgeon, survey).<br>*'Gastro [episodes of diarrhoea and vomiting] … because of the lack of acid it [PPI] gives bugs, which will be going into the gut first'* (C39, surgeon, focus group 2).
'Child health related quality of life'	Measure the time that children are hospitalised for (P11; P12, mothers, interviews), child's quality of sleep (P10; P11, mothers, interviews) and child's mental health:<br>'*His mental wellbeing is just as important as his physical wellbeing. In preventing strictures or reducing the severity of a stricture, ultimately, you're reducing hospital stays, you're reducing stress. He developed a fear of hospitals when he was about nine months old. As soon as he would see the entrance, he'd just be an absolute mess'* (P12, mother, interview).

For peer review only - http://bmjopen.bmj.com/site/about/guidelines.xhtml

SUPPLEMENTARY FILE 8 Edits and additions for predefined outcomes

	Measure *'long term outcomes including Barrett's oesophagus'* (C11, surgeon, survey).<br>*'I think longer term follow-up would also be important as a secondary outcome at 5 and 10 years'* (C16, surgeon, survey).<br>Also see the edits or additions in 'Oral intake' and 'Growth' as these are overlapping.
'Maternal Health Related Quality of Life'	Amend to measure *'Parental'* health related quality of life (P12; P13, mothers, interviews):<br>*I don't think it should be limited to mothers, because obviously dads go through the same thing. I think it should be parental health related quality of life, rather than maternal. Actually, I work and my husband stays at home and looks after [Child's name] ... We don't have traditional gender roles at home. There are probably more families like that as well, so it's probably worth including dads, grandparents or whoever it is. That's probably the only thing I'd change is that it was parental rather than maternal* (P12, mother, interview).<br>*Maybe not just maternal, maybe it needs to be parental because it has an effect on certainly both parents* (P13, mother, interview).
'Other complications directly related to oesophageal atresia'	*'Other surgery – aortopexy / tracheopexy'* (C49, surgeon, survey).
'Severity of anastomotic stricture'	*'Anastomotic tension'* (C14, surgeon, survey).<br>Overlapping with 'Respiratory symptoms':<br>*'Any near-death attacks'* (C25, surgeon, survey).<br>*'ALTE'* [apparent life-threatening event/BRUE – brief, resolved, unexplained event] (C47, surgeon, survey).
'Need for treatment of reflux'	*'Fundoplication'* (C47, surgeon, survey)<br>*'Other surgery – fundoplication'* (C49, surgeon, survey).

For peer review only - http://bmjopen.bmj.com/site/about/guidelines.xhtml

COREQ (COnsolidated criteria for REporting Qualitative research) Checklist

A checklist of items that should be included in reports of qualitative research. You must report the page number in your manuscript where you consider each of the items listed in this checklist. If you have not included this information, either revise your manuscript accordingly before submitting or note N/A.

Topic	Item No.	Guide Questions/Description	Reported on Page No.
**Domain 1: Research team and reflexivity**			
Personal characteristics			
Interviewer/facilitator	1	Which author/s conducted the interview or focus group?	
Credentials	2	What were the researcher's credentials? E.g. PhD, MD	
Occupation	3	What was their occupation at the time of the study?	
Gender	4	Was the researcher male or female?	
Experience and training	5	What experience or training did the researcher have?	
Relationship with participants			
Relationship established	6	Was a relationship established prior to study commencement?	
Participant knowledge of the interviewer	7	What did the participants know about the researcher? e.g. personal goals, reasons for doing the research	
Interviewer characteristics	8	What characteristics were reported about the inter viewer/facilitator? e.g. Bias, assumptions, reasons and interests in the research topic	
**Domain 2: Study design**			
Theoretical framework			
Methodological orientation and Theory	9	What methodological orientation was stated to underpin the study? e.g. grounded theory, discourse analysis, ethnography, phenomenology, content analysis	
Participant selection			
Sampling	10	How were participants selected? e.g. purposive, convenience, consecutive, snowball	
Method of approach	11	How were participants approached? e.g. face-to-face, telephone, mail, email	
Sample size	12	How many participants were in the study?	
Non-participation	13	How many people refused to participate or dropped out? Reasons?	
Setting			
Setting of data collection	14	Where was the data collected? e.g. home, clinic, workplace	
Presence of non-participants	15	Was anyone else present besides the participants and researchers?	
Description of sample	16	What are the important characteristics of the sample? e.g. demographic data, date	
Data collection			
Interview guide	17	Were questions, prompts, guides provided by the authors? Was it pilot tested?	
Repeat interviews	18	Were repeat inter views carried out? If yes, how many?	
Audio/visual recording	19	Did the research use audio or visual recording to collect the data?	
Field notes	20	Were field notes made during and/or after the inter view or focus group?	
Duration	21	What was the duration of the inter views or focus group?	
Data saturation	22	Was data saturation discussed?	
Transcripts returned	23	Were transcripts returned to participants for comment and/or	

For peer review only

Topic	Item No.	Guide Questions/Description	Reported on Page No.
		correction?	
**Domain 3: analysis and findings**			
Data analysis			
Number of data coders	24	How many data coders coded the data?	
Description of the coding tree	25	Did authors provide a description of the coding tree?	
Derivation of themes	26	Were themes identified in advance or derived from the data?	
Software	27	What software, if applicable, was used to manage the data?	
Participant checking	28	Did participants provide feedback on the findings?	
Reporting			
Quotations presented	29	Were participant quotations presented to illustrate the themes/findings? Was each quotation identified? e.g. participant number	
Data and findings consistent	30	Was there consistency between the data presented and the findings?	
Clarity of major themes	31	Were major themes clearly presented in the findings?	
Clarity of minor themes	32	Is there a description of diverse cases or discussion of minor themes?	

Developed from: Tong A, Sainsbury P, Craig J. Consolidated criteria for reporting qualitative research (COREQ): a 32-item checklist for interviews and focus groups. *International Journal for Quality in Health Care*. 2007. Volume 19, Number 6: pp. 349 – 357

Once you have completed this checklist, please save a copy and upload it as part of your submission. DO NOT include this checklist as part of the main manuscript document. It must be uploaded as a separate file.

For peer review only
